# A rapid ionic liquid-based DNA extraction method for molecular diagnostics of urinary tract infections

**DOI:** 10.1128/spectrum.03191-25

**Published:** 2026-02-27

**Authors:** Johanna Kreuter, Lena Piglmann, Katarina Priselac, Roland Martzy, Michael Ante, Dominik Walter, Ildiko-Julia Pap, Barbara Ströbele, Andreas H. Farnleitner, Georg H. Reischer, Claudia Kolm

**Affiliations:** 1 TU Wien, Institute of Chemical, Environmental and Bioscience Engineering, Research Unit Molecular Diagnostics (IFA-Tulln), Tulln, Austria; 2 ICC Interuniversity Cooperation Centre Water & Health, Vienna, Austria; 3 SAN Group GmbH 723891 , Herzogenburg, Austria; 4 Institute of Hygiene and Microbiology, University Hospital St. Pölten, St. Pölten, Austria; 5 Karl Landsteiner University of Health Sciences 467773, Krems an der Donau, Austria; 6 Department of Water Quality and Health, Karl Landsteiner University of Health Sciences 467773, Krems an der Donau, Austria; 7 TU Wien, Institute of Chemical, Environmental and Bioscience Engineering, Research Group for Microbiology and Molecular Diagnostics, Vienna, Austria; End TB Dx Consulting LLC, San Diego, California, USA

**Keywords:** urinary tract infections, DNA extraction, rapid method, uropathogens, molecular detection, extraction method comparison

## Abstract

Rapid and reliable DNA extraction from urine is a critical bottleneck in advancing molecular diagnostics for urinary tract infections (UTIs) in both centralized and decentralized settings. Here, we present an ionic liquid-based DNA extraction method (IL-DEx) that enables recovery of bacterial DNA from urine samples in under 30 min using minimal equipment and no hazardous chemicals. IL-DEx was benchmarked against a widely used commercial kit (QIAamp DNA Mini Kit, QIAGEN) using reference strains, clinical isolates, and spiked urine samples. For gram-negative bacteria, IL-DEx achieved comparable DNA yields (47%–102% relative efficiency), while recoveries from gram-positive bacteria were lower (0.7%–8%) but sufficient for downstream detection. Quantitative PCR (qPCR) revealed linear DNA recovery across five to six orders of magnitude (10⁸–10² CFU/mL, R² > 0.99), with detection limits of ~10²–10³ CFU/mL for gram-negatives and ~10³–10⁴ CFU/mL for gram-positives using 1 mL of urine. Clinical evaluation with 13 patient urine samples (ten culture-positive, three culture-negative) demonstrated that IL-DEx reliably enabled pathogen detection by qPCR and full-length 16S rRNA gene sequencing (Oxford Nanopore). Performance was comparable to three other extraction methods tested head-to-head, including the QIAamp DNA Mini Kit (QIAGEN), the MagaZorb DNA Mini-Prep Kit (Promega), and a phenol-chloroform extraction method. These findings establish IL-DEx as the first ionic liquid-based approach evaluated for DNA recovery from clinical urine samples, providing a fast, simple, and low-cost method suitable for integration into molecular workflows for UTI diagnostics across diverse laboratory and clinical settings.

**IMPORTANCE:**

Urinary tract infections (UTIs) are among the most common infections worldwide and a major driver of antibiotic use. Rapid and accurate diagnosis is critical to guide therapy, reduce inappropriate antibiotic prescriptions, and improve patient outcomes. While molecular diagnostics can drastically reduce time to identify uropathogens, their implementation remains constrained by upstream DNA extraction—a step that is often laborious, cost-intensive, or incompatible with rapid diagnostic workflows. We developed a fast, simple, and low-cost DNA extraction method (IL-DEx) that uses an ionic liquid and magnetic beads to recover bacterial DNA directly from urine. IL-DEx eliminates hazardous reagents and complex equipment while delivering performance comparable to established extraction kits. By streamlining this critical pre-analytical step, IL-DEx enables faster molecular diagnostics and broadens access to modern UTI testing. Its simplicity and robustness position it as a valuable tool for improving diagnostic speed, antimicrobial stewardship, and patient care across healthcare settings.

## INTRODUCTION

Urinary tract infections (UTIs) are among the most prevalent infections worldwide, affecting more than 400 million individuals annually in both community and healthcare settings (1). High-risk populations include infants, pregnant women, the elderly, catheterized patients, and individuals with chronic conditions such as diabetes. Nearly half of all women experience at least one UTI in their lifetime (2). Rising global incidence (3), substantial healthcare costs (4, 5), and increasing antimicrobial resistance (AMR) among uropathogens highlight the need for timely and accurate diagnosis to guide therapy, reduce empirical antibiotic use, and mitigate the spread of AMR (6, 7).

Clinically, UTIs present with a broad spectrum of etiologies and severities, ranging from uncomplicated lower tract infections, such as cystitis, to severe upper tract infections like pyelonephritis, which can progress to urosepsis or septic shock if not promptly treated (8, 9). Uropathogenic *Escherichia coli* is the main causative agent, responsible for 65%–75% of cases, followed by *Klebsiella pneumoniae*, *Proteus mirabilis*, *Pseudomonas aeruginosa*, *Enterococcus* spp., and *Staphylococcus* spp., depending on patient demographics and healthcare settings (10). Current diagnostics rely primarily on urine culture, which requires 18–30 h for species identification and an additional 18–24 h for antimicrobial susceptibility testing (11). While bacterial growth of 10^4^–10^5^ CFU/mL is usually considered indicative of a UTI, lower diagnostic thresholds (10²−10⁴ CFU/mL) can be clinically relevant in specific populations such as men, pregnant women, or catheterized individuals (12, 13). However, the diagnosis is not always straightforward. Despite being the clinical gold standard, urine culture suffers from limited diagnostic sensitivity (e.g., ~60% for detecting acute UTI (14)) and often fails to detect fastidious or polymicrobial infections (15–17).

These limitations have spurred the development of molecular diagnostic tools that provide rapid, sensitive, and often species-level detection of uropathogens and resistance genes directly from urine (18). Emerging platforms include real-time multiplexed PCR assays (18, 19) and isothermal amplification assays such as loop-mediated isothermal amplification, which are being explored for decentralized testing (20–22). Likewise, both targeted (e.g., 16S-23S rRNA gene) (14, 23) and untargeted (shotgun metagenomics) (24) next-generation sequencing approaches are gaining traction as sequencing costs decline and bioinformatics tools mature. For instance, portable sequencers such as the MinION (Oxford Nanopore Technologies) have demonstrated near real-time identification of pathogens and resistance genes in urine in under 5 h (25).

Despite these advances, all molecular platforms remain dependent on upstream nucleic acid extraction—a critical yet often overlooked step that impacts analytical performance, cost, and turnaround time (26). Conventional extraction methods are often labor-intensive, expensive, or poorly suited for rapid or decentralized workflows. To address this, we previously developed a simplified ionic liquid-based extraction protocol that combines chemical lysis with magnetic bead-based purification (27). The method uses 1-ethyl-3-methylimidazolium acetate ([C_2_mim][OAc]) to achieve simultaneous bacterial cell lysis and nucleic acid binding, enabling DNA and RNA recovery from periopathogenic bacterial cultures within 30 min (27). However, its performance on uropathogens and in urine—a matrix characterized by variable microbial loads, host DNA background, and inhibitory compounds —remained untested.

In this proof-of-concept study, we adapted and evaluated the ionic liquid-based extraction method (IL-DEx) for the recovery of bacterial DNA from urine to support rapid molecular detection of uropathogens. IL-DEx was tested across a range of diagnostically relevant specimen types, including (i) cultured reference strains and clinical isolates, (ii) spiked artificial and native urine from healthy donors, and (iii) clinical urine specimens from patients with suspected UTIs. Benchmarking was performed against a widely used commercial kit (QIAamp DNA Mini Kit, Qiagen) and, for clinical specimens, extended to include a second commercial kit (MagaZorb DNA Mini-Prep Kit, Promega) and a traditional phenol-chloroform extraction method. Through this comparative framework, we aimed to assess the analytical performance and diagnostic utility of IL-DEx for integration into molecular workflows for UTI diagnostics.

## MATERIALS AND METHODS

### Bacterial strains and isolates used in this study

This study included seven clinically relevant bacterial species commonly found in UTIs: *E. coli* (type strain NCTC 9001; clinical isolate IHM 5531), *P. aeruginosa* (reference strain NCTC 10662; isolate IHM 5716), *K. pneumoniae* (type strain DSM 30104; isolate IHM 5571), *P. mirabilis* (type strain DSM 4479; isolate IHM 5533), *Enterococcus faecalis* (type strain DSM 20478; isolate IHM 5490), *Enterococcus faecium* (type strain DSM 20477; isolate IHM 5458), and *Staphylococcus saprophyticus* (type strain DSM 20229; isolate IHM 5493). Isolates were obtained from urine samples of patients with confirmed or suspected urinary tract infections at University Hospital St. Pölten (Austria).

Gram-negative bacteria were cultured overnight in lysogeny broth (Merck, Germany) at 37°C, with shaking at 170 rpm. Gram-positive bacteria were grown under identical conditions in tryptic soy broth (Merck). Overnight cultures were diluted in fresh medium to an optical density at 600 nm (OD_600_) of 0.1 and incubated to reach an OD_600_ of ~1.0 prior to use in downstream experiments.

### Experiments with pure bacterial cultures

Cells from actively growing cultures of *E. coli*, *P. aeruginosa*, *K. pneumoniae*, *P. mirabilis*, *E. faecalis*, *E. faecium*, and *S. saprophyticus* were harvested at an OD_600_ of ~1.0. Cultures were centrifuged for 5 min at 4,000 × *g*, washed once, and resuspended in isotonic saline solution (0.9% NaCl) to the same OD_600_. For each strain, 10 µL of the resulting cell suspension was used for DNA extraction with the IL-DEx and the QIAamp DNA Mini Kit (QIAGEN, Germany). Extractions were performed in triplicate for each method. Negative controls using saline were included to monitor for contamination.

Total cell count per milliliter (TCC/mL) was determined by epifluorescence microscopy. For this, bacterial cell suspensions were fixed with sterile-filtered paraformaldehyde (final concentration 0.8%) overnight at 4°C and then filtered through 0.2-µm Anodisc 25 filters (Whatman, Germany). Filters were stained with SYBR Gold (30 µL of 1:400 dilution in sterile deionized water; Invitrogen, Germany), incubated in the dark for 15 min, rinsed three times with sterile Milli-Q water, and air-dried in the dark. Filters were mounted on microscope slides using anti-fade mounting solution (Citifluor). Slides were examined with immersion oil using a Nikon Eclipse Ni microscope equipped with a Nikon DS-Qi2 camera at 400× or 1,000× magnification (Ex ~470, Em ~515). TCC/mL was calculated based on the average number of cells counted at 10 randomly selected positions on the filter (10 × 100 mm²) and the volume of the sample filtered.

### Spiking experiments with artificial urine and urine from healthy donors 

Actively growing cultures of *E. coli* NCTC 9001, *P. aeruginosa* NCTC 10662, *K. pneumoniae* DSM 30104, *P. mirabilis* DSM 4479, *E. faecalis* DSM 20478, *E. faecium* DSM 20477, and *S. saprophyticus* DSM 20229 with an OD_600_ of ~1.0 were harvested by centrifugation (5 min, 4,000 × *g*), washed, and resuspended in saline to an OD_600_ of ~2.0. From each suspension, six 10-fold serial dilutions (from 1:10 to 1:10^6^) were prepared. Each dilution and the undiluted cell suspension were spiked into artificial urine (Sigmatrix Urine Diluent, Sigma-Aldrich, USA) at a 1:10 ratio (vol/vol). A 1 mL aliquot of each spiked sample was centrifuged at 10,000 × *g* for 10 min at 4°C, and the pellet was subjected to DNA extraction using both IL-DEx and the QIAamp DNA Mini Kit (QIAGEN). Unspiked artificial urine was processed in parallel as a negative extraction control. All extractions were performed in triplicate for each dilution and method. For bacterial quantification, the three highest dilutions were used to determine colony-forming units per mL (CFU/mL).

CFU/mL values were obtained by plating the respective cell dilution on lysogeny broth agar (Merck) in triplicate and incubating at 37°C overnight. The number of colonies was counted and used for the calculation of CFU/mL. As a negative control, isotonic saline solution was plated.

Spiking experiments with urine from healthy donors were performed similarly. Freshly cultivated *E. coli* or *E. faecalis* cells were spiked to an OD_600_ of ~0.02 into native first-void urine samples from healthy donors. A 1 mL aliquot of each spiked and unspiked urine was centrifuged at 10,000 × *g* for 10 min at 4°C, and the resulting pellet was subjected to DNA extraction using both IL-DEx and the QIAamp DNA Mini Kit. To assess the DNA content of the spike, the cell suspension was also spiked into saline and processed in parallel. Negative extraction controls with only saline were included. All extractions were performed in triplicate per method.

### Clinical specimens

13 clean-catch midstream urine samples from patients with suspected UTI were processed for comparative analysis of extraction methods: ten were urine culture-positive (>10^4^ CFU/mL), and three were culture-negative (<10^4^ CFU/mL). Samples were collected in sterile containers and stored at 4°C for no longer than 5 days prior to DNA extraction (see Table 1 and Fig. S6) according to consensus recommendations for urinary microbiome research guidelines (28).

**TABLE 1:** Clinical metadata of urine samples for extraction and analysis

Sample no.	Urine appearance	Determined bacterial count by urine culture	UTI suspected	Identified uropathogen by MALDI-TOF	Storage time until extraction (days)
1	Clear	>10^4^	Yes	*Citrobacter koseri*	5
2	Bloody	>10^4^	Yes	*E. coli*	5
3	Bloody, turbid	>10^4^	Yes	*E. coli*	4
4	Clear	>10^4^	Yes	*E. coli*	4
5	Slightly turbid	>10^4^	Yes	*E. coli*	5
6	Turbid	>10^4^	Yes	*E. coli*	4
7	Slightly turbid	>10^4^	Yes	*K. pneumoniae*	4
8	Highly turbid	>10^4^	Yes	*E. coli*	4
9	Turbid	>10^4^	Yes	*E. coli*	3
10	Slightly turbid	>10^4^	Yes	*P. aeruginosa*	3
11	Clear	<10^4^	No	*–^a^*	2
12	Slightly turbid	<10^4^	No	*–^a^*	2
13	Slightly turbid	<10^4^	No	*–^a^*	2

^*a*^ – indicates that urine culture was negative and MALDI-TOF was not performed.

Each sample was processed in duplicate using four different DNA extraction methods: IL-DEx, QIAamp DNA Mini Kit (QIAGEN), MagaZorb DNA Mini-Prep Kit (Promega, USA), and an in-house protocol based on mechanical bead-beating and phenol-chloroform extraction (PC). For each replicate, 1 mL of urine was centrifuged at 10,000 × *g* for 10 min at 4°C, and the pellet was subjected to DNA extraction. Negative extraction controls were included in duplicates for all methods.

Clinical samples were also observed by epifluorescence microscopy. For this, urine samples were diluted 1:10, filtered through 0.2-µm filters (Anodisc 25), and stained with SYBR Gold. Staining and imaging were performed as described above.

### Ionic liquid-based DNA extraction

The ionic liquid (IL) [C_2_mim][OAc] (1-ethyl-3-methylimidazolium acetate; purity >95%; CAS 143314-17-4) was purchased from Iolitec (Germany) and used as a 90% (wt/wt) solution in 10 mM Tris pH 8 buffer. The solution was prepared by weighing the required amount of IL and Tris buffer.

For extraction, 10 µL of the respective sample or the cell pellet was mixed with 90 µL of 90% [C_2_mim][OAc] and incubated at 95°C for 5 min. The resulting lysate was mixed with 150 µL of SeraSil-Mag 400 silica-coated superparamagnetic beads (Cytiva, USA). Subsequently, 765 µL of 10 mM Tris pH 8 buffer was added to dilute the lysate, and the mixture was vortexed briefly and incubated on a thermomixer (24°C, up to 10 min, 1,400 rpm) to facilitate nucleic acid binding to the beads. Beads were collected by placing the reaction tubes in a magnetic separation rack for 30–60 s. After discarding the supernatant, the beads were washed with 500 µL of 70% ethanol prepared in 10 mM Tris pH 8 buffer, then air-dried at room temperature. Elution was performed by adding 100 µL of TE buffer (10 mM Tris-HCl, 1 mM EDTA, pH 8.0) and incubating on a thermomixer (65°C, 3 min, 1,400 rpm).

### Reference extractions

DNA was extracted using the QIAamp DNA Mini Kit (QIAGEN, Germany), following the manufacturer’s protocol for gram-negative or gram-positive bacteria for cultured samples, and the protocol for gram-positive bacteria for spiked urine samples.

For experiments with clinical samples, DNA was extracted with a bead-beating and phenol-chloroform extraction protocol (PC), the QIAamp DNA Mini Kit (QIAGEN), and the MagaZorb DNA Mini-Prep Kit (Promega), both according to the manufacturers’ instructions for gram-positive bacteria. Elution and resuspension volumes for experiments with clinical urine were adjusted to 100 µL for all methods. Extraction with bead-beating and phenol-chloroform was performed as previously described (29–31). In short, the pellet was resuspended in 0.9% NaCl, and cell lysis was achieved by adding CTAB buffer, phenol, chloroform/isoamyl alcohol, and glass beads in a FastPrep 24 bench-top homogenizer (MP Biomedicals Inc., USA) at a speed setting of 6 m/s for 30 s. DNA was precipitated with isopropanol and washed with ethanol. The dried DNA was resuspended in 100 µL of 10 mM Tris pH 8.0 buffer.

### Quantification of DNA using quantitative PCR

Total bacterial DNA was quantified using quantitative PCR (qPCR) targeting the V1–V2 region of the 16S rRNA gene, with primers universally conserved across bacterial taxa (denoted as 16S-qPCR) (32). *E. coli* DNA was quantified with a qPCR assay targeting the 23S rRNA gene (denoted as *E. coli*-qPCR) (33). *E. faecalis* and *E. faecium* DNA were quantified with a qPCR assay targeting the *Enterococcus* 23S rRNA gene (denoted as *Enterococcus*-qPCR) (34, 35). Human DNA was quantified with qPCR targeting the human *Alu* repeats (denoted as human*-*qPCR) (36). All qPCR reactions were carried out in a total reaction volume of 15 µL containing oligonucleotides, 7.5 µL qPCR Master Mix, and 2.5 µL of template DNA. Amplification was performed on a qTOWER³ G real-time thermocycler (Analytik Jena, Germany). Detailed information on qPCR assays is provided in Tables S21 and S22. Unless otherwise stated, qPCR reactions were carried out in duplicates. Calibration curves were generated using a dilution series of plasmid DNA containing a known number of target gene copies, quantified via PicoGreen measurements. Calibration curves for human-qPCR were generated using a dilution series of human genomic DNA (TaqMan Control Genomic DNA, Applied Biosystems, USA) containing a known mass of genomic DNA. To rule out PCR inhibition, samples were measured in multiple dilutions. No template controls were included in each qPCR run.

### Oxford nanopore full-length 16S rRNA sequencing

The 16S rRNA gene was amplified using modified versions of the 27F and 1492R primers (Table S22), respectively, yielding a near full-length 16S amplicon with an approximate length of 1,500 bp. The amplification was performed with the LongAmpTaq HotStart 2x MasterMix (New England Biolabs, USA) in a total reaction volume of 25 µL. The amplicons were purified using the ProNex Size-Selective Purification System (Promega, USA), applying a 1.5-fold ratio of beads-to-sample volume for an approximate size cutoff at 250 bp. The subsequent library preparation was conducted using the Native Barcoding Kit 96 V14 (Oxford Nanopore Technologies, UK), following the manufacturer’s instructions and the recommended amplicon input amount of 200 fmol per sample. The final library was sequenced on a MinION Mk1B platform using an R10.4.1 flow cell (Oxford Nanopore Technologies) for 24 h. The Nanopore sequencing run and its base calling were checked with NanoPlot (37). In case a sample exceeded a yield of 20,000 reads, the raw reads were down-sampled to 20,000 reads using rasusa (38). Subsequently, the reads were processed with Chopper (37), retaining only those that were between 500 and 2,000 bp long. The read statistics were computed with NanoStats (37). Taxonomic classification was carried out with emu (v3.5.0) (39) using the standard emu database (v.3.4.5; a combination of rrnDB v5.6 (40) and NCBI 16S RefSeq (41)). Read statistics and emu classifications per sample were collected with Python.

### Nucleic acid quantity, purity, and fragmentation

Total nucleic acid concentration (ng/mL urine) and purity (A260/280 and A260/230 ratios) were measured via absorbance using a NanoDrop One/OneC Spectrophotometer (Thermo Fisher Scientific, USA). Total DNA concentration (ng/mL urine) was measured with the Qubit dsDNA HS Assay Kit (Invitrogen). DNA fragmentation was assessed by agarose gel electrophoresis. Briefly, a 1% TBE agarose gel was loaded with 10 µL of extracts and 5 or 10 µL of 1 kB Plus DNA Ladder (New England Biolabs, Germany). DNA was stained with SYBR Gold (Thermo Fisher Scientific) and visualized using a Gel Doc XR+ system (Bio-Rad, USA).

### Statistical analysis

Data analysis and visualization were performed using Microsoft Excel, GraphPad Prism (v10; GraphPad Software), and R (version 4.3.3; RStudio). For spiking experiments, paired *t*-tests were used to compare IL-DEx and QIAGEN in unspiked and spiked urine samples, as well as for *E. coli* and *E. faecalis* spike-only controls. For clinical specimens, total nucleic acid and DNA yields were analyzed using repeated measures one-way ANOVA with Geisser-Greenhouse correction to account for violations of sphericity. Tukey’s multiple comparisons test was applied to compare all pairs of extraction methods (α = 0.05). A mixed-design ANOVA was conducted using the ezANOVA() function from the ez package to assess the effects of extraction method (within-subject factor) and sample category (UTI vs non-UTI; between-subject factor) on bacterial DNA yield. Where the assumption of sphericity was violated (as determined by Mauchly’s test), Greenhouse-Geisser correction was applied. Residuals were tested for normality using the Shapiro-Wilk test (W = 0.99, *p* = 0.686). Post hoc pairwise comparisons were performed using the emmeans and nlme packages, with *P* values adjusted using Tukey’s HSD method. Correlations between 16S and 23S rRNA gene copy numbers were evaluated using Spearman’s rank correlation. The association between *E. coli* urine culture status (culture positive vs culture negative) and *E. coli*-specific qPCR (qPCR-positive vs qPCR-negative, threshold > 3 log_10_ 23S copies per extract) was tested using Fisher’s exact test. Statistical significance was defined as *p* < 0.05 (two-tailed). qPCR data were analyzed using qPCRsoft 4.0 (Analytik Jena). Schematic figures were created with BioRender under academic license j43g432 (A.H.F.).

## RESULTS

### IL-DEx workflow

**Fig 1:**
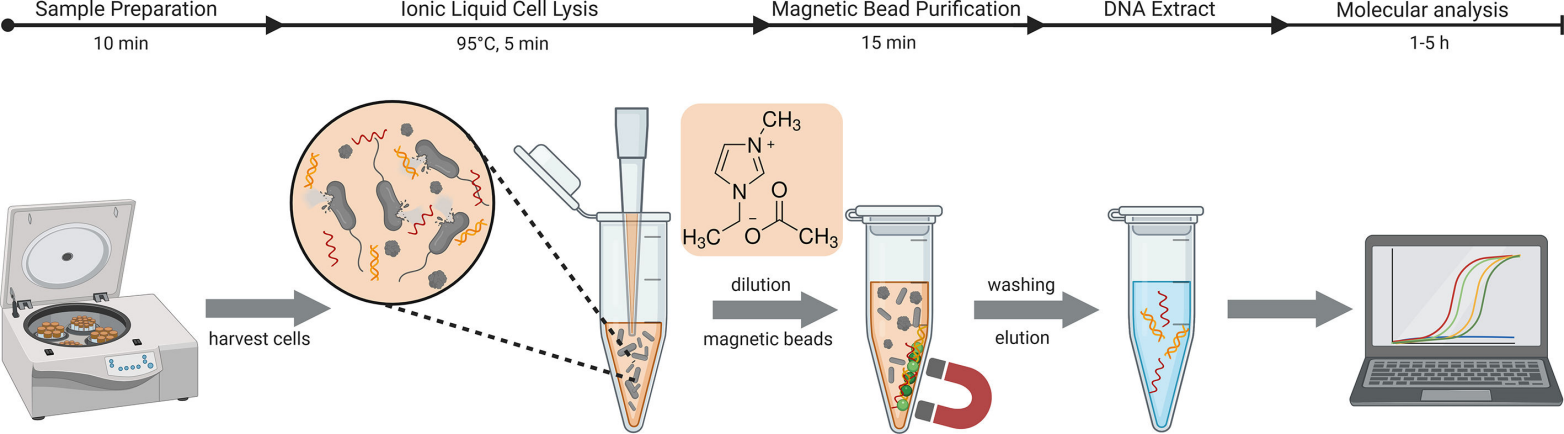
Schematic illustration of the IL-DEx workflow for rapid bacterial DNA extraction from urine samples. The protocol comprises four steps: (i) centrifugation to harvest bacterial cells, (ii) resuspension of the pellet and ionic liquid-mediated lysis of cells using [C_2_mim][OAc] to release genomic DNA, (iii) DNA capture and purification with silica-coated magnetic beads, with [C_2_mim][OAc] also serving as the DNA binding buffer, and (iv) elution of purified DNA for (v) downstream molecular analyses, such as PCR and sequencing. Approximate processing times are indicated above the workflow.

The IL-DEx workflow was designed to enable rapid DNA extraction from urine using a single-tube lysis and nucleic acid capture protocol (Fig. 1). The method combines chemical lysis with 1-ethyl-3-methylimidazolium acetate ([C₂mim][OAc]) and magnetic bead-based recovery. DNA is eluted in less than 30 min without the need for hazardous organic solvents. The streamlined setup requires only standard laboratory equipment, including a centrifuge, heating block, and a magnetic rack.

### DNA recovery from bacterial reference strains and clinical isolates

We first evaluated the extraction efficiency of IL-DEx using pure cultures of seven clinically relevant uropathogens: *E. coli, K. pneumoniae, P. aeruginosa, P. mirabilis, E. faecalis, E. faecium*, and *S. saprophyticus*. Fresh cell suspensions were prepared from both a reference strain and a clinical isolate obtained from UTI patients and extracted. DNA yields were determined by quantifying 16S rRNA gene copy numbers, applying a universal qPCR assay (16S-qPCR) across all extracts to minimize assay-specific variability in analytical sensitivity. Extraction efficiencies were calculated by comparing the DNA yields to those obtained using the QIAamp DNA Mini Kit (QIAGEN), which served as a benchmark. The QIAGEN kit was assigned 100% efficiency based on preliminary data showing strong concordance between detected genome copy numbers and bacterial cell counts (Table S23).

For gram-negative species (*E. coli*, *K. pneumoniae*, *P. aeruginosa*, *P. mirabilis*), IL-DEx yielded DNA amounts comparable to the QIAGEN kit, with relative extraction efficiencies ranging from 47% to 102% (Table 2). No considerable differences were observed between reference strains and clinical isolates. In contrast, extraction efficiencies from gram-positive bacteria were lower, ranging from 0.7% to 8% relative to the QIAGEN kit, likely due to the increased resistance of their thicker cell walls to chemical lysis (42). Nonetheless, IL-DEx consistently recovered sufficient DNA for downstream analysis, averaging ~10^7^ 16S rRNA gene copies from 10 µL of cultures with optical densities (OD_600_) near 1. Notably, clinical isolates of *E. faecalis* and *S. saprophyticus* showed higher recoveries (8% and 3%, respectively) than their respective reference strains (0.8% and 0.7%).

**TABLE 2:** Total bacterial DNA yield (expressed as 16S rRNA gene copies) extracted from gram-negative and gram-positive uropathogens using the IL-DEx protocol (ID-DEx) and the QIAamp DNA Mini Kit (QIAGEN)*^f^*

Strain^a^	OD_600_^*b*^	Method	Total bacterial DNA extracted^*c*^ (mean 16S rRNA gene copies ± SD)	RSD (%)^*d*^	Extraction efficiency (%)^*e*^
*E. coli* NCTC 9001	1.1	IL-DEx	1.12 × 10^9^ ± 2.12 × 10^8^	19	85
		QIAGEN	1.32 × 10^9^ ± 8.45 × 10^7^	6	100
*E. coli* IHM 5531	0.8	IL-DEx	3.37 × 10^8^ ± 6.93 × 10^7^	21	85
		QIAGEN	3.95 × 10^8^ ± 8.23 × 10^7^	21	100
*P. aeruginosa* NCTC 10662	1.0	IL-DEx	7.04 × 10^8^ ± 1.42E × 10^8^	20	102
		QIAGEN	6.88 × 10^8^ ± 6.56 × 10^7^	10	100
*P. aeruginosa* IHM 5716	0.7	IL-DEx	3.73 × 10^8^ ± 8.69 × 10^7^	23	62
		QIAGEN	6.03 × 10^8^ ± 9.27 × 10^7^	15	100
*K. pneumoniae* DSM 30104	1.0	IL-DEx	8.49 × 10^8^ ± 1.08 × 10^8^	13	56
		QIAGEN	1.53 × 10^9^ ± 1.71 × 10^8^	11	100
*K. pneumoniae* IHM 5571	0.8	IL-DEx	2.17 × 10^8^ ± 5.16 × 10^7^	24	47
		QIAGEN	4.62 × 10^8^ ± 1.67 × 10^7^	4	100
*P. mirabilis* DSM 4479	1.1	IL-DEx	1.52 × 10^9^ ± 2.53 × 10^8^	17	90
		QIAGEN	1.69 × 10^9^ ± 3.02 × 10^8^	18	100
*P. mirabilis* IHM 5533	0.7	IL-DEx	4.49 × 10^8^ ± 5.24 × 10^7^	12	78
		QIAGEN	5.74 × 10^8^ ± 7.87 × 10^7^	14	100
*E. faecalis* DSM 20478	1.1	IL-DEx	9.31 × 10^6^ ± 8.61 × 10^5^	9	0.8
		QIAGEN	1.23 × 10^9^ ± 3.81 × 10^8^	31	100
*E. faecalis* IHM 5490	0.7	IL-DEx	3.49 × 10^7^ ± 1.54 × 10^7^	44	8.3
		QIAGEN	4.18 × 10^8^ ± 8.66 × 10^7^	21	100
*E. faecium* DSM 20477	1.3	IL-DEx	2.52 × 10^7^ ± 2.16 × 10^6^	9	3
		QIAGEN	8.56 × 10^8^ ± 3.07 × 10^8^	36	100
*E. faecium* IHM 5458	0.8	IL-DEx	8.74 × 10^6^ ± 1.68 × 10^6^	19	2
		QIAGEN	4.22 × 10^8^ ± 1.84 × 10^7^	4	100
*S. saprophyticus* DSM 20229	1.1	IL-DEx	3.89 × 10^6^ ± 4.00 × 10^4^	1	0.7
		QIAGEN	5.31 × 10^8^ ± 3.20 × 10^7^	6	100
*S. saprophyticus* IHM 5493	0.8	IL-DEx	7.53 × 10^6^ ± 5.28 × 10^5^	7	3
		QIAGEN	2.21 × 10^8^ ± 4.01 × 10^7^	18	100

^*a*^ The panel includes both reference strains and clinical isolates (denoted as “IHM”), all freshly cultured and processed in triplicate. DNA was extracted from 10 µL of bacterial suspension per replicate.

^*b*^ OD_600_ refers to the optical density of the bacterial cell suspension used for extraction.

^*c*^ Data are reported as mean 16S rRNA gene copies ± standard deviation (SD) from three biological replicates.

^*d*^ Relative standard deviation (RSD, %) reflects inter-replicate variability.

^*e*^ Extraction efficiency (%) represents the DNA yield recovered with IL-DEx relative to the QIAGEN kit, which was defined as 100%.

^*f*^ Full raw data are available in Table S1.

### DNA recovery from spiked urine samples

To assess extraction performance in urine matrices, we conducted spiking experiments in (i) artificial urine (AU), which lacks background DNA, and (ii) native urine from healthy donors, which contains both host and microbial DNA.

#### Spiking experiments in artificial urine

AU experiments allowed direct comparison of extraction efficiency and detection sensitivity between IL-DEx and the QIAamp DNA Mini Kit (QIAGEN) under well-defined conditions. For each of the seven tested uropathogens, serial dilutions of fresh bacterial suspensions were prepared in AU at final concentrations ranging from ~10^8^ to ~10^2^ CFU/mL. One-milliliter aliquots were extracted in triplicate using both IL-DEx and the QIAGEN kit. DNA yields were quantified by qPCR targeting the 16S rRNA gene.

**Fig 2:**
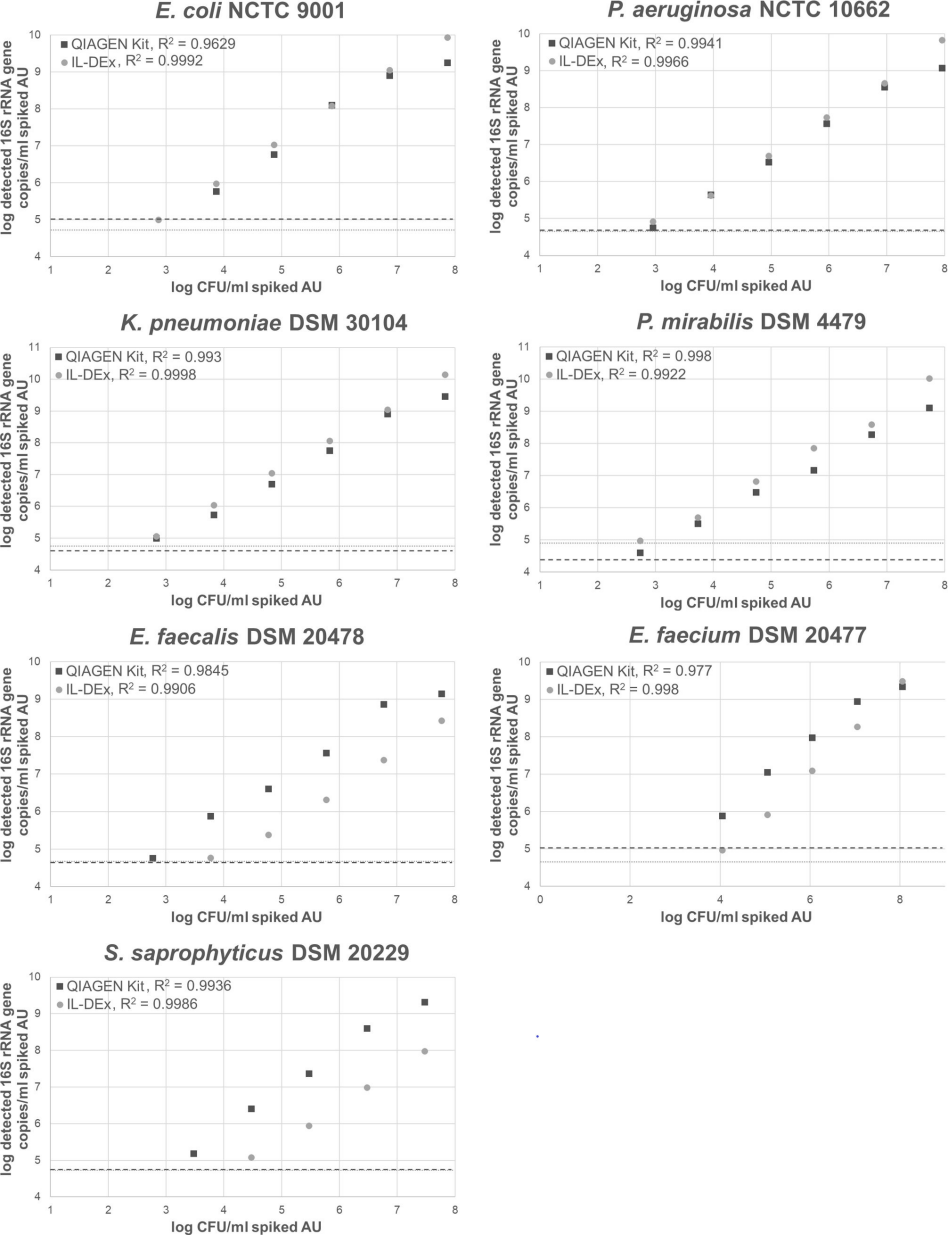
Spiking experiments in artificial urine (AU) to assess linearity and the detection limit of the IL-DEx workflow. Plots show log-transformed 16S rRNA gene copies recovered from 1 mL spiked AU, containing ~10^8^ to ~10^2^ CFU/mL. Each dilution was extracted in triplicate with IL-DEx (light gray filled circles) and the QIAGEN kit (dark gray filled squares). Data points represent means. Standard deviations are not shown due to log-scale compression and close overlap between IL-DEx and QIAGEN data, particularly for gram-negative strains; replicate variability is reported in Tables S2 to S8. Dashed and dotted lines indicate background 16S rRNA gene levels detected in extraction controls (unspiked AU) (dashed lines, 16S background extraction control QIAGEN kit; dotted lines, 16S background extraction control IL-DEx). A three-sigma threshold was chosen to exclude all data points that lie within three standard deviations of the control mean.

As shown in Fig. 2, IL-DEx yielded linear DNA recovery across all concentrations and strains tested (R² > 0.99). In contrast, the QIAGEN kit showed signs of saturation at higher bacterial loads, particularly for gram-negative species, suggesting column overloading. *P. mirabilis* was the exception, for which both methods remained linear across all concentrations. For gram-negative species, IL-DEx yielded DNA amounts comparable to the QIAamp kit. For gram-positive species, IL-DEx yielded approximately 1–2 log_10_ fewer 16S rRNA gene copies per mL.

The estimated detection limit for IL-DEx was ~10³ CFU/mL for gram-negatives and ~10⁴ CFU/mL for gram-positives, based on 16S rRNA gene quantification. However, assay sensitivity was ultimately constrained by low-level background amplification in 16S-qPCR, a phenomenon previously attributed to trace *E. coli* DNA contamination in recombinant polymerases or other laboratory reagents, including extraction kits (43). This background was evident in extraction controls and defined the detection threshold (dotted and dashed lines in Fig. 2; 3σ cutoff). To improve detection sensitivity, species-specific qPCR assays targeting the 23S rRNA gene were applied for *E. coli*, *E. faecalis*, and *E. faecium*. These assays confirmed lower detection limits of ~10² CFU/mL for *E. coli* and ~10³ CFU/mL for the two *Enterococcus* species (Fig. S1).

#### Spiking experiments in native urine from healthy donors

To simulate sample complexity—including both human host and microbial DNA background—and assess potential matrix effects, first-void urine from three healthy female and two healthy male donors was spiked with uropathogenic *E. coli* (isolate IHM 5531). The human urinary tract above the urinary sphincter is not sterile and contains an indigenous urinary microbiome (urobiome) (44, 45). Additionally, midstream, clean catch urine contains the urethral and perigenital flora (17). Preliminary qPCR screening of unspiked urine revealed background 16S rRNA gene levels of ~10⁸ copies/mL in female donors and ~10⁷ copies/mL in male donors. Spiking was therefore performed at ~10^9^ 16S rRNA gene copies/mL (corresponds to 10^7^ *E. coli* CFU/mL urine) to ensure detection above the background. All samples were processed using both IL-DEx and the QIAGEN kit. In addition, unspiked urine was assessed for bacterial and host DNA background by 16S-qPCR and human-qPCR.

**Fig 3:**
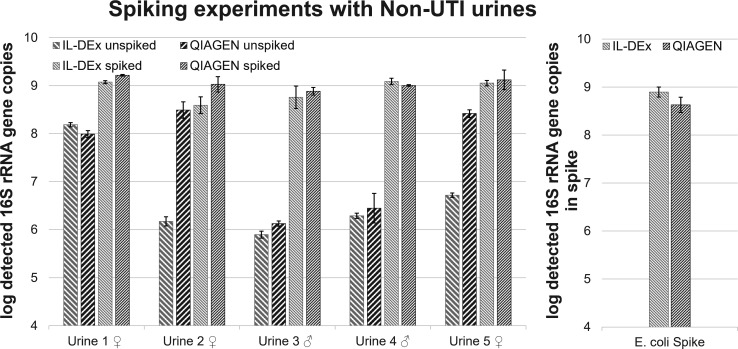
Log-transformed 16S rRNA gene copies detected in extracts from 1 mL spiked and unspiked urine (left panel) and from the *E. coli* cell suspension that was used for spiking (spike control) (right panel). DNA was extracted using IL-DEx (light gray) and the QIAGEN kit (dark gray). Urines were spiked to approx 10^7^ CFU/mL. Data represent mean values from three biological replicates, whiskers indicate the standard deviation. In unspiked urine (left panel), QIAGEN yielded slightly higher 16S rRNA gene copy numbers than IL-DEx (mean paired difference = 0.81 log₁₀ units, 95% CI −0.59 to 2.21, *p* = 0.185), and a similar non-significant trend was observed in spiked urine (mean paired difference = 0.14 log₁₀ units, 95% CI −0.10 to 0.38, *p* = 0.184) (Fig. S3, left panel). For the *E. coli* spike-only control, a paired *t*-test did not detect a statistically significant difference between methods (ns, *p* = 0.104), indicating broadly comparable DNA recovery in the absence of urine matrix (bacterial spike suspension only) (Fig. S3, right panel). Raw data are provided in Table S12.

Both extraction methods enabled robust detection of the *E. coli* spike, with IL-DEx yields comparable to QIAGEN (79% mean extraction efficiency compared to the kit) across donor samples (Fig. 3). IL-DEx underperformed in two unspiked female samples (urine nos. 2 and 5), likely reflecting contributions from vaginal flora rich in gram-positive bacteria (e.g., *Lactobacillus*) (46), which are more resistant to lysis. Across donors, paired *t*-tests showed no statistically significant differences in log 16S rRNA gene copy numbers between IL-DEx and QIAGEN in either unspiked or spiked samples (both *p* > 0.05) (Fig. S3). On average, QIAGEN yielded marginally higher values (mean paired difference = 0.81 log₁₀ units in unspiked urine, 95% CI −0.59 to 2.21, *p* = 0.185; 0.14 log₁₀ units in spiked urine, 95% CI −0.10 to 0.38, *p* = 0.184). In contrast, background DNA analysis indicated that IL-DEx recovered slightly more human DNA than QIAGEN in unspiked urine (334 vs 91 ng; Fig. S2), though this difference was not statistically significant (paired *t*-test, *p* = 0.16).

To further assess gram-positive recovery, a spiking experiment was also performed with *E. faecalis* using urine from a female donor. Samples were processed with IL-DEx and the QIAGEN kit and analyzed by *Enterococcus*-specific 23S qPCR (Fig. S4). In unspiked urine, both methods yielded similar background levels (paired *t*-test, ns, *p* > 0.05). However, in the *E. faecalis* spike control and spiked urine sample, IL-DEx recovered ~1–2 log₁₀ fewer *Enterococcus* gene copies than QIAGEN. Comparable reductions were also observed in pure culture and artificial urine spiking experiments, indicating that lower yields are primarily due to the intrinsic extraction efficiency for this gram-positive bacterium rather than matrix effects.

### DNA recovery from clinical urine samples

We next evaluated the performance of IL-DEx on 13 clinical urine specimens (ten culture-positive “UTI” and three culture-negative “non-UTI” samples as control) (Table 1 and Fig. S6). Each sample was processed using four DNA extraction methods: IL-DEx, two commercial kits (QIAGEN and Promega), and an in-house phenol-chloroform protocol with mechanical bead beating. Extraction performance was broadly assessed by measuring total nucleic acid yield (via Nanodrop), total DNA (via Qubit), bacterial DNA (via 16S-qPCR), *E. coli* DNA (via *E. coli*-qPCR), host DNA (via human-qPCR), DNA integrity (via agarose gel), and microbial composition by full-length 16S rRNA gene sequencing. All urine samples were also imaged by epifluorescence microscopy.

#### Nucleic acid yield and purity

**Fig 4:**
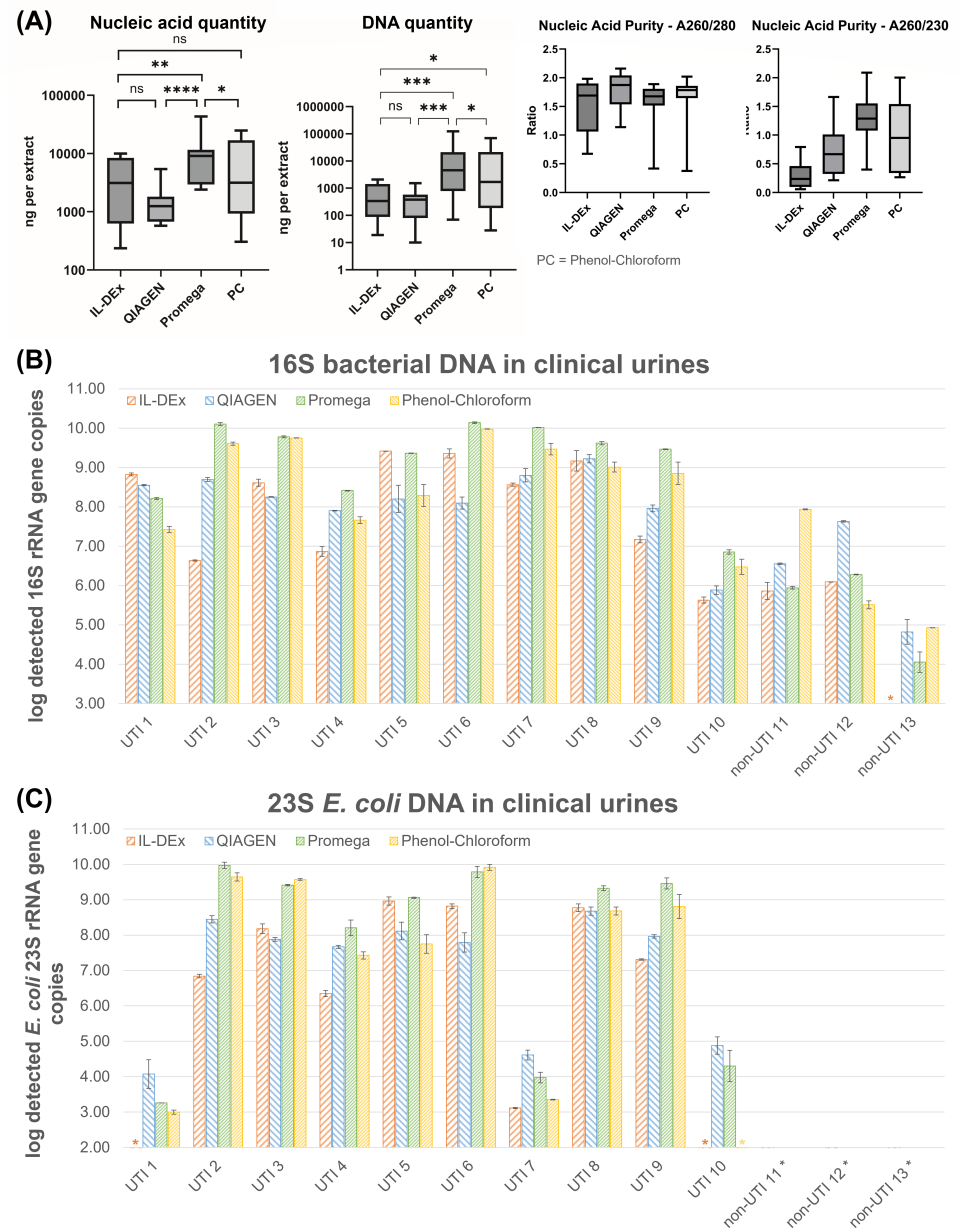
(**A**) Total nucleic acid quantity and purity (measured by Nanodrop), as well as DNA quantity (measured by Qubit) from 13 clinical urine samples extracted with four methods (IL-DEx, QIAGEN, Promega, and phenol–chloroform [PC]). Data are shown as median with min/max values. Methods were compared using repeated measures one-way ANOVA, followed by Tukey’s multiple comparisons test. Significance levels are indicated as *p* < 0.05 (*), *p* < 0.01 (**), *p* < 0.001 (***), *p* < 0.0001 (****). (**B**) and (**C**) Content of total bacterial DNA (as log detected 16S rRNA gene copies) and *E. coli* DNA (as log detected *E. coli* 23S rRNA gene copies) in the DNA extracts from clinical urines obtained via IL-DEx (orange), QIAGEN kit (blue), Promega kit (green), and phenol-chloroform extraction (yellow). Bars show mean values from two biological replicates, with error bars indicating the two measured values. For each replicate, 1 mL of urine was processed. Asterisks indicate results that lie within three standard deviations from the mean of the extraction controls and were therefore excluded. For *E. coli* culture-positive urines (UTI 2–6, 8, and 9), log 23S rRNA gene copy numbers (panel C) strongly correlated with log 16S rRNA gene copy numbers (panel B), as assessed by Spearman’s rank correlation (ρ = 0.92, *p* = 2.8 × 10⁻⁵). Using a threshold of 3 log_10_ 23S rRNA gene copies per extract to define *E. coli* 23S qPCR positivity, detection in IL-DEx extracts was significantly associated with *E. coli* culture status (Fisher’s exact test, *p* = 0.0047). Full raw data are available in Tables S15 to S18.

Across all samples, the Promega kit produced the highest total nucleic acid yield (Nanodrop quantification), significantly exceeding IL-DEx, QIAGEN, and phenol-chloroform (all *p* < 0.05, Fig. 4A, left). QIAGEN yielded significantly less nucleic acid than Promega and phenol-chloroform, while IL-DEx was significantly lower than Promega but not different from QIAGEN or phenol-chloroform. When total DNA (Qubit quantification) was compared (Fig. 4A, middle), the same pattern was observed: Promega achieved the highest DNA yield, followed by phenol-chloroform, IL-DEx, and QIAGEN (repeated-measures ANOVA, *p* < 0.0001). Nucleic acid purity ratios (A260/280) were comparable across methods and within the acceptable range of 1.8–2.0, whereas A260/230 ratios were lower for IL-DEx. Gel electrophoresis of DNA extracts revealed intact high molecular weight DNA in IL-DEx and QIAGEN extracts, while DNA from Promega kit and phenol-chloroform extractions appeared more fragmented (Fig. S7).

#### Bacterial DNA recovery

Bacterial DNA yields (16S rRNA gene copies, Fig. 4B) corresponded well with bacterial cell loads observed by epifluorescence microscopy (Fig. S8). Non-UTI samples and UTI sample no. 10 (low bacterial cell loads) yielded the lowest 16S rRNA gene copies. A mixed-design ANOVA confirmed significant effects of sample category (UTI vs non-UTI, F₁,₂₄ = 36.35, *p* < 0.0001), extraction method (F₃,₇₂ = 3.43, *p* = 0.021), and their interaction (F₃,₇₂ = 6.62, *p* = 0.0005). Among UTI samples, Promega and phenol-chloroform yielded significantly more bacterial DNA than IL-DEx (*p* < 0.0001 and *p* = 0.0228, respectively), while IL-DEx and QIAGEN did not differ (*p* = 0.93). For non-UTI samples, no significant differences between methods were detected (all *p* > 0.1). Pairwise comparisons further showed IL-DEx yielded significantly more bacterial DNA in UTI than in non-UTI samples (Δ = 5.9 log_10_ gene copies, *p* = 0.0001).

#### *E. coli*-specific DNA detection

*E. coli*-qPCR confirmed the presence of *E. coli* in all culture-positive *E. coli* urine samples (UTI sample nos. 2–6, 8, and 9; Fig. 4C and Table 1). Detection of the 23S rRNA gene was significantly associated with *E. coli* culture status (Fisher’s exact test, *p* = 0.0047), confirming the assay’s diagnostic specificity. In these culture-positive samples, 23S rRNA gene copy numbers also closely matched total bacterial DNA levels (16S rRNA gene copies, Fig. 4B), with a strong correlation between 16S and 23S rRNA log copies (Spearman’s ρ = 0.92, *p* = 2.8 × 10⁻⁵). Low-level 23S signals were also detected in samples 1, 7, and 10.

#### Host DNA recovery

Human DNA recovery mirrored bacterial DNA extraction trends, where extraction methods with higher bacterial DNA yields (Promega, phenol-chloroform) also extracted higher amounts of host DNA (Fig. S9).

#### Microbial profiling via nanopore sequencing

**Fig 5:**
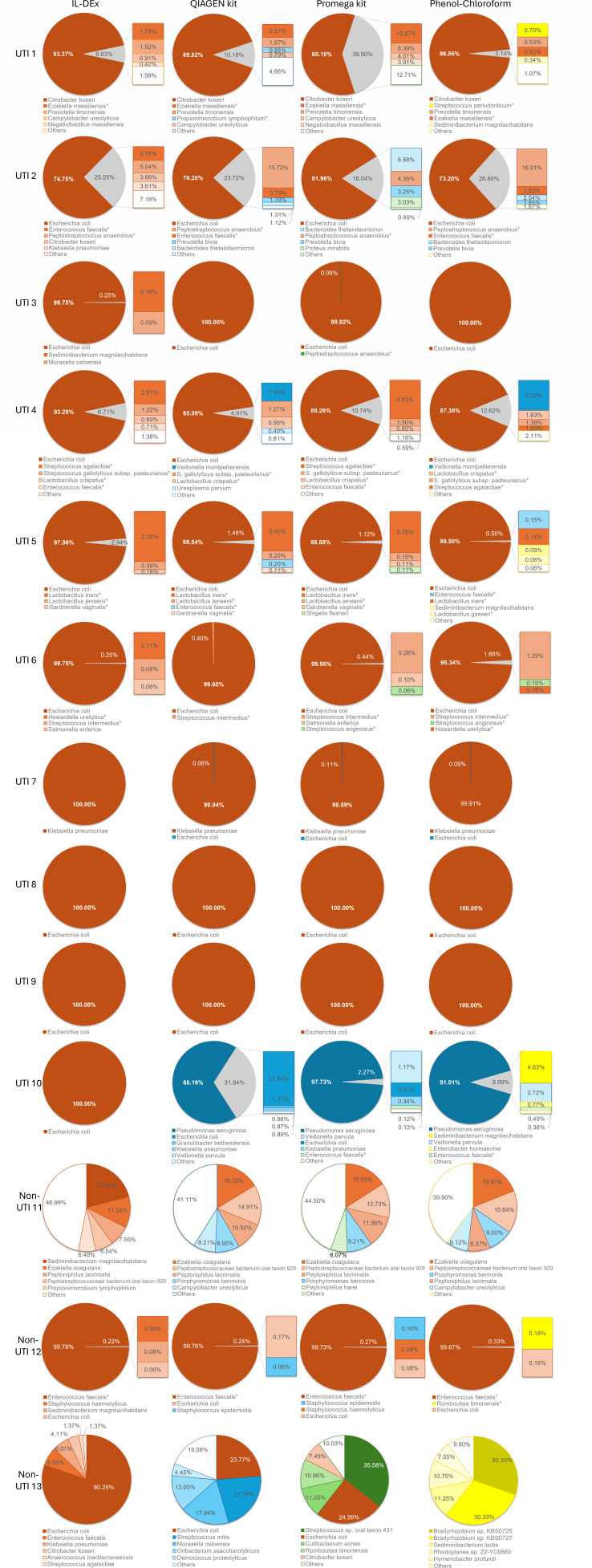
Full-length 16S rRNA gene sequencing results for the extracts from the ten UTI and three non-UTI samples obtained via IL-DEx, QIAGEN kit, Promega kit, and phenol-chloroform extraction. The five most abundant species are shown. The order of species in the legends under the corresponding charts corresponds to their relative abundance. Asterisks indicate gram-positive bacteria. Full raw data are available in Table S20.

Full-length 16S sequencing confirmed that all four extraction methods enabled detection of the dominant uropathogen identified by clinical culture (Fig. 5). In IL-DEx extracts, the dominant pathogen was detected at ≥75% relative abundance, except in UTI sample 10. In this sample, IL-DEx extract sequencing identified *E. coli*, whereas urine culture and sequencing of extracts from QIAGEN, Promega, and phenol-chloroform methods identified *P. aeruginosa*. However, *E. coli*-specific qPCR showed no detection in the IL-DEx extract (Fig. 4C), while a subsequent qPCR assay specific for *P. aeruginosa* confirmed its presence (data not shown), suggesting a possible sample mix-up during NGS library preparation. Aside from this discrepancy, the microbial community profiles were consistent across methods in UTI samples. Despite known challenges in lysing gram-positive bacteria, IL-DEx still recovered detectable levels of these taxa (e.g., *Enterococcus, Streptococcus, Lactobacillus, Staphylococcus, Gardnerella* sp.; Fig. 5). Sequencing of non-UTI extracts showed similar results across all extraction methods for non-UTI 11 and 12. In contrast, non-UTI 13 samples displayed substantial variability in taxonomic compositions depending on the extraction protocol used. Interestingly, this specimen contained the lowest bacterial biomass of all urine samples, as indicated by the low 16S rRNA gene copies (Fig. 4B) and the low total bacterial cell counts determined by epifluorescence microscopy (Fig. S8). When DNA quantities are low and close to the detection limit, stochastic amplification and reagent background can disproportionately influence sequencing outcomes, leading to apparent compositional differences and detection of sequences also present in extraction and negative control samples (47). These findings suggest that the observed discrepancies for non-UTI 13 likely reflect stochastic effects associated with extremely low microbial load rather than systematic differences between extraction methods.

## DISCUSSION

Rapid and reliable identification of uropathogens is essential for effective management of UTIs. With rising diagnostic demand and increasing antimicrobial resistance, molecular techniques, such as PCR, isothermal amplification, and deep sequencing, are gaining traction to expand current diagnostic options for UTIs (18, 48). However, their sensitivity, specificity, turnaround time, and cost are impacted by upstream DNA extraction steps. Extraction biases affecting microbial composition are well recognized, underscoring the importance of selecting suitable extraction methods for different sample types (49–51).

In this study, we demonstrated the feasibility of using ionic liquid chemistry for direct DNA extraction from urine, a specimen type that remains technically challenging for molecular diagnostics. Urine is characterized by variable microbial loads, abundant human host DNA, and inhibitory compounds (e.g., urea, salts, calcium oxalate crystals) that can impede bacterial DNA recovery (52–54). Although recent studies have optimized or compared different extraction approaches for urinary microbiota analyses, alternatives beyond traditional enzymatic, chemical, or mechanical disruption remain largely unexplored. Standardized and reproducible DNA extraction workflows are increasingly recognized as essential for minimizing methodological bias in urinary microbiome and molecular diagnostic research (28). Against this background, we evaluated IL-DEx, an ionic liquid-based method that integrates bacterial lysis and nucleic acid capture in a single-tube workflow using the hydrophilic ionic liquid [C_2_mim][OAc] and silica-coated magnetic beads. Compared to commercial kits and a phenol-chloroform protocol tested, IL-DEx offered several advantages: faster processing (~30 min), lower reagent costs (~2 € per prep), and elimination of hazardous solvents and specialized homogenization equipment (Table S24). While not entirely instrument-free - requiring centrifugation and heating for cell harvest and lysis - its simplicity and reagent accessibility support its application in both centralized laboratories and decentralized diagnostic settings.

Across spiking experiments and clinical specimens, IL-DEx produced DNA yields for gram-negative uropathogens comparable to the QIAamp DNA Mini Kit (equivalent to the DNeasy Blood and Tissue Kit, QIAGEN), which prior benchmarking studies identified as one of the most reliable options for urinary microbiota extraction (26, 55, 56). Matching QIAGEN’s performance for gram-negatives is clinically significant given that *E. coli*, *K. pneumoniae*, *P. mirabilis*, and *P. aeruginosa* cause the majority of community- and hospital-acquired UTIs (9, 57). In contrast, IL-DEx consistently yielded lower recovery from gram-positive bacteria (~1 log₁₀ lower), consistent with studies reporting that thick-walled organisms require more rigorous lysis (42, 57). This difference reflects a methodological extraction bias, demonstrated by qPCR on bacterial reference strains, clinical isolates, and spiked urine matrices, but does not indicate a categorical loss of detection. Both qPCR and long-read 16S sequencing confirmed that IL-DEx recovered sufficient DNA from gram-positive taxa for reliable detection, although typically at lower absolute and relative abundance. Sequencing of DNA extracts confirmed the presence of several gram-positive taxa at low abundance, in agreement with other tested extraction methods and alongside dominant uropathogens (Fig. 5). These included *Lactobacillus* spp. (UTI 4, 5), *Streptococcus* spp. (UTI 4, 6), *Enterococcus* spp. (UTI 2, 4), *Gardnerella vaginalis* (UTI 5), *Ezakiella massiliensis* (UTI 1), and *Peptostreptococcus anaerobius* (UTI 2). Importantly, these subdominant gram-positive taxa were consistently detected in IL-DEx extracts of clinical specimens whenever present in the corresponding QIAGEN, Promega, or phenol–chloroform preparations (Fig. 5). These results indicate that the extraction bias of IL-DEx toward gram-positive bacteria primarily affects quantitative representation (relative-abundance estimates) rather than detection sensitivity (loss of taxa or false negatives). While this compositional bias did not compromise the identification of dominant uropathogens in this study (dominated by gram-negative infections), we emphasize the need for larger, taxonomically diverse cohorts, including specimens dominated by gram-positive uropathogens and with polymicrobial infections, to comprehensively evaluate IL-DEx performance across cell-wall types and clinical contexts.

IL-DEx is therefore competitive with commercial silica spin-column methods for gram-negatives (QIAGEN), while offering a faster and chemically safe workflow. In comparison, Promega and phenol–chloroform protocols produced higher total DNA yields, but with more fragmented DNA and substantially more complex procedures, respectively. This trade-off echoes observations of earlier comparative studies, where yield advantages did not always translate into improved sequencing outcomes (26, 58). Thus, the combination of rapid processing, recovery of high molecular weight DNA, and simplified chemistry distinguishes IL-DEx from conventional extraction methods.

While the results of this study are encouraging, several limitations must be acknowledged. First, a clinical evaluation was performed on a small cohort of 13 urine specimens (ten culture-positive, three culture-negative), limiting conclusions about diagnostic sensitivity and specificity across diverse patient populations. Larger studies across patient groups and clinical presentations, with clearly defined downstream applications (e.g., multiplex PCR), are needed to establish clinical robustness. Second, although IL-DEx demonstrated compatibility with qPCR and nanopore-based 16S sequencing, its integration with other molecular platforms, such as isothermal amplification or shotgun metagenomic workflows, remains to be evaluated. Human-to-bacteria DNA ratios varied between samples (1:1 to 1,000:1), as determined by qPCR of 16S and human DNA content. Such variability has minimal impact on amplification-based assays (PCR, isothermal methods, targeted sequencing) but may impact microbial sequencing depth and sensitivity in untargeted (shotgun metagenomics) sequencing approaches (59). Third, fungal pathogens were not assessed in this study. Although less frequent than bacterial UTIs, *Candida* spp. are commonly implicated in catheter-associated UTIs and are associated with adverse outcomes in immunocompromised or critically ill patients (60). Given their chitin-rich cell walls, effective DNA extraction may require additional lysis strategies. Future work should explore IL-DEx’s utility in fungal diagnostics and polymicrobial infections.

In conclusion, IL-DEx offers a rapid, simple, and cost-effective approach to DNA extraction from urine specimens. Its compatibility with molecular platforms, ability to recover DNA from clinically relevant uropathogens, and potential for automation support its use in next-generation diagnostic workflows. As molecular testing continues to shift toward rapid, near-patient, and point-of-care applications, streamlined methods, such as IL-DEx, can help overcome pre-analytical barriers and extend access to advanced infectious disease diagnostics.

## Data Availability

Oxford Nanopore sequencing data have been deposited in the European Nucleotide Archive (ENA) under accession numbers ERS26910746–ERS26910803, associated with project PRJEB97800. Raw data from qPCR analyses, absorbance measurements, and sequencing can be found in the supplemental material (File SII).

## SUPPLEMENTAL MATERIAL

File SI spectrum.03191-25-s0001.docx Tables S21 to S24; Fig. S1 to S9.

File SII spectrum.03191-25-s0002.pdf Tables S1 to S20; data for figures and tables.

ASM does not own the copyrights to Supplemental Material that may be linked to, or accessed through, an article. The authors have granted ASM a non-exclusive, world-wide license to publish the Supplemental Material files. Please contact the corresponding author directly for reuse.
