# Supplementary material for: A rapid ionic liquid-based DNA extraction method for molecular diagnostics of urinary tract infections
Source: Microbiol Spectr. 2026 Feb 27;14(4):e03191-25. doi: 10.1128/spectrum.03191-25 (PMC13055219; doi:10.1128/spectrum.03191-25)
Supplement: File SI — Tables S21 to S24; Fig. S1 to S9. [file spectrum.03191-25-s0001.docx]

**Electronic Supplementary Material I**

**A rapid ionic liquid-based DNA extraction method for molecular diagnostics of urinary tract infections**

Johanna Kreuter^a,b^, Lena Piglmann^a,b^, Katarina Priselac^a,b^, Roland Martzy^c^, Michael Ante^c^, Dominik Walter^d,e^, Ildiko-Julia Pap^d,e^, Barbara Ströbele^d,e^, Andreas H. Farnleitner^b,f,g^, Georg H. Reischer^a,b^, Claudia Kolm^b,f^

^a^TU Wien, Institute of Chemical, Environmental and Bioscience Engineering, Research Unit Molecular Diagnostics (IFA-Tulln) Tulln, Austria

^b^ ICC Interuniversity Cooperation Centre Water & Health, Vienna, Austria ([www.waterandhealth.at](http://www.waterandhealth.at))

^c^ SAN Group GmbH, Herzogenburg, Austria

^d^ Institute of Hygiene and Microbiology, University Hospital St. Pölten, St. Pölten, Austria

^e^Karl Landsteiner University of Health Sciences, Krems, Austria

^f^ Karl Landsteiner University of Health Sciences, Department of Pharmacology, Physiology, and Microbiology, Division of Water Quality and Health, Krems, Austria

^g^TU Wien, Institute of Chemical, Environmental and Bioscience Engineering, Research Group for Microbiology and Molecular Diagnostics 166/5/3, Vienna, Austria

**Contents**

**Table S21** Detailed information on qPCR assays for quantification of DNA in extracts. Oligonucleotide sequences for qPCR

**Table S22** Oligonucleotide sequences for (q)PCRs.

**Table S23** Comparison of genome copy numbers with total bacterial cell counts for extracts from the QIAGEN kit.

***Fig S1*** *Spiking experiments with E. coli, E. faecalis and E. faecium in artificial urine to determine the detection limit of the analysis workflow.*

***Fig S2*** *Detected human DNA amount (in ng) in the extracts from 1 ml un-spiked urine.*

***Fig S3*** *Recovery of bacterial 16S rRNA gene copies from unspiked and E. coli–spiked urine and from E. coli spike control using IL-DEx and QIAGEN.*

***Fig S4*** *Log detected Enterococcus 23S rRNA gene copies in the extracts from 1 ml spiked and un-spiked urine and from the E. faecalis spike.*

***Fig S5*** *Recovery of Enterococcus faecalis 23S rRNA gene copies from unspiked and spiked urine and from E. faecalis spike control using IL-DEx and QIAGEN.*

***Fig S6*** *Photos of the clinical sample set received from St. Pölten University Hospital.*

***Fig S7*** *1% TBE agarose gel of selected extracts from clinical urines to investigate DNA integrity.*

***Fig S8*** *EFM images of clinical urine samples.*

***Fig S9*** *Content of human DNA (as ng) in the DNA extracts from clinical urines.*

***Table S24*** *Characteristics of performed extraction methods.*

**Table S21** Detailed information on qPCR assays for quantification of DNA in extracts. Due to E. coli DNA traces in the polymerase, low-level amplification was observed in NTCs in 16S-qPCR and E.coli-qPCR. Results were accepted if the NTCs contained less than 100 copies of the target per reaction.

| **qPCR Assay** | **Reaction Mix** | **Temperature protocol** |
| --- | --- | --- |
| **16S-qPCR** | 200 nM primer (Merck, Darmstadt, Germany)  7.5 µl KAPA™ SYBR® Fast qPCR Master Mix 2x (Peqlab, Erlangen, Germany)  2.5 µl DNA  H_2_O to 15 µl | 3 min at 95 °C  40 x (30 s at 95 °C, 30 s at 57 °C and 60 s at 72 °C)  2 min at 72 °C |
| ***E. coli*-qPCR** | 1 µM primer (Merck)  80 nM probe (Merck)  400 ng/µl BSA  7.5 µl Luna® Universal Probe qPCR Master Mix 2x (New England Biolabs, Frankfurt am Main, Germany)  2.5 µl DNA  H_2_O to 15 µl | 5 min at 95 °C  45 x (15 s at 95°C and 60 s at 60°C) |
| ***Enterococcus*-qPCR** | 1 µM primer (Merck)  80 nM probe (Merck)  7.5 µl KAPA™ PROBE FAST qPCR Master Mix 2x (Peqlab)  2.5 µl DNA  H_2_O to 15 µl | 5 min at 95 °C  40 x (15 s at 95°C and 60 s at 60°C) |
| **Human-qPCR** | 900 nM primer (Merck)  250 nM probe (Merck)  7.5 µl KAPA™ PROBE FAST qPCR Master Mix 2x (Peqlab)  2.5 µl DNA  H_2_O to 15 µl | 5 min at 95 °C  40 x (15 s at 95°C and 60 s at 60°C) |

**Table S22** Oligonucleotide sequences for (q)PCRs.

| **(q)PCR Assay** | **Oligonucleotide** | **Sequence 5’-3’** | **References** |
| --- | --- | --- | --- |
| **16S-qPCR** | 8F | AGAGTTTGATCCTGGCTCAG | [1] |
|  | 338R | TGCTGCCTCCCGTAGGAGT | [2] |
| ***E. coli*-qPCR** | EC23S857_F | GGTAGAGCACTGTTTTGGCA | [3] |
|  | EC23S857_R | TGTCTCCCGTGATAACTTTCTC |  |
|  | EC23S857_P | [6-FAM]-TCATCCCGACTTACCAACCCG-TAMRA |  |
| ***Enterococcus*-qPCR** | USEPA-Entc-F | GAGAAATTCCAAACGAACTTG | [4, 5] |
|  | USEPA-Entc-R | CAGTGCTCTACCTCCATCATT |  |
|  | USEPA-Entc-P | [6-FAM]-TGGTTCTCTCCGAAATAGCTTTAGGGCTA-TAMRA |  |
| **Human-qPCR** | Alu human DNA_fwd | CATGGTGAAACCCCGTCTCTA | [6] |
|  | Alu human DNA_rev | GCCTCAGCCTCCCGAGTAG |  |
|  | Alu human DNA_probe | [6-FAM]-ATTAGCCGGGCGTGGTGGCG-TAMRA |  |
| **Library preparation**  **16S Amplification** | 27F  *modified with wobble positions* | AGRGTTYGATYMTGGCTCAG |  |
|  | 1492R  *modified with wobble positions* | RGYTACCTTGTTACGACTT |  |

**Table S23** Comparison of genome copy numbers with total bacterial cell counts for extracts from the QIAGEN kit. Genome copy numbers were calculated by dividing the detected 16S rRNA gene copy number by the mean number of 16S rRNA operons per genome (E. coli: 7 16S gene copies per genome, P. aeruginosa: 4, K. pneumoniae: 8, P. mirabilis: 7, E. faecalis: 4, E. faecium: 6, and S. saprophyticus: 6) [44]. Data are reported as mean ± standard deviation (SD) from three biological replicates.

| **Strain** | **Method** | **Genome copy numbers**  **[Mean ± SD]** | **Total bacterial cell counts**  **[Mean ± SD]** |
| --- | --- | --- | --- |
| ***E. coli*** | QIAGEN | 1.13 x 10^8^ ± 7.22 x 10^6^ | 2.36 x 10^7^ ± 1.20 x 10^7^ |
| ***P. aeruginosa*** | QIAGEN | 8.86 x 10^7^ ± 2.32 x 10^7^ | 1.56 x 10^8^ ± 1.55 x 10^7^ |
| ***K. pneumoniae*** | QIAGEN | 1.01 x 10^8^ ± 3.68 x 10^6^ | 9.76 x 10^7^ ± 3.98 x 10^6^ |
| ***P. mirabilis*** | QIAGEN | 2.68 x 10^7^ ± 5.09 x 10^6^ | 7.80 x 10^7^ ± 7.14 x 10^6^ |
| ***E. faecalis*** | QIAGEN | 1.81 x 10^8^ ± 9.42 x 10^7^ | 8.37 x 10^7^ ± 1.18 x 10^7^ |
| ***E. faecium*** | QIAGEN | 1.47 x 10^8^ ± 2.27 x 10^7^ | 1.27 x 10^8^ ± 1.05 x 10^7^ |
| ***S. saprophyticus*** | QIAGEN | 6.56 x 10^7^ ± 2.40 x 10^7^ | 7.01 x 10^7^ ± 4.14 x 10^6^ |


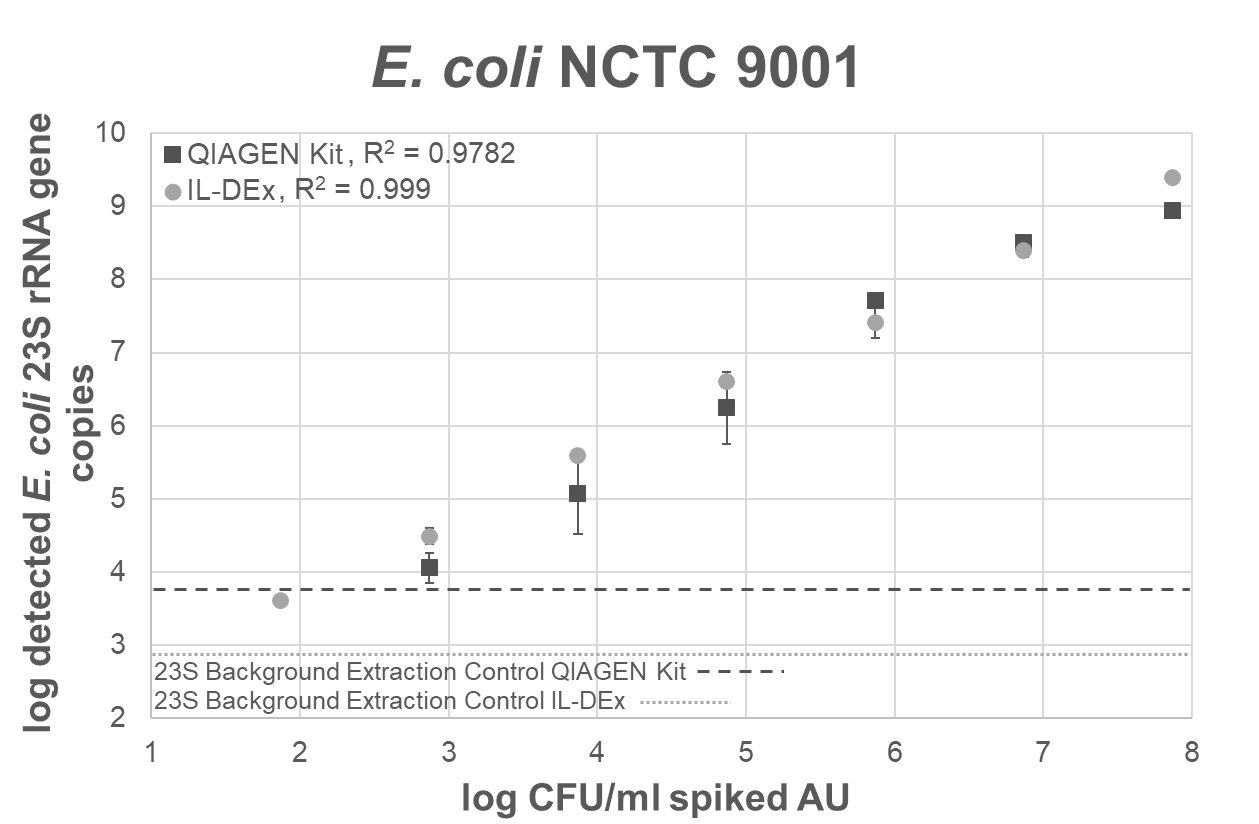

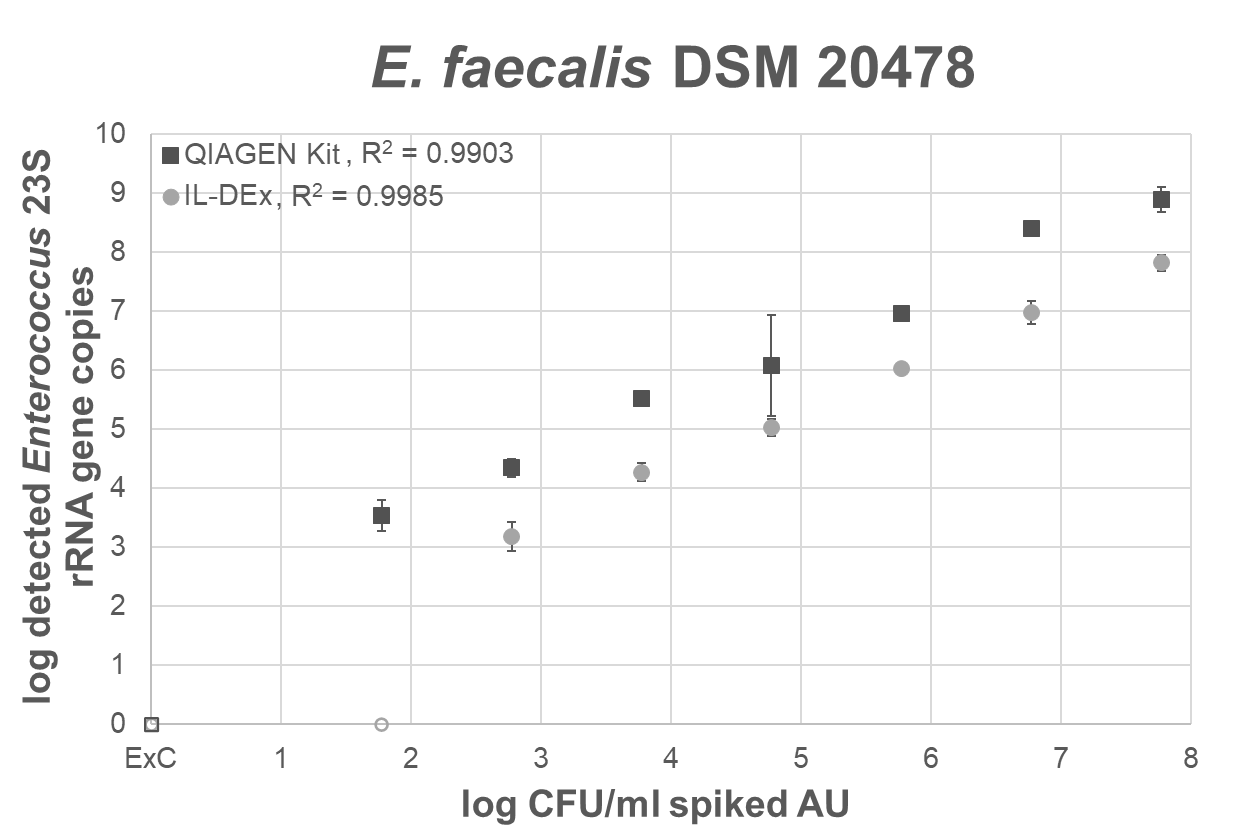

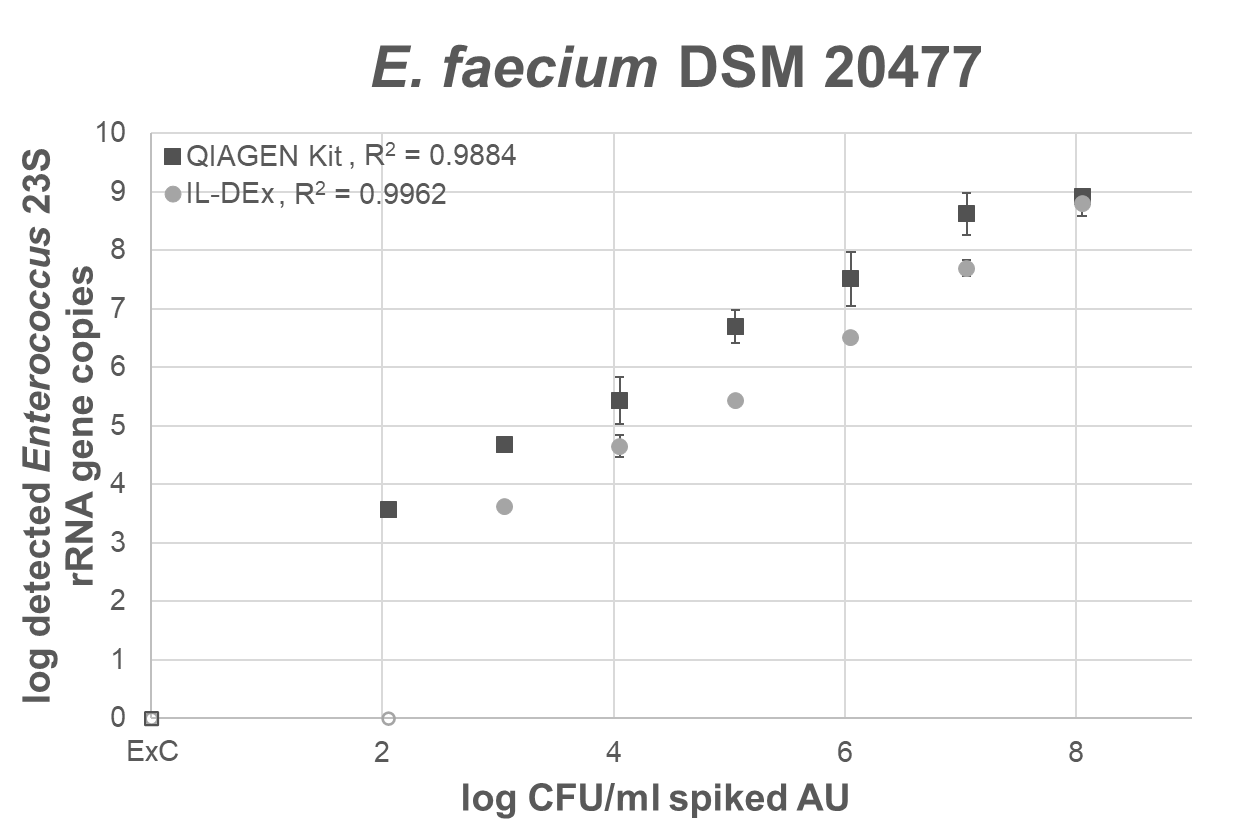


***Fig S1*** *Spiking experiments in artificial urine to determine the detection limit of the analysis workflow. E. coli, E. faecalis and E. faecium samples were analyzed with a specific E. coli- and Enterococcus-qPCR assay. Values shown are mean values from three biological replicates, whiskers indicate the standard deviation. For E. coli, the dotted lines represent the E. coli 23S background of the extraction controls (un-spiked AU). A three-sigma limit was chosen to exclude all datapoints that lie within three standard deviations from the mean of these controls.* *For E. faecalis and E. faecium, no Enterococci DNA was detected in the extraction controls (ExC) of both methods.* *For raw data see Supplementary II Table S9-S11.*

*
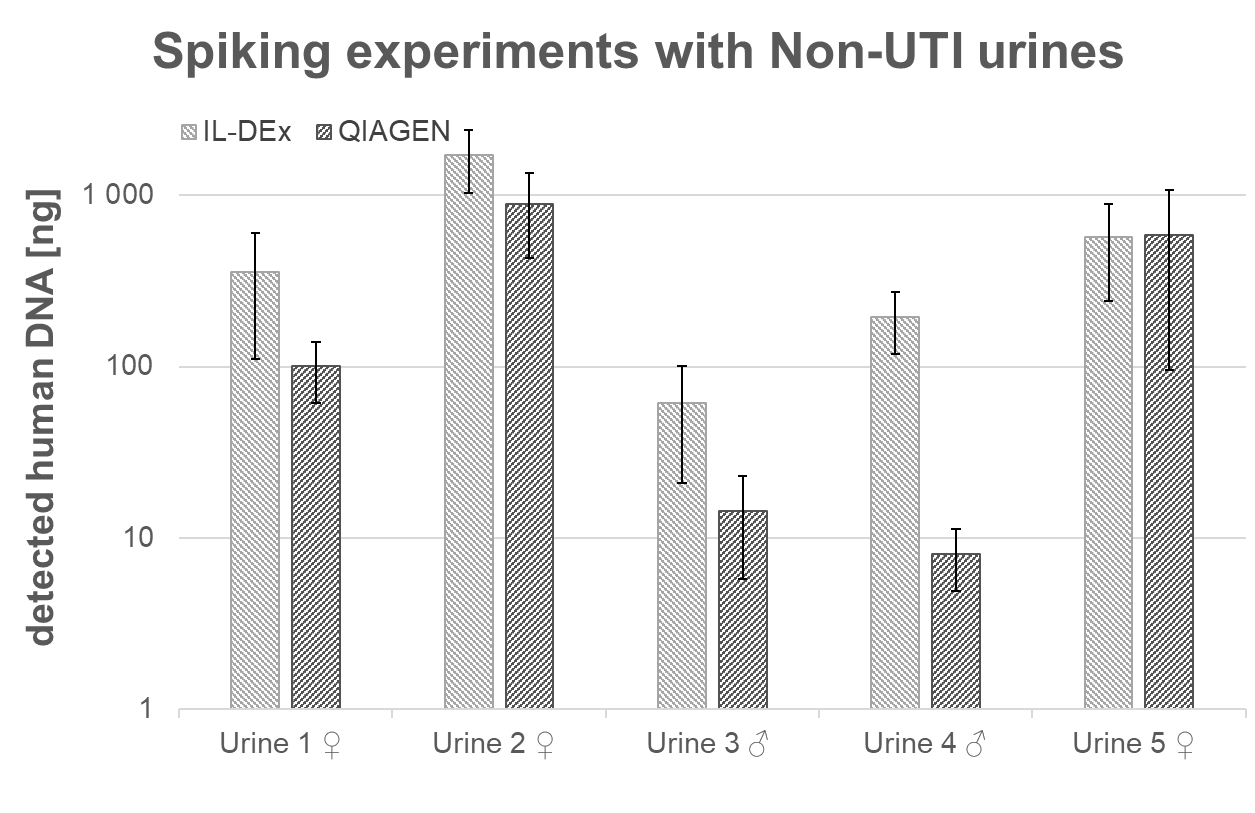
*

***Fig S2*** *Detected human DNA amount (in ng) in the extracts from 1 ml un-spiked urine. Extracts from IL-DEx (light grey) and QIAGEN kit (dark grey). Values shown are mean values from three biological replicates, whiskers indicate the standard deviation. Paired t‑tests on human DNA yield across the five urine samples showed no statistically significant difference between IL-DEx and QIAGEN (p = 0.16). For raw data see Supplementary II Table S13.*


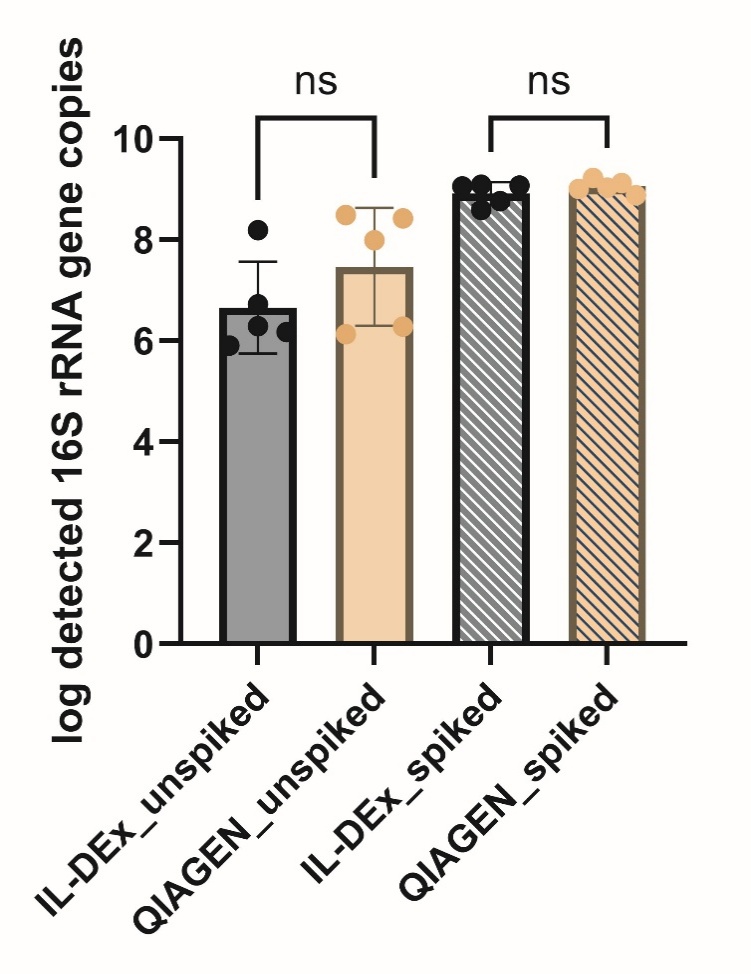

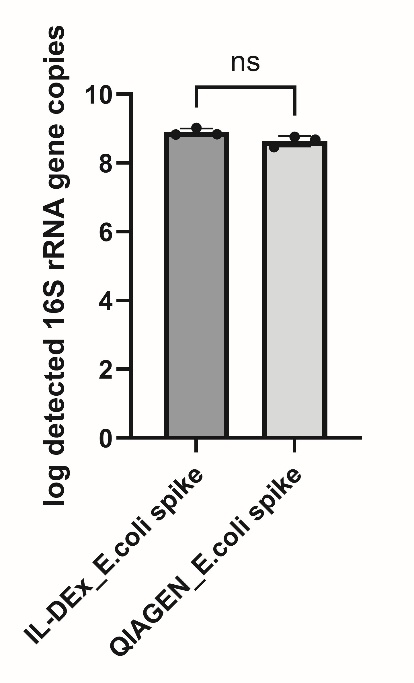


***Fig S3*** *Recovery of bacterial 16S rRNA gene copies from unspiked and E. coli–spiked urine (left panel) and from E. coli spike control (right panel) using IL-DEx and QIAGEN.*

*Left panel: Log_10_-transformed 16S rRNA gene copy numbers measured in urine from five donors extracted with IL-DEx and QIAGEN under two conditions: unspiked urine and urine spiked with E. coli. Each data point represents an individual donor, with paired values for IL-DEx and QIAGEN within each condition. Bars indicate mean ± SD. Paired t-tests comparing IL-DEx and QIAGEN within unspiked and spiked urine showed no statistically significant differences in 16S rRNA gene copy numbers (both p ~ 0.18), indicating broadly comparable bacterial DNA recovery between the two extraction methods across donor samples.*

*Right panel: Log_10_-transformed 16S rRNA gene copy numbers obtained from a homogeneous E. coli spike suspension extracted in triplicate with each method. Data points represent individual replicate extractions; bars indicate mean ± SD. A paired t-test comparing IL-DEx and QIAGEN did not detect a statistically significant difference in recovered E. coli 16S copies (p = 0.104), indicating broadly comparable DNA recovery from the spike-only control.*

**
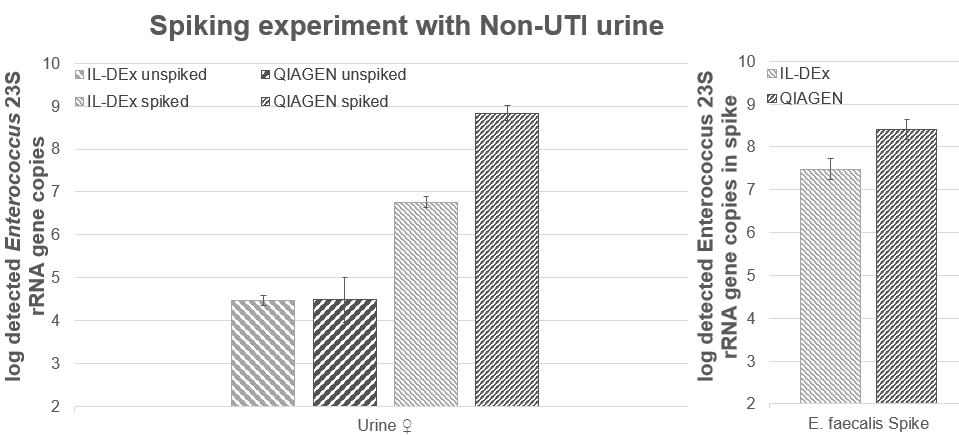
**

***Fig S4*** *Log detected Enterococcus 23S rRNA gene copies in the extracts from 1 ml unspiked and E.faecalis-spiked urine (left panel) and from the E. faecalis cell suspension used for spiking (right panel). Extracts from IL-DEx (light grey) and QIAGEN kit (dark grey). Urine was spiked to approx. 10^7^CFUs/ml. Values shown are mean values from three biological replicates, whiskers indicate the standard deviation. In unspiked urine, both extraction methods yielded similar background 23S rRNA gene copy numbers (paired t-test, not significant, p > 0.05) (Fig. S5, left panel). In E. faecalis-spiked urine, QIAGEN recovered significantly higher 23S rRNA gene copies than IL-DEx (mean difference = 2.07 log₁₀ units, p < 0.0001), indicating markedly greater apparent recovery of E. faecalis DNA by the QIAGEN kit (Fig. S5, left panel). Comparable results were obtained for the E. faecalis spike-only control (right panel). QIAGEN recovered significantly higher E. faecalis 16S copy numbers than IL-DEx (paired t-test, p = 0.0054), with a mean difference of 0.92 log10 units, corresponding to an approximately 8-fold higher apparent recovery (Fig. S5, right panel). For raw data see Supplementary II Table S14.*


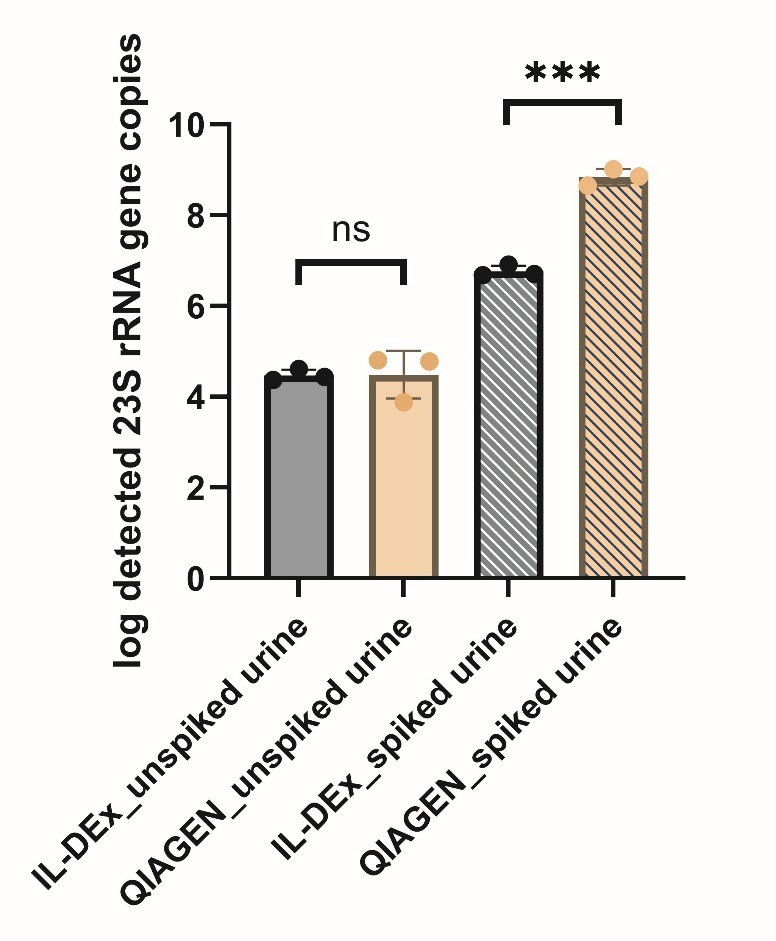

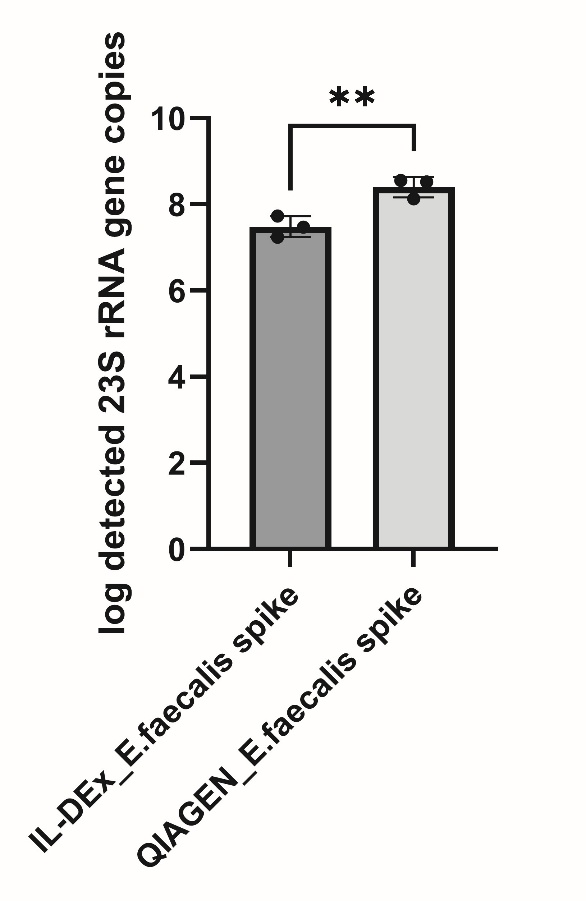


***Fig S5*** *Recovery of Enterococcus faecalis 23S rRNA gene copies from unspiked and spiked urine (left panel) and from E. faecalis spike control (right panel) using IL-DEx and QIAGEN.*

*Left panel: Log_10_-transformed 23S rRNA gene copy numbers measured from a single female donor urine sample either unspiked or spiked with E. faecalis, then extracted in triplicate with IL-DEx and QIAGEN. Each point represents one replicate extraction; bars indicate mean ± SD. Within each condition, paired t-tests were used to compare IL-DEx and QIAGEN extractions. No statistically significant difference was observed in unspiked urine (ns, p > 0.05). In E. faecalis–spiked urine, QIAGEN recovered substantially higher 23S rRNA copy numbers than IL-DEx (paired t-test, mean difference = 2.07 log₁₀ units, p = 0.001), Right panel: Log_10_-transformed 23S rRNA gene copy numbers measured from a homogeneous E. faecalis spike-in suspension extracted in triplicate with IL-DEx and QIAGEN. Data points represent individual replicate extractions; bars indicate mean ± SD. QIAGEN recovered significantly higher E. faecalis 16S copy numbers than IL-DEx (paired t-test, p = 0.0054), with a mean difference of 0.92 log10 units, corresponding to an approximately 8-fold higher apparent recovery.*

*
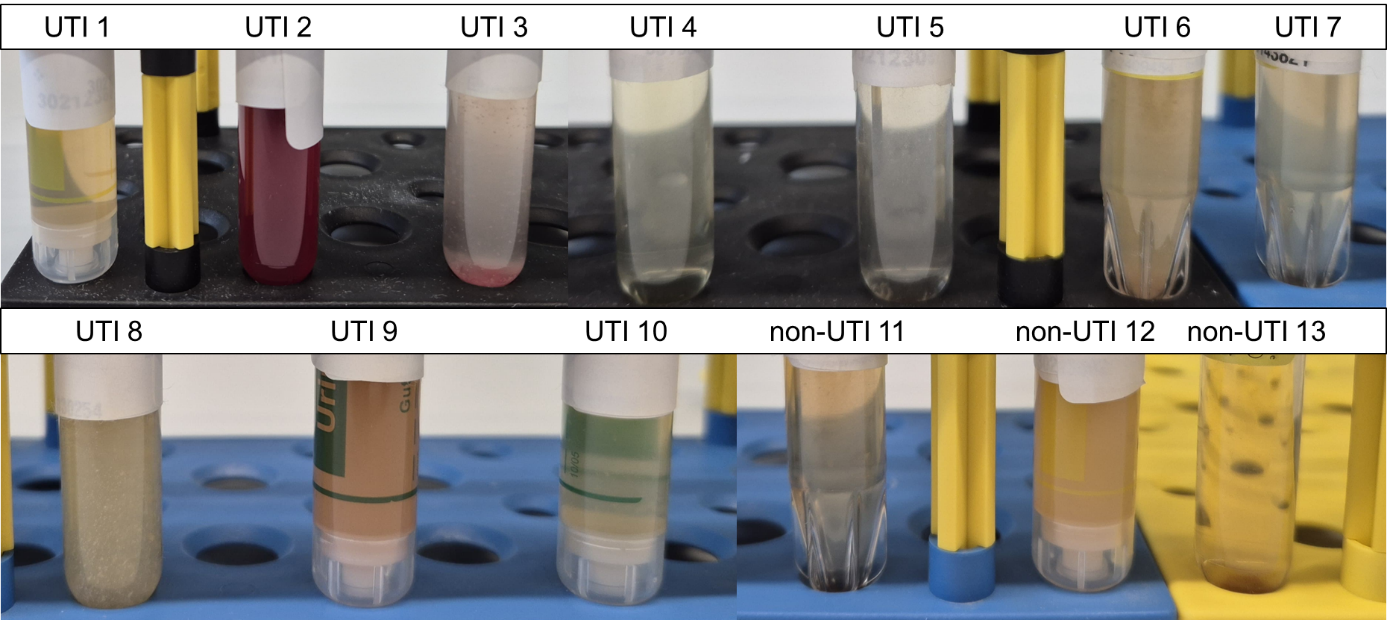
*

***Fig S6*** *Photos of the clinical sample set received from St. Pölten University Hospital. Based on the clinical report, a total of 10 suspected UTI and 3 non-UTI urine samples were received.*


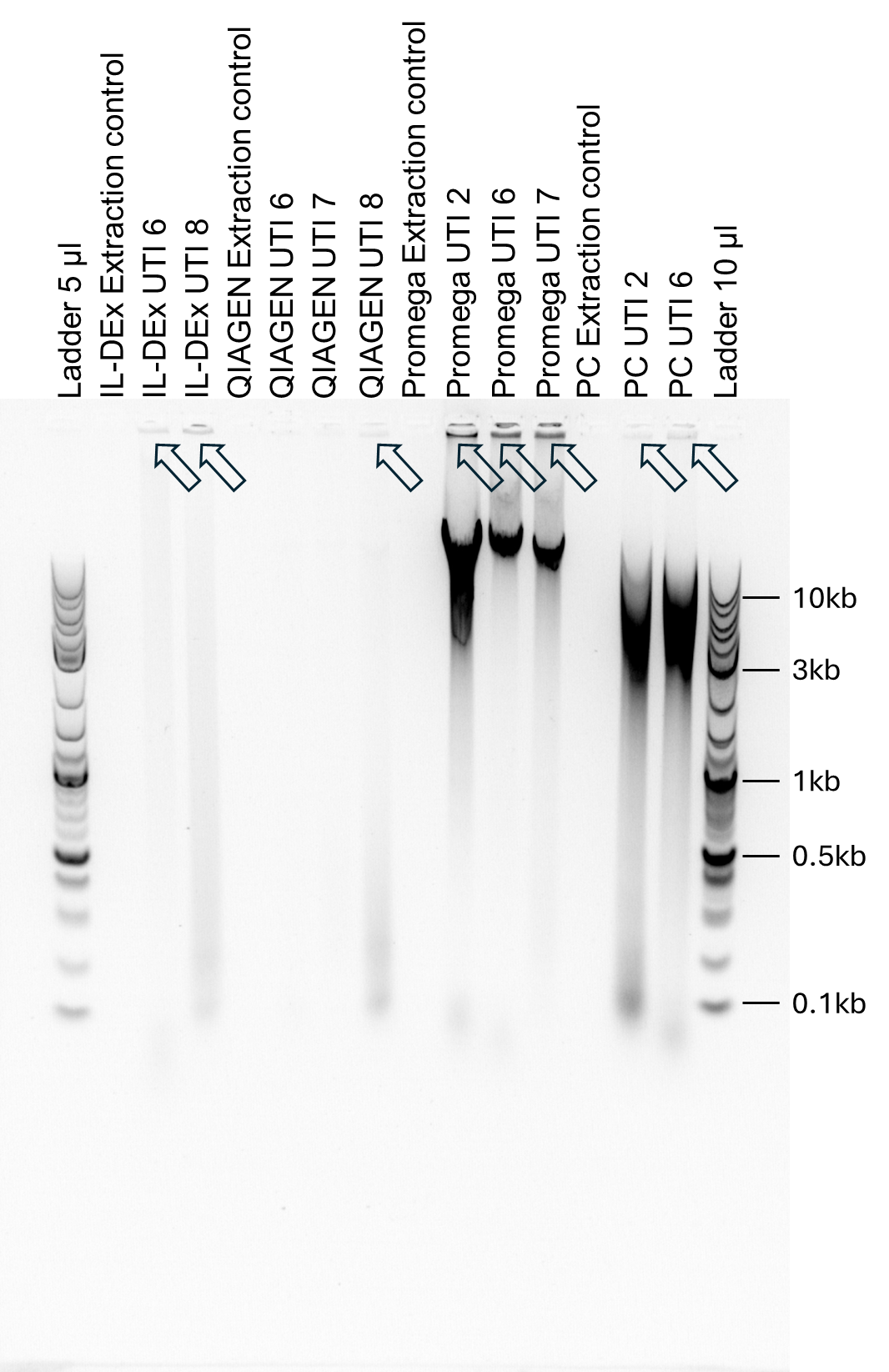


***Fig S7*** *1% TBE agarose gel of selected extracts from clinical urines to investigate DNA integrity (PC = Phenol-chloroform extraction). 10 µl of extract and 5 or 10 µl of 1 kB Plus DNA Ladder (New England Biolabs, Frankfurt am Main, Germany) were loaded. Gel was run at 90 V for 60 min. DNA was visualized with SYBR Gold. Arrows indicate integer genomic DNA. Black smears indicate fragmented DNA.*


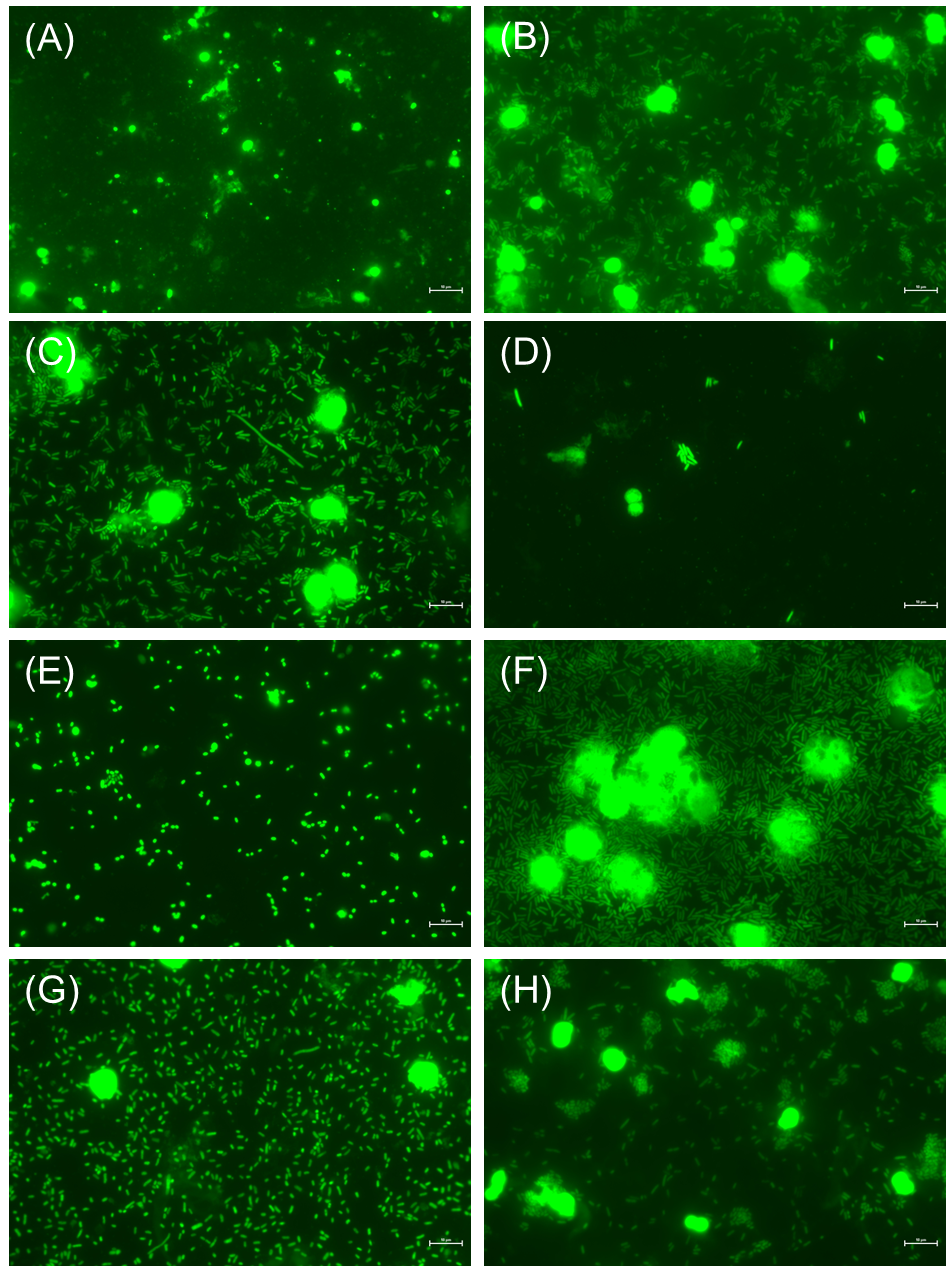


***Fig S8*** *EFM images of clinical urine samples. Urines were 1:10 diluted. filtered on a polycarbonate 0.2 µm filter, stained with SYBR gold, and imaged under an epifluorescence microscope (scale bar: 10 µm; 1 000x magnification). (A) UTI 1, 1 ms exposure time. (B) UTI 2, 30ms exposure time. (C) UTI 3, 30ms exposure time. (D) UTI 4, 30ms exposure time. (E) UTI 5, 30ms exposure time. (F) UTI 6, 30ms exposure time. (G) UTI 7, 30ms exposure time. (H) UTI 8, 30ms exposure time.*


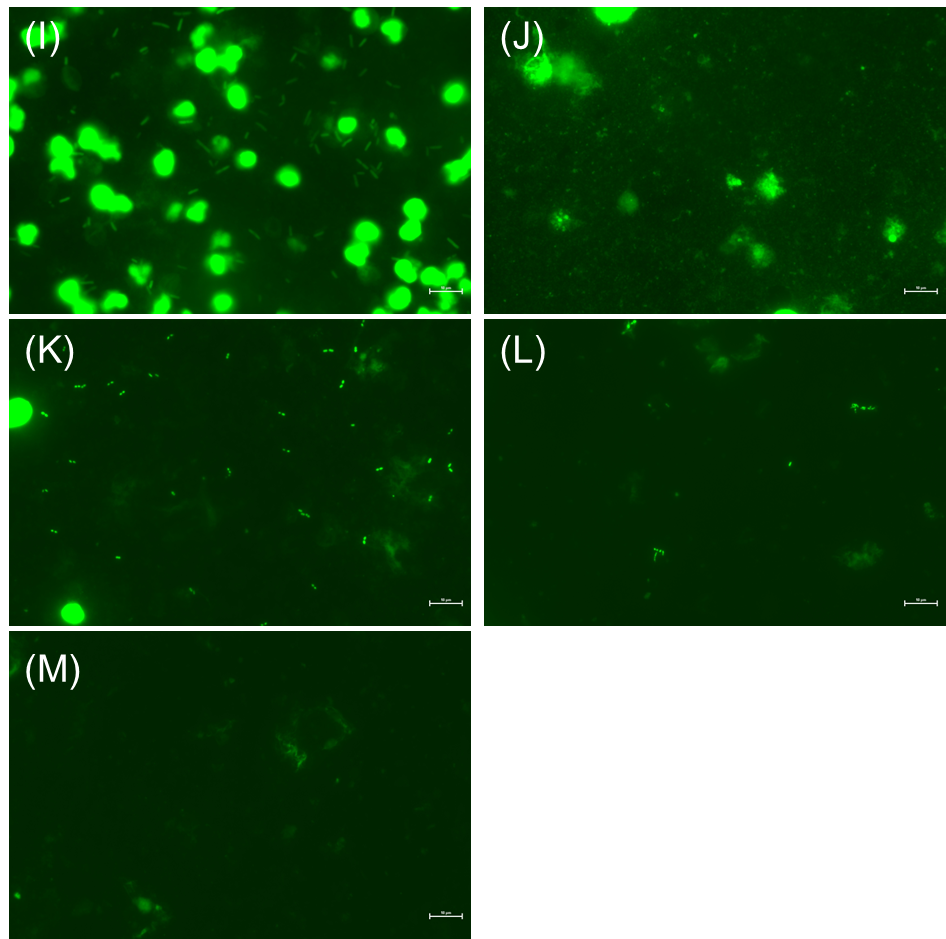


***Fig S8 Continued*** *EFM images of clinical urine samples. Urines were 1:10 diluted. filtered on a polycarbonate 0.2 µm filter, stained with SYBR gold, and imaged under an epifluorescence microscope (scale bar: 10 µm; 1 000x magnification). (I) UTI 9, 30ms exposure time. (J) UTI 10, 30ms exposure time. (K) Non-UTI 11, 30ms exposure time. (L) Non-UTI 12, 30ms exposure time. (M) Non-UTI 13, 30ms exposure time.*


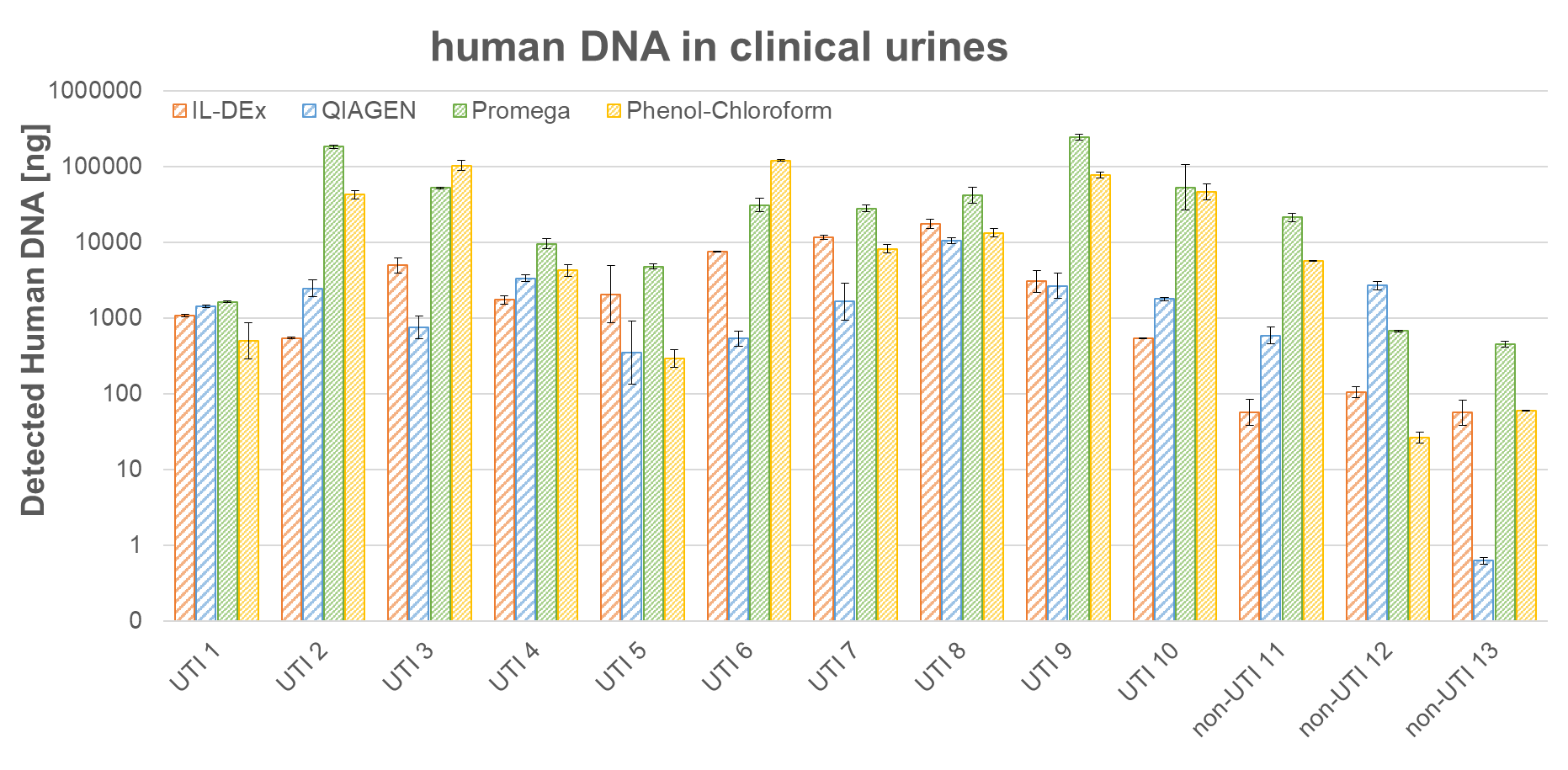


***Fig S9*** *Content of human DNA (as ng) in the DNA extracts from clinical urines obtained via IL-DEx (orange), QIAGEN kit (blue), Promega kit (green) and phenol-chloroform extraction (yellow). Values shown are mean values from two biological replicates, whiskers indicate the two measured values. For each replicate 1 ml of urine was used. For raw data see Supplementary II Table S19.*

**Table S24** Characteristics of performed extraction methods. L = Lysozyme, PK = Proteinase K, IL = Ionic liquid.

| ***Method*** | ***Lysis*** | | | ***Clean-up*** | ***Materials*** | ***Cost & Time*** |
| --- | --- | --- | --- | --- | --- | --- |
|  | ***Mechanical*** | ***Enzymatic*** | ***Chemical*** |  |  |  |
| **IL-DEx** | × | × | ✓ | MB | IL  10mM Tris buffer  Mag. beads  70% EtOH  TE buffer  Magnetic rack  Heating and Shaking block  Centrifuge | ≈2€  20min |
| **QIAGEN kit** | × | L, PK | ✓ | Silica column | L solution  PK solution  96% ethanol  Buffer 1  Buffer 2  Buffer 3  Buffer 4  Buffer 5  Heating and Shaking block  Centrifuge | 4.94€  80min |
| **Promega kit** | × | PK | ✓ | MB | TE buffer  PK solution  Mag beads  Buffer 1  Buffer 2  Buffer 3  Buffer 4  Heating and Shaking block  Magnetic rack  Centrifuge | 2.47€  40min |
| **Phenol-Chloroform** | Bead beating | × | ✓ | Precipitation | Glass beads  Phenol  Chloroform  Isoamyl alc.  CTAB buffer  Isopropanol  70% EtOH  10mM Tris buffer  Homogenizer  Centrifuge | ≈2-3€  120min |

**References:**

1. Edwards, U., et al., *Isolation and direct complete nucleotide determination of entire genes. Characterization of a gene coding for 16S ribosomal RNA.* Nucleic Acids Res, 1989. **17**(19): p. 7843-53.

2. Etchebehere, C. and J. Tiedje, *Presence of two different active nirS nitrite reductase genes in a denitrifying Thauera sp. from a high-nitrate-removal-rate reactor.* Appl Environ Microbiol, 2005. **71**(9): p. 5642-5.

3. Chern, E.C., et al., *Comparison of quantitative PCR assays for Escherichia coli targeting ribosomal RNA and single copy genes.* Letters in applied microbiology, 2011. **52**: p. 298-306.

4. Office of Water, U.S.E.P.A., *Method 1611: Enterococci in Water by TaqMan quantitative polymerase chain reaction (qPCR) assay. Technical Report EPA-821R-12-008*. 2012: Washington, DC.

5. Office of Water, U.S.E.P.A., *Method 1609: Enterococci in Water by TaqMan Quantitative Polymerase Chain Reaction (qPCR) with Internal Amplification Control (IAC) Assay. Technical Report EPA-820-R-13-005*. 2013: Washington, DC.

6. McBride, C., D. Gaupp, and D.G. Phinney, *Quantifying levels of transplanted murine and human mesenchymal stem cells in vivo by real-time PCR.* Cytotherapy, 2003. **5**(1): p. 7-18.
