# Supplementary material for: A rapid ionic liquid-based DNA extraction method for molecular diagnostics of urinary tract infections
Source: Microbiol Spectr. 2026 Feb 27;14(4):e03191-25. doi: 10.1128/spectrum.03191-25 (PMC13055219; doi:10.1128/spectrum.03191-25)
Supplement: File SII — Tables S1 to S20; data for figures and tables. [file spectrum.03191-25-s0002.pdf]

Electronic Supplementary Material I I

A rapid ionic liquid-based DNA extraction method for molecular diagnostics of urinary tract infections

Johanna Kreuter[a,b], Lena Piglmann[a,b], Katarina Priselac[a,b], Roland Martzy[c], Michael Ante[c], Dominik Walter[d,e], Ildiko-Julia Pap[d,e], Barbara Ströbele[d,e], Andreas H. Farnleitner[b,f,g], Georg H. Reischer[a,b], Claudia Kolm[b,f]

[a] TU Wien, Institute of Chemical, Environmental and Bioscience Engineering, Research Unit Molecular Diagnostics (IFA-Tulln) Tulln, Austria

b ICC Interuniversity Cooperation Centre Water & Health, Vienna, Austria (www.waterandhealth.at)

[c] SAN Group GmbH, Herzogenburg, Austria

[d] Institute of Hygiene and Microbiology, University Hospital St. Pölten, St. Pölten, Austria

[e] Karl Landsteiner University of Health Sciences, Krems, Austria

[f] Karl Landsteiner University of Health Sciences, Department of Pharmacology, Physiology, and Microbiology, Division of Water Quality and Health, Krems, Austria

[g] TU Wien, Institute of Chemical, Environmental and Bioscience Engineering, Research Group for Microbiology and Molecular Diagnostics 166/5/3, Vienna, Austria

Contents

Table S1 *Data for Table 1. Extraction experiments with bacterial reference strains and clinical isolates.*
Table S2 *Data for Fig. 2. Spiking Experiments with E. coli NCTC 9001 in artificial urine.*
Table S3 *Data for Fig. 2. Spiking Experiments with P. aeruginosa NCTC 10662 in artificial urine.*
Table S4 *Data for Fig. 2. Spiking Experiments with K. pneumoniae DSM 30104 in artificial urine.*
Table S5 *Data for Fig. 2. Spiking Experiments with P. mirabilis DSM 4479 in artificial urine.*
Table S6 *Data for Fig. 2. Spiking Experiments with E. faecalis DSM 20478 in artificial urine.*
Table S7: *Data for Fig. 2. Spiking Experiments with E. faecium DSM 20477 in artificial urine.*
Table S8 *Data for Fig. 2. Spiking Experiments with S. saprophyticus DSM 20229 in artificial urine.*
Table S9 *Data for Fig. S1. Spiking Experiments with E. coli NCTC 9001 in artificial urine.*
Table S10 *Data for Fig. S1. Spiking Experiments with E. faecalis DSM 20478 in artificial urine.*
Table S11 *Data for Fig. S1. Spiking Experiments with E. faecium DSM 20477 in artificial urine.*
Table S12 *Data for Fig. 3 and Fig. S3. Spiking Experiments with E. coli NCTC 9001 in five non-UTI urines.*
Table S13 *Data for Fig. S2. Human DNA in five non-UTI urines from the E. coli Spiking Experiments.*
Table S14 *Data for Fig. S4 and S5. Spiking Experiments with E. faecalis DSM 20487 in a non-UTI urine.*
Table S15 *Data for Fig. 4A. Total nucleic acid content of extracts from clincial urines.*
Table S16 *Data for Fig. 4A. Total DNA content of extracts from clinical urines.*
Table S17 *Data for Fig. 4B. 16S rRNA gene content of extracts from clinical urines.*
Table S18 *Data for Fig. 4C. E. coli 23S rRNA gene content of extracts from clinical urines.*
Table S19 *Data for Fig. S9. Human DNA content of extracts from clinical urines.*
Table S20 *Data for Fig. 5. 16S rRNA gene sequencing results for extracts from clinical urines.*

Table S1: Data for Table 1. Extraction experiments with bacterial reference strains and clinical isolates. Extract from 10 µl pure bacterial culture. Measured with 16S-qPCR.

Organism	Strain	OD_{600}	Method	16S rRNA gene copies in extract	Mean [copies in extract]	SD	RSD [%]	Extraction Efficiency [%]	16S rRNA gene logcopies in extract	Mean [logcopies in extract]	SD
E. faecalis				9.40E+06					6.97		
	DSM20478	1.1	IL-DEx	1.02E+07	**9.31E+06**	8.61E+05	9%	0.76%	7.01	6.97	0.04
				8.45E+06					6.93		
				1.47E+09					9.17		
	DSM20478		QIAGEN Kit	8.33E+08	**1.23E+09**	3.81E+08	31%	100%	8.92	9.09	0.15
				1.52E+09					9.18		
				2.82E+07					7.45		
	IHM 5490	0.7	IL-DEx	5.45E+07	**3.49E+07**	1.54E+07	44%	8%	7.74	7.54	0.17
				2.76E+07					7.44		
				3.55E+08					8.55		
	IHM 5490		QIAGEN Kit	3.96E+08	**4.18E+08**	8.66E+07	21%	100%	8.60	8.62	0.09
				5.21E+08					8.72		
E. faecium				2.77E+07					7.44		
	DSM20477	1.3	IL-DEx	2.42E+07	**2.52E+07**	2.16E+06	9%	2.94%	7.38	7.40	0.04
				2.37E+07					7.38		
				6.55E+08					8.82		
	DSM20477		QIAGEN Kit	1.24E+09	**8.56E+08**	3.07E+08	36%	100%	9.09	8.93	0.14
				7.75E+08					8.89		
				9.31E+06					6.97		
	IHM 5458	0.8	IL-DEx	6.99E+06	**8.74E+06**	1.68E+06	19%	2%	6.84	6.94	0.09
				1.03E+07					7.01		
				4.34E+08					8.64		
	IHM 5458		QIAGEN Kit	4.32E+08	**4.22E+08**	1.84E+07	4%	100%	8.64	8.63	0.02
				4.01E+08					8.60		
S. saprophyticus				3.93E+06					6.59		
	DSM20229	1.1	IL-DEx	3.89E+06	**3.89E+06**	4.00E+04	1%	0.73%	6.59	6.59	0.00
				3.85E+06					6.59		
				5.68E+08					8.75		
	DSM20229		QIAGEN Kit	5.07E+08	**5.31E+08**	3.20E+07	6%	100%	8.70	8.73	0.03
				5.21E+08					8.72		
				7.26E+06					6.86		
	IHM 5493	0.8	IL-DEx	8.15E+06	**7.53E+06**	5.28E+05	7%	3%	6.91	6.88	0.03
				7.21E+06					6.86		
				2.54E+08					8.41		
	IHM 5493		QIAGEN Kit	2.39E+08	**2.21E+08**	4.01E+07	18%	100%	8.38	8.35	0.08
				1.79E+08					8.25		
E. coli				1.36E+09					9.13		
	NCTC9001	1.1	IL-DEx	9.40E+08	**1.12E+09**	2.12E+08	19%	84.99%	8.97	9.05	0.08
				1.11E+09					9.04		
				1.42E+09					9.15		
	NCTC9001		QIAGEN Kit	1.29E+09	**1.32E+09**	8.45E+07	6%	100%	9.11	9.12	0.03
				1.26E+09					9.10		
				4.15E+08					8.62		
	IHM 5531	0.8	IL-DEx	2.77E+08	**3.37E+08**	6.93E+07	21%	85%	8.44	8.53	0.09
				3.34E+08					8.52		
				4.38E+08					8.64		
	IHM 5531		QIAGEN Kit	3.07E+08	**3.95E+08**	8.23E+07	21%	100%	8.49	8.60	0.10
				4.58E+08					8.66		
P. aeruginosa				8.73E+08					8.94		
	NCTC10662	1	IL-DEx	6.61E+08	**7.04E+08**	1.42E+08	20%	102.36%	8.82	8.85	0.08
				6.05E+08					8.78		
				6.28E+08					8.80		
	NCTC10662		QIAGEN Kit	6.84E+08	**6.88E+08**	6.56E+07	10%	100%	8.83	8.84	0.04
				7.58E+08					8.88		
				4.16E+08					8.62		
	IHM 5716	0.7	IL-DEx	4.44E+08	**3.73E+08**	8.69E+07	23%	62%	8.65	8.57	0.11
				2.81E+08					8.45		
				6.15E+08					8.79		
	IHM 5716		QIAGEN Kit	6.96E+08	**6.03E+08**	9.27E+07	15%	100%	8.84	8.78	0.07
				5.11E+08					8.71		
K. pneumoniae				7.70E+08					8.89		
	DSM30104	1	IL-DEx	8.17E+08	**8.49E+08**	1.08E+08	13%	55.53%	8.91	8.93	0.05
				9.75E+08					8.99		
				1.71E+09					9.23		
	DSM30104		QIAGEN Kit	1.37E+09	**1.53E+09**	1.71E+08	11%	100%	9.14	9.18	0.05
				1.52E+09					9.18		
				2.50E+08					8.40		
	IHM 5571	0.8	IL-DEx	2.51E+08	**2.17E+08**	5.16E+07	24%	47%	8.40	8.34	0.11
				1.61E+08					8.21		
				4.61E+08					8.66		
	IHM 5571		QIAGEN Kit	4.79E+08	**4.62E+08**	1.67E+07	4%	100%	8.68	8.66	0.02
				4.46E+08					8.65		
P. mirabilis				1.32E+09					9.12		
	DSM4479	1.1	IL-DEx	1.48E+09	**1.52E+09**	2.53E+08	17%	89.85%	9.17	9.18	0.07
				1.81E+09					9.26		
				1.66E+09					9.22		
	DSM4479		QIAGEN Kit	1.44E+09	**1.69E+09**	3.02E+08	18%	100%	9.16	9.23	0.08
				2.04E+09					9.31		
				5.11E+08					8.71		
	IHM 5533	0.7	IL-DEx	4.19E+08	**4.49E+08**	5.24E+07	12%	78%	8.62	8.65	0.05
				4.22E+08					8.63		
				6.50E+08					8.81		
	IHM 5533		QIAGEN Kit	5.90E+08	**5.74E+08**	7.87E+07	14%	100%	8.77	8.76	0.06
				4.94E+08					8.69		

Table S2: Data for Fig. 2. Spiking Experiments with E. coli NCTC 9001 in artificial urine. Extract from 1 ml of spiked artificial urine. Measured with 16S-qPCR. The 16S background was calculated from the mean of the extraction control + 3x standard deviation and is the limit above which values are true positives with a probability of 95%. Values of the dilutions series that fall under the limit (grey) were not included in the diagram.

CFU/ml	log CFU/ml	Method	16S rRNA gene content [copies in extract]	Mean [copies in extract]	SD [copies in extract]	RSD [%]	16S rRNA gene content [logcopies in exract]	Mean [logcopies in extract]	SD [logcopies in extract]
7.39E+07	*7.87*	IL-DEx	8.69E+09<br>8.35E+09<br>8.44E+09	**8.49E+09**	1.78E+08	2%	9.94<br>9.92<br>9.93	9.93	0.01
		QIAGEN Kit	2.11E+09<br>1.65E+09<br>1.53E+09	**1.75E+09**	3.06E+08	17%	9.32<br>9.22<br>9.19	9.24	0.07
7.39E+06	*6.87*	IL-DEx	1.11E+09<br>1.26E+09<br>9.68E+08	**1.11E+09**	1.45E+08	13%	9.05<br>9.10<br>8.99	9.04	0.06
		QIAGEN Kit	7.52E+08<br>8.48E+08<br>7.71E+08	**7.89E+08**	5.06E+07	6%	8.88<br>8.93<br>8.89	8.90	0.03
7.39E+05	*5.87*	IL-DEx	1.31E+08<br>1.39E+08<br>9.18E+07	**1.19E+08**	2.52E+07	21%	8.12<br>8.14<br>7.96	8.07	0.10
		QIAGEN Kit	1.55E+08<br>1.19E+08<br>1.07E+08	**1.25E+08**	2.54E+07	20%	8.19<br>8.07<br>8.03	8.10	0.08
7.39E+04	*4.87*	IL-DEx	1.07E+07<br>9.69E+06<br>1.13E+07	**1.05E+07**	8.17E+05	8%	7.03<br>6.99<br>7.05	7.02	0.03
		QIAGEN Kit	9.92E+06<br>5.25E+06<br>3.70E+06	**5.77E+06**	3.24E+06	56%	7.00<br>6.72<br>6.57	6.76	0.22
7.39E+03	*3.87*	IL-DEx	9.26E+05<br>9.82E+05<br>9.12E+05	**9.39E+05**	3.70E+04	4%	5.97<br>5.99<br>5.96	5.97	0.02
		QIAGEN Kit	3.22E+05<br>1.16E+06<br>5.15E+05	**5.77E+05**	4.39E+05	76%	5.51<br>6.06<br>5.71	5.76	0.28
7.39E+02	*2.87*	IL-DEx	1.24E+05<br>8.55E+04<br>9.52E+04	**1.00E+05**	1.98E+04	20%	5.09<br>4.93<br>4.98	5.00	0.08
		QIAGEN Kit	7.27E+04<br>6.43E+04<br>5.63E+04	**6.41E+04**	8.23E+03	13%	4.86<br>4.81<br>4.75	4.81	0.06
7.39E+01	*1.87*	IL-DEx	4.96E+04<br>5.68E+04<br>4.96E+04	**5.19E+04**	4.18E+03	8%	4.70<br>4.75<br>4.70	4.71	0.03
		QIAGEN Kit	2.38E+04<br>2.28E+04<br>6.08E+04	**3.21E+04**	2.17E+04	68%	4.38<br>4.36<br>4.78	4.51	0.24
Extraction control		IL-DEx	4.83E+04<br>4.36E+04<br>4.67E+04	4.61E+04	2.41E+03	5%	4.68<br>4.64<br>4.67	4.66	0.02
		QIAGEN Kit	2.46E+04<br>6.85E+04<br>3.45E+04	3.88E+04	2.30E+04	59%	4.39<br>4.84<br>4.54	4.59	0.23
Extraction control 16S Background = Mean of Extraction control + 3xStandard deviation		IL-DEx		5.34E+04				4.73	
		QIAGEN Kit		1.08E+05				5.03	

Table S3: Data for Fig. 2. Spiking Experiments with P. aeruginosa NCTC 10662 in artificial urine. Extract from 1 ml of spiked artificial urine. Measured with 16S-qPCR. The 16S background was calculated from the mean of the extraction control + 3x standard deviation and is the limit above which values are true positives with a probability of 95%. Values of the dilutions series that fall under the limit (grey) were not included in the diagram.

CFU/ml	log CFU/ml	Method	16S rRNA gene content [copies in extract]	Mean [copies in extract]	SD [copies in extract]	RSD [%]	16S rRNA gene content [logcopies in exract]	Mean [logcopies in extract]	SD [logcopies in extract]
9.09E+07	*7.96*	IL-DEx	6.38E+09<br>7.13E+09<br>6.62E+09	**6.70E+09**	3.80E+08	6%	9.80<br>9.85<br>9.82	9.83	0.02
		QIAGEN Kit	1.12E+09<br>9.07E+08<br>1.65E+09	**1.19E+09**	3.81E+08	32%	9.05<br>8.96<br>9.22	9.07	0.13
9.09E+06	*6.96*	IL-DEx	4.14E+08<br>4.71E+08<br>4.89E+08	**4.57E+08**	3.96E+07	9%	8.62<br>8.67<br>8.69	8.66	0.04
		QIAGEN Kit	4.38E+08<br>3.93E+08<br>2.59E+08	**3.55E+08**	9.27E+07	26%	8.64<br>8.59<br>8.41	8.55	0.12
9.09E+05	*5.96*	IL-DEx	4.78E+07<br>6.15E+07<br>5.53E+07	**5.46E+07**	6.88E+06	13%	7.68<br>7.79<br>7.74	7.74	0.06
		QIAGEN Kit	4.69E+07<br>4.48E+07<br>2.42E+07	**3.70E+07**	1.26E+07	34%	7.67<br>7.65<br>7.38	7.57	0.16
9.09E+04	*4.96*	IL-DEx	4.63E+06<br>5.12E+06<br>5.11E+06	**4.95E+06**	2.81E+05	6%	6.67<br>6.71<br>6.71	6.69	0.03
		QIAGEN Kit	4.96E+06<br>2.39E+06<br>3.14E+06	**3.34E+06**	1.32E+06	40%	6.70<br>6.38<br>6.50	6.52	0.16
9.09E+03	*3.96*	IL-DEx	4.44E+05<br>4.12E+05<br>4.01E+05	**4.18E+05**	2.22E+04	5%	5.65<br>5.61<br>5.60	5.62	0.02
		QIAGEN Kit	3.58E+05<br>5.72E+05<br>4.16E+05	**4.40E+05**	1.10E+05	25%	5.55<br>5.76<br>5.62	5.64	0.10
9.09E+02	*2.96*	IL-DEx	8.40E+04<br>7.18E+04<br>9.17E+04	**8.21E+04**	1.01E+04	12%	4.92<br>4.86<br>4.96	4.91	0.05
		QIAGEN Kit	5.82E+04<br>4.72E+04<br>6.22E+04	**5.55E+04**	7.76E+03	14%	4.76<br>4.67<br>4.79	4.74	0.06
9.09E+01	*1.96*	IL-DEx	5.20E+04<br>4.41E+04<br>4.28E+04	**4.61E+04**	4.95E+03	11%	4.72<br>4.64<br>4.63	4.66	0.05
		QIAGEN Kit	1.18E+04<br>2.49E+04<br>4.64E+04	**2.39E+04**	1.75E+04	73%	4.07<br>4.40<br>4.67	4.38	0.30
Extraction control		IL-DEx	4.27E+04<br>4.03E+04<br>4.25E+04	4.18E+04	1.30E+03	3%	4.63<br>4.61<br>4.63	4.62	0.01
		QIAGEN Kit	1.99E+04<br>3.29E+04<br>3.25E+04	2.77E+04	7.42E+03	27%	4.30<br>4.52<br>4.51	4.44	0.13
Extraction control 16S Background = Mean of Extraction control + 3xStandard deviation		IL-DEx		4.58E+04				4.66	
		QIAGEN Kit		5.00E+04				4.70	

Table S4: Data for Fig. 2. Spiking Experiments with K. pneumoniae DSM 30104 in artificial urine. Extract from 1 ml of spiked artificial urine. Measured with 16S-qPCR. The 16S background was calculated from the mean of the extraction control + 3x standard deviation and is the limit above which values are true positives with a probability of 95%. Values of the dilutions series that fall under the limit (grey) were not included in the diagram.

CFU/ml	log CFU/ml	Method	16S rRNA gene content [copies in extract]	Mean [copies in extract]	SD [copies in extract]	RSD [%]	16S rRNA gene content [logcopies in exract]	Mean [logcopies in extract]	SD [logcopies in extract]
6.82E+07	*7.83*	IL-DEx	1.35E+10<br>1.41E+10<br>1.36E+10	**1.37E+10**	3.17E+08	2%	10.13<br>10.15<br>10.13	10.14	0.01
		QIAGEN Kit	2.87E+09<br>2.77E+09<br>2.98E+09	**2.87E+09**	1.06E+08	4%	9.46<br>9.44<br>9.47	9.46	0.02
6.82E+06	*6.83*	IL-DEx	1.19E+09<br>8.60E+08<br>1.29E+09	**1.10E+09**	2.27E+08	21%	9.08<br>8.93<br>9.11	9.04	0.09
		QIAGEN Kit	8.29E+08<br>7.75E+08<br>8.23E+08	**8.08E+08**	2.94E+07	4%	8.92<br>8.89<br>8.92	8.91	0.02
6.82E+05	*5.83*	IL-DEx	1.35E+08<br>9.32E+07<br>1.24E+08	**1.16E+08**	2.15E+07	19%	8.13<br>7.97<br>8.09	8.06	0.08
		QIAGEN Kit	4.32E+07<br>7.05E+07<br>5.77E+07	**5.60E+07**	1.36E+07	24%	7.64<br>7.85<br>7.76	7.75	0.11
6.82E+04	*4.83*	IL-DEx	1.06E+07<br>1.20E+07<br>1.04E+07	**1.10E+07**	8.48E+05	8%	7.02<br>7.08<br>7.02	7.04	0.03
		QIAGEN Kit	9.34E+06<br>3.52E+06<br>3.82E+06	**5.01E+06**	3.28E+06	65%	6.97<br>6.55<br>6.58	6.70	0.24
6.82E+03	*3.83*	IL-DEx	1.07E+06<br>9.74E+05<br>1.27E+06	**1.10E+06**	1.53E+05	14%	6.03<br>5.99<br>6.11	6.04	0.06
		QIAGEN Kit	6.19E+05<br>3.70E+05<br>6.74E+05	**5.37E+05**	1.62E+05	30%	5.79<br>5.57<br>5.83	5.73	0.14
6.82E+02	*2.83*	IL-DEx	1.10E+05<br>1.29E+05<br>1.03E+05	**1.13E+05**	1.35E+04	12%	5.04<br>5.11<br>5.01	5.05	0.05
		QIAGEN Kit	1.02E+05<br>1.31E+05<br>7.08E+04	**9.82E+04**	3.02E+04	31%	5.01<br>5.12<br>4.85	4.99	0.13
6.82E+01	*1.83*	IL-DEx	4.97E+04<br>4.50E+04<br>5.08E+04	**4.84E+04**	3.08E+03	6%	4.70<br>4.65<br>4.71	4.68	0.03
		QIAGEN Kit	3.16E+04<br>4.53E+04<br>2.62E+04	**3.35E+04**	9.82E+03	29%	4.50<br>4.66<br>4.42	4.52	0.12
Extraction control		IL-DEx	4.65E+04<br>4.07E+04<br>4.51E+04	4.41E+04	3.02E+03	7%	4.67<br>4.61<br>4.65	4.64	0.03
		QIAGEN Kit	1.84E+04<br>2.77E+04<br>1.57E+04	2.00E+04	6.27E+03	31%	4.27<br>4.44<br>4.20	4.30	0.13
Extraction control 16S Background = Mean of Extraction control + 3xStandard deviation		IL-DEx		5.31E+04				4.73	
		QIAGEN Kit		3.88E+04				4.59	

Table S5: Data for Fig. 2. Spiking Experiments with P. mirabilis DSM 4479 in artificial urine. Extract from 1 ml of spiked artificial urine. Measured with 16S-qPCR. The 16S background was calculated from the mean of the extraction control + 3x standard deviation and is the limit above which values are true positives with a probability of 95%. Values of the dilutions series that fall under the limit (grey) were not included in the diagram.

CFU/ml	log CFU/ml	Method	16S rRNA gene content [copies in extract]	Mean [copies in extract]	SD [copies in extract]	RSD [%]	16S rRNA gene content [logcopies in exract]	Mean [logcopies in extract]	SD [logcopies in extract]
5.45E+07	*7.74*	IL-DEx	1.02E+10<br>1.08E+10<br>1.04E+10	**1.05E+10**	3.12E+08	3%	10.01<br>10.03<br>10.02	10.02	0.01
		QIAGEN Kit	1.21E+09<br>1.35E+09<br>1.27E+09	**1.27E+09**	6.91E+07	5%	9.08<br>9.13<br>9.10	9.10	0.02
5.45E+06	*6.74*	IL-DEx	2.50E+08<br>4.33E+08<br>5.12E+08	**3.81E+08**	1.35E+08	35%	8.40<br>8.64<br>8.71	8.58	0.16
		QIAGEN Kit	2.31E+08<br>1.69E+08<br>1.70E+08	**1.88E+08**	3.56E+07	19%	8.36<br>8.23<br>8.23	8.27	0.08
5.45E+05	*5.74*	IL-DEx	8.70E+07<br>6.22E+07<br>6.80E+07	**7.17E+07**	1.29E+07	18%	7.94<br>7.79<br>7.83	7.86	0.08
		QIAGEN Kit	9.49E+06<br>1.39E+07<br>2.41E+07	**1.47E+07**	7.48E+06	51%	6.98<br>7.14<br>7.38	7.17	0.20
5.45E+04	*4.74*	IL-DEx	6.42E+06<br>6.24E+06<br>6.67E+06	**6.44E+06**	2.19E+05	3%	6.81<br>6.79<br>6.82	6.81	0.01
		QIAGEN Kit	2.33E+06<br>2.51E+06<br>4.47E+06	**2.97E+06**	1.18E+06	40%	6.37<br>6.40<br>6.65	6.47	0.15
5.45E+03	*3.74*	IL-DEx	5.08E+05<br>4.02E+05<br>6.07E+05	**4.99E+05**	1.03E+05	21%	5.71<br>5.60<br>5.78	5.70	0.09
		QIAGEN Kit	3.72E+05<br>1.94E+05<br>4.61E+05	**3.22E+05**	1.36E+05	42%	5.57<br>5.29<br>5.66	5.51	0.20
5.45E+02	*2.74*	IL-DEx	1.03E+05<br>8.75E+04<br>9.37E+04	**9.44E+04**	7.65E+03	8%	5.01<br>4.94<br>4.97	4.98	0.03
		QIAGEN Kit	6.33E+04<br>3.31E+04<br>2.97E+04	**3.96E+04**	1.85E+04	47%	4.80<br>4.52<br>4.47	4.60	0.18
5.45E+01	*1.74*	IL-DEx	7.54E+04<br>5.61E+04<br>6.15E+04	**6.38E+04**	9.94E+03	16%	4.88<br>4.75<br>4.79	4.81	0.07
		QIAGEN Kit	1.49E+04<br>9.96E+03<br>1.97E+04	**1.43E+04**	4.89E+03	34%	4.17<br>4.00<br>4.30	4.16	0.15
Extraction control		IL-DEx	3.13E+04<br>5.20E+04<br>5.35E+04	4.43E+04	1.24E+04	28%	4.50<br>4.72<br>4.73	4.65	0.13
		QIAGEN Kit	1.52E+04<br>7.40E+03<br>4.23E+03	7.80E+03	5.63E+03	72%	4.18<br>3.87<br>3.63	3.89	0.28
Extraction control 16S Background = Mean of Extraction control + 3xStandard deviation		IL-DEx		8.17E+04				4.91	
		QIAGEN Kit		2.47E+04				4.39	

Table S6: Data for Fig. 2. Spiking Experiments with E. faecalis DSM 20478 in artificial urine. Extract from 1 ml of spiked artificial urine. Measured with 16S-qPCR. The 16S background was calculated from the mean of the extraction control + 3x standard deviation and is the limit above which values are true positives with a probability of 95%. Values of the dilutions series that fall under the limit (grey) were not included in the diagram.

CFU/ml	log CFU/ml	Method	16S rRNA gene content [copies in extract]	Mean [copies in extract]	SD [copies in extract]	RSD [%]	16S rRNA gene content [logcopies in exract]	Mean [logcopies in extract]	SD [logcopies in extract]
5.94E+07	*7.77*	IL-DEx	2.40E+08<br>2.74E+08<br>2.85E+08	**2.66E+08**	2.37E+07	9%	8.38<br>8.44<br>8.46	8.42	0.04
		QIAGEN Kit	9.92E+08<br>1.50E+09<br>1.84E+09	**1.40E+09**	4.25E+08	30%	9.00<br>9.18<br>9.26	9.15	0.14
5.94E+06	*6.77*	IL-DEx	1.82E+07<br>1.93E+07<br>3.83E+07	**2.38E+07**	1.13E+07	48%	7.26<br>7.29<br>7.58	7.38	0.18
		QIAGEN Kit	1.20E+09<br>4.82E+08<br>6.50E+08	**7.22E+08**	3.77E+08	52%	9.08<br>8.68<br>8.81	8.86	0.20
5.94E+05	*5.77*	IL-DEx	2.14E+06<br>2.17E+06<br>1.84E+06	**2.04E+06**	1.83E+05	9%	6.33<br>6.34<br>6.26	6.31	0.04
		QIAGEN Kit	4.53E+07<br>1.96E+07<br>5.60E+07	**3.68E+07**	1.87E+07	51%	7.66<br>7.29<br>7.75	7.57	0.24
5.94E+04	*4.77*	IL-DEx	2.32E+05<br>2.21E+05<br>2.71E+05	**2.41E+05**	2.62E+04	11%	5.37<br>5.35<br>5.43	5.38	0.05
		QIAGEN Kit	9.31E+05<br>6.54E+06<br>1.03E+07	**3.97E+06**	4.72E+06	119%	5.97<br>6.82<br>7.01	6.60	0.55
5.94E+03	*3.77*	IL-DEx	5.97E+04<br>5.97E+04<br>5.54E+04	**5.82E+04**	2.45E+03	4%	4.78<br>4.78<br>4.74	4.77	0.02
		QIAGEN Kit	5.66E+05<br>9.51E+05<br>7.85E+05	**7.50E+05**	1.93E+05	26%	5.75<br>5.98<br>5.89	5.88	0.11
5.94E+02	*2.77*	IL-DEx	4.17E+04<br>4.25E+04<br>4.02E+04	**4.15E+04**	1.16E+03	3%	4.62<br>4.63<br>4.60	4.62	0.01
		QIAGEN Kit	5.91E+04<br>5.63E+04<br>5.41E+04	**5.65E+04**	2.52E+03	4%	4.77<br>4.75<br>4.73	4.75	0.02
5.94E+01	*1.77*	IL-DEx	4.01E+04<br>3.95E+04<br>3.98E+04	**3.98E+04**	2.94E+02	1%	4.60<br>4.60<br>4.60	4.60	0.00
		QIAGEN Kit	3.03E+04<br>3.20E+04<br>4.10E+04	**3.41E+04**	5.76E+03	17%	4.48<br>4.50<br>4.61	4.53	0.07
Extraction control		IL-DEx	4.22E+04<br>4.48E+04<br>3.64E+04	4.10E+04	4.28E+03	10%	4.63<br>4.65<br>4.56	4.61	0.05
		QIAGEN Kit	3.59E+04<br>1.90E+04<br>2.68E+04	2.64E+04	8.47E+03	32%	4.56<br>4.28<br>4.43	4.42	0.14
Extraction control 16S Background = Mean of Extraction control + 3xStandard deviation		IL-DEx		5.38E+04				4.73	
		QIAGEN Kit		5.18E+04				4.71	

Table S7: Data for Fig. 2. Spiking Experiments with E. faecium DSM 20477 in artificial urine. Extract from 1 ml of spiked artificial urine. Measured with 16S-qPCR. The 16S background was calculated from the mean of the extraction control + 3x standard deviation and is the limit above which values are true positives with a probability of 95%. Values of the dilutions series that fall under the limit (grey) were not included in the diagram.

CFU/ml	log CFU/ml	Method	16S rRNA gene content [copies in extract]	Mean [copies in extract]	SD [copies in extract]	RSD [%]	16S rRNA gene content [logcopies in exract]	Mean [logcopies in extract]	SD [logcopies in extract]
1.13E+08	*8.05*	IL-DEx	2.73E+09<br>2.73E+09<br>3.89E+09	**3.07E+09**	6.71E+08	22%	9.44<br>9.44<br>9.59	9.49	0.09
		QIAGEN Kit	2.11E+09<br>2.00E+09<br>2.50E+09	**2.20E+09**	2.63E+08	12%	9.33<br>9.30<br>9.40	9.34	0.05
1.13E+07	*7.05*	IL-DEx	1.95E+08<br>1.84E+08<br>1.76E+08	**1.85E+08**	9.53E+06	5%	8.29<br>8.26<br>8.25	8.27	0.02
		QIAGEN Kit	7.94E+08<br>8.30E+08<br>1.05E+09	**8.83E+08**	1.36E+08	15%	8.90<br>8.92<br>9.02	8.95	0.06
1.13E+06	*6.05*	IL-DEx	1.23E+07<br>1.37E+07<br>1.11E+07	**1.23E+07**	1.31E+06	11%	7.09<br>7.14<br>7.05	7.09	0.05
		QIAGEN Kit	3.84E+07<br>1.19E+08<br>1.82E+08	**9.40E+07**	7.19E+07	76%	7.58<br>8.08<br>8.26	7.97	0.35
1.13E+05	*5.05*	IL-DEx	6.31E+05<br>8.80E+05<br>9.75E+05	**8.15E+05**	1.77E+05	22%	5.80<br>5.94<br>5.99	5.91	0.10
		QIAGEN Kit	1.08E+07<br>1.59E+07<br>8.13E+06	**1.12E+07**	3.95E+06	35%	7.03<br>7.20<br>6.91	7.05	0.15
1.13E+04	*4.05*	IL-DEx	1.01E+05<br>8.69E+04<br>9.16E+04	**9.30E+04**	7.22E+03	8%	5.00<br>4.94<br>4.96	4.97	0.03
		QIAGEN Kit	3.26E+05<br>1.36E+06<br>9.97E+05	**7.61E+05**	5.23E+05	69%	5.51<br>6.13<br>6.00	5.88	0.33
1.13E+03	*3.05*	IL-DEx	3.80E+04<br>4.39E+04<br>5.04E+04	**4.38E+04**	6.23E+03	14%	4.58<br>4.64<br>4.70	4.64	0.06
		QIAGEN Kit	7.90E+04<br>1.17E+05<br>8.50E+04	**9.24E+04**	2.07E+04	22%	4.90<br>5.07<br>4.93	4.97	0.09
1.13E+02	*2.05*	IL-DEx	3.60E+04<br>4.89E+04<br>3.69E+04	**4.02E+04**	7.22E+03	18%	4.56<br>4.69<br>4.57	4.60	0.07
		QIAGEN Kit	2.42E+04<br>1.74E+04<br>1.10E+04	**1.67E+04**	6.64E+03	40%	4.38<br>4.24<br>4.04	4.22	0.17
Extraction control		IL-DEx	4.35E+04<br>3.95E+04<br>3.77E+04	4.02E+04	2.99E+03	7%	4.64<br>4.60<br>4.58	4.60	0.03
		QIAGEN Kit	5.14E+04<br>6.67E+04<br>2.20E+04	4.23E+04	2.27E+04	54%	4.71<br>4.82<br>4.34	4.63	0.25
Extraction control 16S Background = Mean of Extraction control + 3xStandard deviation		IL-DEx		4.91E+04				4.69	
		QIAGEN Kit		1.10E+05				5.04	

Table S8: Data for Fig. 2. Spiking Experiments with S. saprophyticus DSM 20229 in artificial urine. Extract from 1 ml of spiked artificial urine. Measured with 16S-qPCR. The 16S background was calculated from the mean of the extraction control + 3x standard deviation and is the limit above which values are true positives with a probability of 95%. Values of the dilutions series that fall under the limit (grey) were not included in the diagram.

CFU/ml	log CFU/ml	Method	16S rRNA gene content [copies in extract]	Mean [copies in extract]	SD [copies in extract]	RSD [%]	16S rRNA gene content [logcopies in exract]	Mean [logcopies in extract]	SD [logcopies in extract]
3.00E+07	*7.48*	IL-DEx	8.67E+07<br>9.23E+07<br>1.05E+08	**9.44E+07**	9.50E+06	10%	7.94<br>7.96<br>8.02	7.98	0.04
		QIAGEN Kit	2.01E+09<br>2.08E+09<br>2.06E+09	**2.05E+09**	3.39E+07	2%	9.30<br>9.32<br>9.31	9.31	0.01
3.00E+06	*6.48*	IL-DEx	9.96E+06<br>9.09E+06<br>1.01E+07	**9.70E+06**	5.48E+05	6%	7.00<br>6.96<br>7.00	6.99	0.02
		QIAGEN Kit	2.54E+08<br>4.49E+08<br>5.35E+08	**3.93E+08**	1.44E+08	37%	8.40<br>8.65<br>8.73	8.59	0.17
3.00E+05	*5.48*	IL-DEx	7.43E+05<br>8.85E+05<br>9.88E+05	**8.66E+05**	1.23E+05	14%	5.87<br>5.95<br>5.99	5.94	0.06
		QIAGEN Kit	3.11E+07<br>2.75E+07<br>1.41E+07	**2.29E+07**	8.96E+06	39%	7.49<br>7.44<br>7.15	7.36	0.19
3.00E+04	*4.48*	IL-DEx	9.15E+04<br>1.26E+05<br>1.44E+05	**1.19E+05**	2.69E+04	23%	4.96<br>5.10<br>5.16	5.07	0.10
		QIAGEN Kit	1.70E+06<br>2.86E+06<br>3.29E+06	**2.52E+06**	8.21E+05	33%	6.23<br>6.46<br>6.52	6.40	0.15
3.00E+03	*3.48*	IL-DEx	7.01E+04<br>6.01E+04<br>5.06E+04	**5.97E+04**	9.72E+03	16%	4.85<br>4.78<br>4.70	4.78	0.07
		QIAGEN Kit	1.29E+05<br>2.12E+05<br>1.28E+05	**1.52E+05**	4.84E+04	32%	5.11<br>5.33<br>5.11	5.18	0.13
3.00E+02	*2.48*	IL-DEx	3.84E+04<br>3.92E+04<br>5.57E+04	**4.38E+04**	9.75E+03	22%	4.58<br>4.59<br>4.75	4.64	0.09
		QIAGEN Kit	7.01E+04<br>6.27E+04<br>2.43E+04	**4.74E+04**	2.46E+04	52%	4.85<br>4.80<br>4.39	4.68	0.25
3.00E+01	*1.48*	IL-DEx	4.10E+04<br>3.89E+04<br>5.01E+04	**4.31E+04**	5.94E+03	14%	4.61<br>4.59<br>4.70	4.63	0.06
		QIAGEN Kit	3.44E+04<br>9.79E+03<br>2.14E+04	**1.93E+04**	1.23E+04	64%	4.54<br>3.99<br>4.33	4.29	0.28
Extraction control		IL-DEx	3.78E+04<br>3.94E+04<br>4.60E+04	4.09E+04	4.38E+03	11%	4.58<br>4.60<br>4.66	4.61	0.05
		QIAGEN Kit	3.07E+04<br>6.60E+03<br>2.45E+04	1.71E+04	1.25E+04	73%	4.49<br>3.82<br>4.39	4.23	0.36
Extraction control 16S Background = Mean of Extraction control + 3xStandard deviation		IL-DEx		5.41E+04				4.73	
		QIAGEN Kit		5.47E+04				4.74	

Table S9: Data for Fig. S1. Spiking Experiments with E. coli NCTC 9001 in artificial urine. Extract from 1 ml of spiked artificial urine. Measured with E.coli-qPCR. The 23S background was calculated from the mean of the extraction control + 3x standard deviation and is the limit above which values are true positives with a probability of 95%. Values of the dilutions series that fall under the limit (grey) were not included in the diagram.

CFU/ml	log CFU/ml	Method	23S rRNA gene content [copies in extract]	Mean [copies in extract]	SD [copies in extract]	RSD [%]	23S rRNA gene content [logcopies in exract]	Mean [logcopies in extract]	SD [logcopies in extract]
7.39E+07	*7.87*	IL-DEx	2.62E+09<br>2.19E+09<br>2.56E+09	**2.45E+09**	2.33E+08	10%	9.42<br>9.34<br>9.41	9.39	0.04
		QIAGEN Kit	8.43E+08<br>7.95E+08<br>1.02E+09	**8.81E+08**	1.18E+08	13%	8.93<br>8.90<br>9.01	8.94	0.06
7.39E+06	*6.87*	IL-DEx	3.07E+08<br>2.32E+08<br>2.10E+08	**2.46E+08**	5.09E+07	21%	8.49<br>8.36<br>8.32	8.39	0.09
		QIAGEN Kit	3.50E+08<br>3.27E+08<br>2.80E+08	**3.17E+08**	3.58E+07	11%	8.54<br>8.51<br>8.45	8.50	0.05
7.39E+05	*5.87*	IL-DEx	4.09E+07<br>2.59E+07<br>1.59E+07	**2.56E+07**	1.26E+07	49%	7.61<br>7.41<br>7.20	7.41	0.21
		QIAGEN Kit	4.55E+07<br>6.33E+07<br>4.75E+07	**5.15E+07**	9.75E+06	19%	7.66<br>7.80<br>7.68	7.71	0.08
7.39E+04	*4.87*	IL-DEx	4.04E+06<br>4.37E+06<br>3.85E+06	**4.08E+06**	2.60E+05	6%	6.61<br>6.64<br>6.59	6.61	0.03
		QIAGEN Kit	6.26E+06<br>1.27E+06<br>6.84E+05	**1.76E+06**	3.06E+06	174%	6.80<br>6.10<br>5.84	6.25	0.50
7.39E+03	*3.87*	IL-DEx	4.24E+05<br>3.82E+05<br>3.64E+05	**3.89E+05**	3.08E+04	8%	5.63<br>5.58<br>5.56	5.59	0.03
		QIAGEN Kit	4.65E+04<br>5.23E+05<br>7.15E+04	**1.20E+05**	2.68E+05	223%	4.67<br>5.72<br>4.85	5.08	0.56
7.39E+02	*2.87*	IL-DEx	2.53E+04<br>2.87E+04<br>4.12E+04	**3.10E+04**	8.38E+03	27%	4.40<br>4.46<br>4.61	4.49	0.11
		QIAGEN Kit	1.97E+04<br>9.42E+03<br>8.17E+03	**1.15E+04**	6.32E+03	55%	4.29<br>3.97<br>3.91	4.06	0.21
7.39E+01	*1.87*	IL-DEx	3.94E+03<br>4.09E+03<br>4.06E+03	**4.03E+03**	7.96E+01	2%	3.60<br>3.61<br>3.61	3.60	0.01
		QIAGEN Kit	3.60E+03<br>2.82E+03<br>5.59E+03	**3.84E+03**	1.43E+03	37%	3.56<br>3.45<br>3.75	3.58	0.15
Extraction control		IL-DEx	1.47E+02<br>4.45E+02<br>3.22E+02	2.76E+02	1.50E+02	54%	2.17<br>2.65<br>2.51	2.44	0.25
		QIAGEN Kit	3.39E+03<br>1.17E+03<br>1.12E+03	1.64E+03	1.30E+03	79%	3.53<br>3.07<br>3.05	3.22	0.27
23S Background = Mean of Extraction control + 3xStandard deviation		IL-DEx		7.26E+02				2.86	
		QIAGEN Kit		5.54E+03				3.74	

Table S10: Data for Fig. S1. Spiking Experiments with E. faecalis DSM 20478 in artificial urine. Extract from 1 ml of spiked artificial urine. Measured with Enterococcus-qPCR.

CFU/ml	log CFU/ml	Method	23S rRNA gene content [copies in extract]	Mean [copies in extract]	SD [copies in extract]	RSD [%]	23S rRNA gene content [logcopies in exract]	Mean [logcopies in extract]	SD [logcopies in extract]
5.94E+07	*7.77*	IL-DEx	6.20E+07	**6.65E+07**	2.19E+07	33%	7.79	7.82	0.13
			5.09E+07				7.71		
			9.31E+07				7.97		
		QIAGEN Kit	5.12E+08	**7.92E+08**	4.25E+08	54%	8.71	8.90	0.21
			7.29E+08				8.86		
			1.33E+09				9.12		
5.94E+06	*6.77*	IL-DEx	5.77E+06	**9.59E+06**	3.87E+06	40%	6.76	6.98	0.19
			1.30E+07				7.11		
			1.18E+07				7.07		
		QIAGEN Kit	3.27E+08	**2.57E+08**	5.80E+07	23%	8.51	8.41	0.09
			2.40E+08				8.38		
			2.16E+08				8.34		
5.94E+05	*5.77*	IL-DEx	1.25E+06	**1.06E+06**	1.72E+05	16%	6.10	6.02	0.07
			9.02E+05				5.96		
			1.05E+06				6.02		
		QIAGEN Kit	1.20E+07	**9.09E+06**	2.46E+06	27%	7.08	6.96	0.11
			7.22E+06				6.86		
			8.67E+06				6.94		
5.94E+04	*4.77*	IL-DEx	7.74E+04	**1.07E+05**	3.64E+04	34%	4.89	5.03	0.14
			1.50E+05				5.18		
			1.07E+05				5.03		
		QIAGEN Kit	1.32E+05	**1.21E+06**	2.86E+06	236%	5.12	6.08	0.86
			2.31E+06				6.36		
			5.81E+06				6.76		
5.94E+03	*3.77*	IL-DEx	1.31E+04	**1.87E+04**	6.76E+03	36%	4.12	4.27	0.15
			2.66E+04				4.42		
			1.90E+04				4.28		
		QIAGEN Kit	2.46E+05	**3.35E+05**	8.53E+04	25%	5.39	5.53	0.12
			4.07E+05				5.61		
			3.77E+05				5.58		
5.94E+02	*2.77*	IL-DEx	2.28E+03	**1.53E+03**	8.86E+02	58%	3.36	3.18	0.25
			1.02E+03				3.01		
			n.d.				n.d.		
		QIAGEN Kit	1.55E+04	**2.22E+04**	7.96E+03	36%	4.19	4.35	0.15
			2.27E+04				4.36		
			3.14E+04				4.50		
5.94E+01	*1.77*	IL-DEx	n.d.	**n.d.**			n.d.	n.d.	
			n.d.				n.d.		
			n.d.				n.d.		
		QIAGEN Kit	2.23E+03	**3.43E+03**	2.17E+03	63%	3.35	3.54	0.27
			n.d.				n.d.		
			5.29E+03				3.72		
Extraction control		IL-DEx	n.d.	n.d.			n.d.	n.d.	
			n.d.				n.d.		
			n.d.				n.d.		
		QIAGEN Kit	n.d.	n.d.			n.d.	n.d.	
			n.d.				n.d.		
			n.d.				n.d.		

Table S11: Data for Fig. S1. Spiking Experiments with E. faecium DSM 20477 in artificial urine. Extract from 1 ml of spiked artificial urine. Measured with Enterococcus-qPCR.

CFU/ml	log CFU/ml	Method	23S rRNA gene content [copies in extract]	Mean [copies in extract]	SD [copies in extract]	RSD [%]	23S rRNA gene content [logcopies in exract]	Mean [logcopies in extract]	SD [logcopies in extract]
1.13E+08	*8.05*	IL-DEx	6.40E+08<br>3.75E+08<br>1.02E+09	**6.25E+08**	3.23E+08	52%	8.81<br>8.57<br>9.01	8.80	0.22
		QIAGEN Kit	6.61E+08<br>8.63E+08<br>1.05E+09	**8.43E+08**	1.94E+08	23%	8.82<br>8.94<br>9.02	8.93	0.10
1.13E+07	*7.05*	IL-DEx	3.49E+07<br>6.08E+07<br>5.72E+07	**4.95E+07**	1.40E+07	28%	7.54<br>7.78<br>7.76	7.69	0.13
		QIAGEN Kit	3.36E+08<br>2.07E+08<br>1.05E+09	**4.18E+08**	4.54E+08	109%	8.53<br>8.32<br>9.02	8.62	0.36
1.13E+06	*6.05*	IL-DEx	2.51E+06<br>3.91E+06<br>3.35E+06	**3.20E+06**	7.05E+05	22%	6.40<br>6.59<br>6.52	6.51	0.10
		QIAGEN Kit	9.65E+06<br>5.13E+07<br>6.77E+07	**3.22E+07**	2.99E+07	93%	6.98<br>7.71<br>7.83	7.51	0.46
1.13E+05	*5.05*	IL-DEx	2.32E+05<br>2.43E+05<br>3.44E+05	**2.69E+05**	6.15E+04	23%	5.37<br>5.39<br>5.54	5.43	0.09
		QIAGEN Kit	5.99E+06<br>8.49E+06<br>2.39E+06	**4.96E+06**	3.06E+06	62%	6.78<br>6.93<br>6.38	6.70	0.28
1.13E+04	*4.05*	IL-DEx	3.02E+04<br>4.14E+04<br>7.04E+04	**4.45E+04**	2.08E+04	47%	4.48<br>4.62<br>4.85	4.65	0.19
		QIAGEN Kit	9.56E+04<br>4.10E+05<br>5.06E+05	**2.71E+05**	2.15E+05	79%	4.98<br>5.61<br>5.70	5.43	0.39
1.13E+03	*3.05*	IL-DEx	4.32E+03<br>3.60E+03<br>4.88E+03	**4.23E+03**	6.43E+02	15%	3.64<br>3.56<br>3.69	3.63	0.07
		QIAGEN Kit	5.22E+04<br>4.51E+04<br>4.59E+04	**4.77E+04**	3.88E+03	8%	4.72<br>4.65<br>4.66	4.68	0.03
1.13E+02	*2.05*	IL-DEx	n.d.<br>n.d.<br>n.d.	**n.d.**			n.d.<br>n.d.<br>n.d.	n.d.	
		QIAGEN Kit	3.81E+03<br>3.57E+03<br>n.d.	**3.69E+03**	1.76E+02	5%	3.58<br>3.55<br>n.d.	3.57	0.02
Extraction control		IL-DEx	n.d.<br>n.d.<br>n.d.	n.d.			n.d.<br>n.d.<br>n.d.	n.d.	
		QIAGEN Kit	n.d.<br>n.d.<br>n.d.	n.d.			n.d.<br>n.d.<br>n.d.	n.d.	

Table S12: Data for Fig. 3 and Fig. S3. Spiking Experiments with E. coli isolate IHM 5531 in five non-UTI urines. Extract from 1 ml of spiked urine. Measured with 16S-qPCR. The 16S background was calculated from the mean of the extraction control + 3x standard deviation and is the limit above which values are true positives with a probability of 95%.

Urine unspiked													
**Method**	**Gender**	**Sample**	**16S rRNA gene content [copies in extract]**			**Mean [copies in extract]**	**SD [copies in extract]**	**RSD [%]**	**16S rRNA gene content [logcopies in extract]**			**Mean [logcopies in extract]**	**SD [logcopies in extract]**
IL-DEx	w	Urine 1 ♀	1.59E+08	1.38E+08	1.67E+08	**1.54E+08**	1.51E+07	10%	8.20	8.14	8.22	8.19	0.04
	w	Urine 2 ♀	1.66E+06	1.16E+06	1.74E+06	**1.49E+06**	3.14E+05	21%	6.22	6.06	6.24	6.17	0.10
	m	Urine 3 ♂	6.79E+05	9.45E+05	7.59E+05	**7.87E+05**	1.37E+05	17%	5.83	5.98	5.88	5.90	0.07
	m	Urine 4 ♂	1.75E+06	1.91E+06	2.23E+06	**1.95E+06**	2.46E+05	13%	6.24	6.28	6.35	6.29	0.05
	w	Urine 5 ♀	5.79E+06	5.23E+06	4.68E+06	**5.21E+06**	5.56E+05	11%	6.76	6.72	6.67	6.72	0.05
QIAGEN kit	w	Urine 1 ♀	1.11E+08	8.10E+07	1.05E+08	**9.80E+07**	1.58E+07	16%	8.04	7.91	8.02	7.99	0.07
	w	Urine 2 ♀	4.69E+08	2.13E+08	3.03E+08	**3.11E+08**	1.30E+08	42%	8.67	8.33	8.48	8.49	0.17
	m	Urine 3 ♂	1.54E+06	1.31E+06	1.20E+06	**1.34E+06**	1.70E+05	13%	6.19	6.12	6.08	6.13	0.05
	m	Urine 4 ♂	6.17E+06*	1.56E+06	2.31E+06	**1.90E+06**	5.35E+05	28%		6.19	6.36	6.28	0.12
	w	Urine 5 ♀	2.36E+08	3.24E+08	2.37E+08	**2.63E+08**	5.06E+07	19%	8.37	8.51	8.37	8.42	0.08
**Urine spiked**													
**Method**	**Gender**	**Sample**	**16S rRNA gene content [copies in extract]**			**Mean [copies in extract]**	**SD [copies in extract]**	**RSD [%]**	**16S rRNA gene content [logcopies in extract]**			**Mean [logcopies in extract]**	**SD [logcopies in extract]**
IL-DEx	-	E. coli Spike	1.04E+09	6.88E+08	6.81E+08	**7.87E+08**	2.05E+08	26%	9.02	8.84	8.83	8.90	0.10
	w	Urine 1 ♀	missing	1.13E+09	1.24E+09	**1.18E+09**	8.21E+07	7%	mssing	9.05	9.09	9.07	0.03
	w	Urine 2 ♀	5.81E+08	2.58E+08	4.01E+08	**3.92E+08**	1.62E+08	41%	8.76	8.41	8.60	8.59	0.18
	m	Urine 3 ♂	9.08E+08	6.54E+08	3.19E+08	**5.75E+08**	2.95E+08	51%	8.96	8.82	8.50	8.76	0.23
	m	Urine 4 ♂	1.43E+09	1.05E+09	1.22E+09	**1.22E+09**	1.91E+08	16%	9.15	9.02	9.09	9.09	0.07
	w	Urine 5 ♀	1.31E+09	1.02E+09	1.10E+09	**1.13E+09**	1.51E+08	13%	9.12	9.01	9.04	9.05	0.06
QIAGEN kit	-	E. coli Spike	4.75E+08	5.76E+08	2.85E+08	**4.27E+08**	1.48E+08	35%	8.68	8.76	8.46	8.63	0.16
	w	Urine 1 ♀	1.63E+09	1.72E+09	1.60E+09	**1.65E+09**	6.42E+07	4%	9.21	9.24	9.20	9.22	0.02
	w	Urine 2 ♀	1.55E+09	7.41E+08	1.07E+09	**1.07E+09**	4.05E+08	38%	9.19	8.87	9.03	9.03	0.16
	m	Urine 3 ♂	9.10E+08	6.34E+08	7.80E+08	**7.67E+08**	1.38E+08	18%	8.96	8.80	8.89	8.88	0.08
	m	Urine 4 ♂	9.89E+08	1.06E+09	9.89E+08	**1.01E+09**	3.86E+07	4%	9.00	9.02	9.00	9.00	0.02
	w	Urine 5 ♀	1.38E+09	8.13E+08	2.08E+09	**1.32E+09**	6.35E+08	48%	9.14	8.91	9.32	9.12	0.20
**Extraction control**													
**Method**	**Gender**	**Sample**	**16S rRNA gene content [copies in extract]**			**Mean [copies in extract]**	**SD [copies in extract]**	**RSD [%]**	**16S rRNA gene content [logcopies in extract]**			**Mean [logcopies in extract]**	**SD**
IL-DEx	-	Extraction control	7.34E+04	5.87E+04	5.38E+04	6.14E+04	1.02E+04	17%	4.87	4.77	4.73	4.79	0.07
QIAGEN kit	-	Extraction control	1.28E+04	2.13E+04	4.95E+04	2.38E+04	1.92E+04	81%	4.11	4.33	4.69	4.38	0.30
IL-DEx	-	Extraction control 16S Background = Mean of Extraction control + 3xStandard deviation	-	-	-	9.20E+04	-	-	-	-	-	4.96	-
QIAGEN kit	-	Extraction control 16S Background = Mean of Extraction control + 3xStandard deviation	-	-	-	8.14E+04	-	-	-	-	-	4.91	-

* outlier removed

Table S13: Data for Fig. S2. Human DNA in five non-UTI urines from the E. coli Spiking Experiments. Extract from 1 ml of spiked urine. Measured with human-qPCR.

Urine unspiked								
**Method**	**Gender**	**Sample**	**human DNA content [ng in extract]**			**Mean [ng in extract]**	**SD [ng in extract]**	**RSD [%]**
IL-DEx	w	Urine 1 ♀	203.10	326.71	675.96	**355.30**	245.24	69%
	w	Urine 2 ♀	2563.34	1197.31	1685.15	**1729.36**	692.25	40%
	m	Urine 3 ♂	86.15	101.89	25.89	**61.02**	40.12	66%
	m	Urine 4 ♂	132.28	197.50	284.08	**195.06**	76.15	39%
	w	Urine 5 ♀	285.85	692.97	930.91	**569.19**	326.20	57%
QIAGEN kit	w	Urine 1 ♀	72.87	93.14	148.90	**100.35**	39.37	39%
	w	Urine 2 ♀	1461.11	538.93	911.12	**895.22**	463.94	52%
	m	Urine 3 ♂	24.03	17.78	6.98	**14.39**	8.63	60%
	m	Urine 4 ♂	167.04*	6.11	10.55	**8.03**	3.14	39%
	w	Urine 5 ♀	239.52	1223.97	695.31	**588.52**	492.67	84%
**Extraction control**								
**Method**	**Gender**	**Sample**	**human DNA content [ng in extract]**			**Mean [ng in extract]**	**SD [ng in extract]**	**RSD [%]**
IL-DEx	-	Extraction control	0.014	0.009	0.011	0.01	0.002	20%
QIAGEN kit	-	Extraction control	0.014	0.016	0.013	0.01	0.001	10%

Table S14: Data for Fig. S4 and S5. Spiking Experiments with E. faecalis DSM 20487 in a non-UTI urine. Extract from 1 ml of spiked urine. Measured with Enterococcus-qPCR.

Urine unspiked														
**Method**	**Gender**	**Sample**	**23S rRNA gene content [copies in extract]**			**Mean [copies in extract]**	**SD [copies in extract]**	**RSD [%]**	**23S rRNA gene content [logcopies in extract]**			**Mean [logcopies in extract]**	**SD [logcopies in extract]**
IL-DEx	w	Urine ♀	2.36E+04	4.03E+04	2.76E+04	**2.97E+04**	8.68E+03	29%	4.37	4.61	4.44	4.47	0.12
QIAGEN kit	w	Urine ♀	7.51E+03	6.02E+04	6.37E+04	**3.07E+04**	3.15E+04	103%	3.88	4.78	4.80	4.49	0.53
**Urine spiked**													
**Method**	**Gender**	**Sample**	**23S rRNA gene content [copies in extract]**			**Mean [copies in extract]**	**SD [copies in extract]**	**RSD [%]**	**23S rRNA gene content [logcopies in extract]**			**Mean [logcopies in extract]**	**SD [logcopies in extract]**
IL-DEx	-	E. faecalis Spike	5.31E+07	2.96E+07	1.75E+07	**3.02E+07**	1.81E+07	60%	7.73	7.47	7.24	7.48	0.24
	w	Urine ♀	5.02E+06	8.09E+06	4.75E+06	**5.78E+06**	1.85E+06	32%	6.70	6.91	6.68	6.76	0.13
QIAGEN kit	-	E. faecalis Spike	3.52E+08	3.34E+08	1.36E+08	**2.52E+08**	1.20E+08	47%	8.55	8.52	8.13	8.40	0.23
	w	Urine ♀	4.49E+08	1.02E+09	7.05E+08	**6.86E+08**	2.86E+08	42%	8.65	9.01	8.85	8.84	0.18
**Extraction control**													
**Method**	**Gender**	**Sample**	**23S rRNA gene content [copies in extract]**			**Mean [copies in extract]**	**SD [copies in extract]**	**RSD [%]**	**23S rRNA gene content [logcopies in extract]**			**Mean [logcopies in extract]**	**SD [logcopies in extract]**
IL-DEx	-	Extraction control	n.d.	n.d.	n.d.								
QIAGEN kit	-	Extraction control	n.d.	n.d.	n.d.								

Table S15: Data for Fig. 4A. Total nucleic acid content of extracts from clinical urines. Extract from 1 ml of urine. Measured with Nanodrop.

Method → Sample ↓	IL-DEx Nucleic Acid [ng in extract]	Mean [ng in extract]	A260/A280	Mean	A260/A230	Mean
1A	888.50	865.65	1.27	1.28	0.13	0.13
1B	842.80		1.29		0.12	
2A	4700.20	3777.80	1.77	1.75	0.11	0.12
2B	2855.40		1.73		0.13	
3A	5984.10	6223.15	1.88	1.88	0.42	0.42
3B	6462.20		1.88		0.42	
4A	1172.80	1211.30	1.67	1.65	0.16	0.14
4B	1249.80		1.62		0.12	
5A	3420.60	3118.90	2.03	1.98	0.35	0.30
5B	2817.20		1.94		0.25	
6A	8696.50	9386.90	1.94	1.94	0.55	0.50
6B	10077.30		1.94		0.46	
7A	8084.50	7584.10	1.92	1.92	0.59	0.65
7B	7083.70		1.91		0.72	
8A	7753.30	9968.00	1.72	1.79	0.66	0.79
8B	12182.70		1.86		0.92	
9A	7455.10	9167.80	1.18	0.84	0.25	0.29
9B	10880.50		0.50		0.34	
10A	1154.60	1181.40	1.67	1.69	0.21	0.24
10B	1208.20		1.71		0.26	
11A	492.50	394.95	1.59	1.52	0.08	0.07
11B	297.40		1.46		0.05	
12A	261.30	235.60	0.67	0.67	0.05	0.06
12B	209.90		0.67		0.06	
13A	299.80	247.95	0.67	0.69	0.07	0.07
13B	196.10		0.70		0.07	

Method → Sample ↓	QIAGEN Nucleic Acid [ng in extract]	Mean [ng in extract]	A260/A280	Mean	A260/A230	Mean
1A	774.10	667.55	1.32	1.52	0.16	0.21
1B	561.00		1.71		0.27	
2A	2157.80	2036.50	1.93	1.95	0.64	0.67
2B	1915.20		1.97		0.70	
3A	1592.10	1594.60	2.17	2.15	1.07	0.95
3B	1597.10		2.13		0.83	
4A	871.70	964.75	1.92	1.87	0.59	0.47
4B	1057.80		1.83		0.35	
5A	355.30	684.90	2.13	1.86	0.25	0.25
5B	1014.50		1.59		0.26	
6A	598.00	1251.75	2.27	2.16	1.26	1.07
6B	1905.50		2.05		0.89	
7A	1169.60	1532.20	2.20	2.11	1.12	1.07
7B	1894.80		2.01		1.02	
8A	4912.50	5419.40	2.03	1.97	1.79	1.67
8B	5926.30		1.92		1.54	
9A	3380.00	2920.00	1.13	1.52	0.79	0.75
9B	2460.00		1.92		0.70	
10A	846.40	971.80	1.88	1.93	0.32	0.60
10B	1097.20		1.97		0.87	
11A	514.00	574.40	1.07	1.14	0.30	0.32
11B	634.80		1.20		0.34	
12A	1413.90	1297.60	1.71	1.73	0.74	0.73
12B	1181.30		1.75		0.72	
13A	630.80	672.75	1.53	1.55	0.32	0.33
13B	714.70		1.57		0.34	

Method → Sample ↓	Promega Nucleic Acid [ng in extract]	Mean [ng in extract]	A260/A280	Mean	A260/A230	Mean
1A	2379.50	2407.40	1.56	1.56	1.12	1.11
1B	2435.30		1.55		1.10	
2A	24616.10	23427.10	1.84	1.84	1.79	1.80
2B	22238.10		1.85		1.82	
3A	11345.00	10390.70	1.76	1.76	1.57	1.56
3B	9436.40		1.76		1.54	
4A	3084.90	3126.75	1.61	1.62	1.17	1.27
4B	3168.60		1.62		1.38	
5A	2771.00	2751.90	1.63	1.66	1.20	1.14
5B	2732.80		1.69		1.08	
6A	12656.70	12453.70	1.89	1.89	1.55	1.54
6B	12250.70		1.89		1.52	
7A	9171.50	8696.15	1.76	1.77	1.38	1.39
7B	8220.80		1.78		1.39	
8A	9268.60	9298.50	0.96	1.02	0.69	0.74
8B	9328.40		1.09		0.79	
9A	43152.40	43163.45	1.88	1.87	2.10	2.09
9B	43174.50		1.87		2.08	
10A	9175.10	9090.00	1.77	1.77	1.56	1.55
10B	9004.90		1.77		1.55	
11A	4192.60	4194.85	1.68	1.68	1.23	1.29
11B	4197.10		1.67		1.34	
12A	2669.80	2742.30	1.52	1.48	1.08	1.05
12B	2814.80		1.43		1.02	
13A	8699.70	10587.50	0.44	0.42	0.42	0.40
13B	12475.30		0.40		0.38	

Method → Sample ↓	Phenol-Chloroform Nucleic Acid [ng in extract]	Mean [ng in extract]	A260/A280	Mean	A260/A230	Mean
1A	311.90	305.05	1.77	1.69	0.43	0.32
1B	298.20		1.61		0.21	
2A	17746.90	16983.90	1.88	1.86	1.24	1.20
2B	16220.90		1.83		1.17	
3A	16530.40	16721.30	1.84	1.86	1.59	1.72
3B	16912.20		1.87		1.85	
4A	1280.50	981.00	1.75	1.72	0.64	0.69
4B	681.50		1.69		0.73	
5A	393.60	460.05	1.80	1.79	0.36	0.36
5B	526.50		1.77		0.35	
6A	20846.90	21669.60	1.96	1.96	1.99	2.00
6B	22492.30		1.97		2.02	
7A	3285.30	3145.95	2.07	2.02	0.88	0.95
7B	3006.60		1.97		1.02	
8A	7876.70	7721.15	1.76	1.79	0.95	1.03
8B	7565.60		1.82		1.11	
9A	34125.70	24742.90	1.77	1.60	2.06	1.70
9B	15360.10		1.43		1.34	
10A	4784.80	5607.80	1.87	1.86	1.28	1.39
10B	6430.80		1.85		1.49	
11A	1213.90	1252.40	1.62	1.70	0.41	0.51
11B	1290.90		1.77		0.60	
12A	3124.70	2425.15	0.39	0.39	0.29	0.29
12B	1725.60		0.40		0.29	
13A	829.80	883.75	0.38	0.38	0.25	0.27
13B	937.70		0.37		0.28	

Table S16: Data for Fig. 4A. Total DNA content of extracts from clinical urines. Extract from 1 ml of urine. Measured with Qubit.

Method →	IL-DEx		Method →	QIAGEN		Method →	Promega		Method →	Phenol-Chloroform	
**Sample ↓**	**DNA [ng in extract]**	**Mean [ng in extract]**	**Sample ↓**	**DNA [ng in extract]**	**Mean [ng in extract]**	**Sample ↓**	**DNA [ng in extract]**	**Mean [ng in extract]**	**Sample ↓**	**DNA [ng in extract]**	**Mean [ng in extract]**
1A	146.00	102.20	1A	133.00	79.10	1A	580.00	341.50	1A	289.00	163.15
1B	58.40		1B	25.20		1B	103.00		1B	37.30	
2A	609.00	335.95	2A	1420.00	793.50	2A	85000.00	50000.00	2A	29600.00	16280.00
2B	62.90		2B	167.00		2B	15000.00		2B	2960.00	
3A	2230.00	1326.00	3A	596.00	378.50	3A	33600.00	19150.00	3A	45400.00	26580.00
3B	422.00		3B	161.00		3B	4700.00		3B	7760.00	
4A	311.00	192.15	4A	331.00	198.40	4A	2780.00	1651.50	4A	2370.00	1324.00
4B	73.30		4B	65.80		4B	523.00		4B	278.00	
5A	1310.00	747.50	5A	115.00	79.95	5A	2080.00	1228.00	5A	332.00	208.60
5B	185.00		5B	44.90		5B	376.00		5B	85.20	
6A	3670.00	2078.00	6A	308.00	220.00	6A	22400.00	13080.00	6A	49800.00	29000.00
6B	486.00		6B	132.00		6B	3760.00		6B	8200.00	
7A	3250.00	1884.50	7A	622.00	415.50	7A	23600.00	13510.00	7A	6590.00	3592.50
7B	519.00		7B	209.00		7B	3420.00		7B	595.00	
8A	3020.00	1527.55	8A	2420.00	1518.00	8A	3920.00	3900.00	8A	610.00	548.50
8B	35.10		8B	616.00		8B	3880.00		8B	487.00	
9A	1640.00	1023.50	9A	1020.00	709.50	9A	212000.00	123200.00	9A	135000.00	69790.00
9B	407.00		9B	399.00		9B	34400.00		9B	4580.00	
10A	314.00	190.85	10A	690.00	443.00	10A	40600.00	23160.00	10A	18200.00	10755.00
10B	67.70		10B	196.00		10B	5720.00		10B	3310.00	
11A	137.00	73.95	11A	31.00	18.90	11A	7860.00	4573.00	11A	2870.00	1686.00
11B	10.90		11B	6.80		11B	1286.00		11B	502.00	
12A	26.00	26.00	12A	388.00	388.00	12A	114.00	69.60	12A	75.00	40.95
12B	n.d.		12B	n.d.		12B	25.20		12B	6.90	
13A	32.00	19.05	13A	10.00	10.00	13A	117.00	71.75	13A	28.00	28.00
13B	6.10		13B	n.d.		13B	26.50		13B	n.d.	

Table S17: Data for Fig. 4B. 16S rRNA gene content of extracts from clinical urines. Extract from 1 ml of urine. The 16S background was calculated from the mean of the extraction control + 3x standard deviation and is the limit above which values are true positives with a probability of 95%. Values that fall under the limit (grey) were not included in the diagram.

Method →	16S rRNA gene content [copies in extract]				Method →	Mean [copies in extract]				SD [copies in extract]				16S Background = Mean of Extraction control + 3xStandard deviation			
Sample ↓	IL-DEx	QIAGEN	Promega	Phenol-Chloroform	Sample ↓	IL-DEx	QIAGEN	Promega	Phenol-Chloroform	IL-DEx	QIAGEN	Promega	Phenol-Chloroform	IL-DEx	QIAGEN	Promega	Phenol-Chloroform
UTI 1A	6.13E+08	3.70E+08	1.55E+08	2.19E+07	**UTI 1**	6.65E+08	3.56E+08	1.63E+08	2.64E+07	7.63E+07	2.01E+07	1.17E+07	7.03E+06				
UTI 1B	7.21E+08	3.42E+08	1.71E+08	3.18E+07													
UTI 2A	4.45E+06	5.56E+08	1.38E+10	3.67E+09	**UTI 2**	4.30E+06	4.96E+08	1.27E+10	4.04E+09	2.12E+05	8.07E+07	1.51E+09	5.40E+08				
UTI 2B	4.15E+06	4.42E+08	1.16E+10	4.44E+09													
UTI 3A	3.32E+08	1.81E+08	6.32E+09	5.67E+09	**UTI 3**	4.07E+08	1.78E+08	5.98E+09	5.64E+09	1.19E+08	5.24E+06	4.74E+08	5.38E+07				
UTI 23	5.00E+08	1.74E+08	5.65E+09	5.60E+09													
UTI 4A	9.80E+06	8.18E+07	2.53E+08	5.55E+07	**UTI 4**	7.36E+06	8.03E+07	2.58E+08	4.58E+07	3.03E+06	2.17E+06	8.32E+06	1.25E+07				
UTI 4B	5.52E+06	7.87E+07	2.64E+08	3.78E+07													
UTI 5A	2.63E+09	7.19E+07	2.28E+09	1.02E+08	**UTI 5**	2.62E+09	1.59E+08	2.31E+09	1.94E+08	1.31E+07	1.98E+08	4.59E+07	1.90E+08				
UTI 5B	2.61E+09	3.51E+08	2.35E+09	3.71E+08													
UTI 6A	2.97E+09	8.74E+07	1.45E+10	9.38E+09	**UTI 6**	2.28E+09	1.25E+08	1.38E+10	9.49E+09	8.72E+08	6.43E+07	9.91E+08	1.59E+08				
UTI 6B	1.74E+09	1.78E+08	1.31E+10	9.61E+09													
UTI 7A	4.00E+08	4.30E+08	1.03E+10	2.08E+09	**UTI 7**	3.66E+08	6.33E+08	1.03E+10	2.91E+09	4.66E+07	3.54E+08	2.56E+07	1.42E+09				
UTI 7B	3.34E+08	9.31E+08	1.04E+10	4.08E+09													
UTI 8A	8.07E+08	1.30E+09	3.75E+09	1.36E+09	**UTI 8**	1.47E+09	1.67E+09	4.14E+09	1.02E+09	1.33E+09	5.84E+08	5.75E+08	4.18E+08				
UTI 8B	2.69E+09	2.13E+09	4.56E+09	7.68E+08													
UTI 9A	1.22E+07	7.47E+07	2.99E+09	1.36E+09	**UTI 9**	1.49E+07	9.13E+07	2.92E+09	7.10E+08	4.10E+06	2.60E+07	1.01E+08	6.99E+08				
UTI 9B	1.80E+07	1.11E+08	2.84E+09	3.71E+08													
UTI 10A	3.56E+05	5.95E+05	8.09E+06	1.92E+06	**UTI 10**	4.26E+05	7.62E+05	7.12E+06	2.98E+06	1.08E+05	2.70E+05	1.29E+06	1.93E+06				
UTI 10B	5.09E+05	9.76E+05	6.27E+06	4.64E+06													
non-UTI 11A	1.20E+06	3.72E+06	8.08E+05	8.38E+07	**non-UTI 11**	7.29E+05	3.58E+06	8.73E+05	8.65E+07	5.39E+05	1.94E+05	9.66E+04	3.92E+06				
non-UTI 11B	4.41E+05	3.44E+06	9.44E+05	8.93E+07													
non-UTI 12A	1.24E+06	4.44E+07	1.85E+06	2.57E+05	**non-UTI 12**	1.24E+06	4.28E+07	1.90E+06	3.23E+05	8.74E+03	2.31E+06	6.71E+04	1.06E+05				
non-UTI 12B	1.23E+06	4.12E+07	1.95E+06	4.06E+05													
non-UTI 13A	2.29E+04	3.20E+04	2.06E+04	8.44E+04	**non-UTI 13**	2.71E+04	6.61E+04	1.13E+04	8.45E+04	6.47E+03	7.41E+04	1.02E+04	8.53E+01				
non-UTI 13B	3.21E+04	1.37E+05	6.20E+03	8.45E+04													
Extraction control A	1.12E+05	1.33E+04	5.73E+02	2.46E+04	**Extraction control**	8.12E+04	1.05E+04	4.88E+02	2.95E+04	3.75E+04	3.60E+03	1.12E+02	7.57E+03	1.94E+05	2.13E+04	8.23E+02	5.22E+04
Extraction control B	5.89E+04	8.26E+03	4.15E+02	3.53E+04													

Method →	16S rRNA gene content [logcopies in extract]				Method →	Mean [logcopies in extract]				SD [logcopies in extract]				16S Background = Mean of Extraction control + 3xStandard deviation			
Sample ↓	IL-DEx	QIAGEN	Promega	Phenol-Chloroform	Sample ↓	IL-DEx	QIAGEN	Promega	Phenol-Chloroform	IL-DEx	QIAGEN	Promega	Phenol-Chloroform	IL-DEx	QIAGEN	Promega	Phenol-Chloroform
UTI 1A	8.79	8.57	8.19	7.34	**UTI 1**	8.82	8.55	8.21	7.42	0.05	0.02	0.03	0.12				
UTI 1B	8.86	8.53	8.23	7.50													
UTI 2A	6.65	8.75	10.14	9.56	**UTI 2**	6.63	8.70	10.10	9.61	0.02	0.07	0.05	0.06				
UTI 2B	6.62	8.65	10.07	9.65													
UTI 3A	8.52	8.26	9.80	9.75	**UTI 3**	8.61	8.25	9.78	9.75	0.13	0.01	0.03	0.00				
UTI 23	8.70	8.24	9.75	9.75													
UTI 4A	6.99	7.91	8.40	7.74	**UTI 4**	6.87	7.90	8.41	7.66	0.18	0.01	0.01	0.12				
UTI 4B	6.74	7.90	8.42	7.58													
UTI 5A	9.42	7.86	9.36	8.01	**UTI 5**	9.42	8.20	9.36	8.29	0.00	0.49	0.01	0.40				
UTI 5B	9.42	8.55	9.37	8.57													
UTI 6A	9.47	7.94	10.16	9.97	**UTI 6**	9.36	8.10	10.14	9.98	0.16	0.22	0.03	0.01				
UTI 6B	9.24	8.25	10.12	9.98													
UTI 7A	8.60	8.63	10.01	9.32	**UTI 7**	8.56	8.80	10.01	9.46	0.06	0.24	0.00	0.21				
UTI 7B	8.52	8.97	10.02	9.61													
UTI 8A	8.91	9.12	9.57	9.13	**UTI 8**	9.17	9.22	9.62	9.01	0.37	0.15	0.06	0.18				
UTI 8B	9.43	9.33	9.66	8.89													
UTI 9A	7.09	7.87	9.48	9.13	**UTI 9**	7.17	7.96	9.46	8.85	0.12	0.12	0.02	0.40				
UTI 9B	7.26	8.05	9.45	8.57													
UTI 10A	5.55	5.77	6.91	6.28	**UTI 10**	5.63	5.88	6.85	6.47	0.11	0.15	0.08	0.27				
UTI 10B	5.71	5.99	6.80	6.67													
non-UTI 11A	6.08	6.57	5.91	7.92	**non-UTI 11**	5.86	6.55	5.94	7.94	0.31	0.02	0.05	0.02				
non-UTI 11B	5.64	6.54	5.98	7.95													
non-UTI 12A	6.10	7.65	6.27	5.41	**non-UTI 12**	6.09	7.63	6.28	5.51	0.00	0.02	0.02	0.14				
non-UTI 12B	6.09	7.61	6.29	5.61													
non-UTI 13A	4.36	4.50	4.31	4.93	**non-UTI 13**	4.43	4.82	4.05	4.93	0.10	0.45	0.37	0.00				
non-UTI 13B	4.51	5.14	3.79	4.93													
Extraction control A	5.05	4.13	2.76	4.39	**Extraction control**	4.91	4.02	2.69	4.47	0.20	0.15	0.10	0.11	5.29	4.33	2.92	4.72
Extraction control B	4.77	3.92	2.62	4.55													

Table S18: Data for Fig. 4C. E. coli 23S rRNA gene content of extracts from clinical urines. Extract from 1 ml of urine. Measured with E.coli-qPCR. The background was calculated from the mean of the extraction control + 3x standard deviation and is the limit above which values are true positives with a probability of 95%. Values that fall under the limit (grey) were not included in the diagram.

Method → Sample ↓	23S rRNA gene content [copies in extract]			
	IL-DEx	QIAGEN	Promega	Phenol-Chloroform
UTI 1A	n.d.	3.01E+04	1.83E+03	1.14E+03
UTI 1B	n.d.	4.70E+03	n.d.	8.76E+02
UTI 2A	7.80E+06	3.56E+08	1.15E+10	3.36E+09
UTI 2B	6.19E+06	2.20E+08	7.60E+09	5.73E+09
UTI 3A	1.10E+08	8.60E+07	2.45E+09	3.92E+09
UTI 23	2.08E+08	6.73E+07	2.70E+09	3.54E+09
UTI 4A	1.83E+06	4.22E+07	2.68E+08	3.40E+07
UTI 4B	2.74E+06	5.14E+07	9.66E+07	2.12E+07
UTI 5A	1.21E+09	7.34E+07	1.18E+09	3.09E+07
UTI 5B	7.04E+08	2.31E+08	1.10E+09	1.03E+08
UTI 6A	7.68E+08	3.32E+07	4.21E+09	9.94E+09
UTI 6B	5.61E+08	1.17E+08	8.85E+09	6.77E+09
UTI 7A	1.36E+03	5.64E+04	6.68E+03	2.21E+03
UTI 7B	1.26E+03	2.98E+04	1.34E+04	2.31E+03
UTI 8A	4.61E+08	3.66E+08	2.47E+09	6.19E+08
UTI 8B	7.63E+08	6.25E+08	1.81E+09	3.72E+08
UTI 9A	1.95E+07	1.02E+08	2.03E+09	1.40E+09
UTI 9B	2.14E+07	8.19E+07	4.12E+09	2.94E+08
UTI 10A	9.21E+02	4.36E+04	7.26E+03	1.93E+03
UTI 10B	8.90E+02	1.34E+05	5.52E+04	1.92E+02
non-UTI 11A	5.08E+02	2.46E+03	3.54E+02	n.d.
non-UTI 11B	3.77E+02	2.15E+03	3.90E+02	4.50E+02
non-UTI 12A	9.10E+02	7.52E+03	1.85E+02	6.14E+02
non-UTI 12B	5.11E+02	1.23E+03	2.78E+02	2.88E+02
non-UTI 13A	3.35E+02	6.84E+03	3.35E+02	n.d.
non-UTI 13B	6.79E+02	2.44E+03	5.15E+02	2.04E+02
Extraction control A	6.27E+02	1.06E+03	n.d.	n.d.
Extraction control B	2.59E+02	2.38E+03	4.95E+02	7.67E+02

Method → Sample ↓	Mean [copies in extract]				SD [copies in extract]				23S Background = Mean of Extraction control + 3xStandard deviation			
	IL-DEx	QIAGEN	Promega	Phenol-Chloroform	IL-DEx	QIAGEN	Promega	Phenol-Chloroform	IL-DEx	QIAGEN	Promega	Phenol-Chloroform
**UTI 1**	n.d.	1.19E+04	1.83E+03	9.97E+02		1.80E+04		1.83E+02				
**UTI 2**	6.94E+06	2.80E+08	9.37E+09	4.39E+09	1.14E+06	9.62E+07	2.79E+09	1.67E+09				
**UTI 3**	1.52E+08	7.61E+07	2.57E+09	3.73E+09	6.93E+07	1.32E+07	1.70E+08	2.70E+08				
**UTI 4**	2.24E+06	4.66E+07	1.61E+08	2.69E+07	6.43E+05	6.50E+06	1.21E+08	9.01E+06				
**UTI 5**	9.23E+08	1.30E+08	1.14E+09	5.63E+07	3.57E+08	1.11E+08	5.37E+07	5.07E+07				
**UTI 6**	6.57E+08	6.23E+07	6.10E+09	8.20E+09	1.46E+08	5.91E+07	3.28E+09	2.24E+09				
**UTI 7**	1.31E+03	4.10E+04	9.47E+03	2.26E+03	7.08E+01	1.88E+04	4.79E+03	6.91E+01				
**UTI 8**	5.93E+08	4.78E+08	2.11E+09	4.80E+08	2.13E+08	1.83E+08	4.65E+08	1.74E+08				
**UTI 9**	2.04E+07	9.13E+07	2.89E+09	6.41E+08	1.38E+06	1.41E+07	1.48E+09	7.80E+08				
**UTI 10**	9.05E+02	7.63E+04	2.00E+04	6.10E+02	2.22E+01	6.37E+04	3.39E+04	1.23E+03				
**non-UTI 11**	4.38E+02	2.30E+03	3.72E+02	4.50E+02	9.27E+01	2.21E+02	2.55E+01					
**non-UTI 12**	6.82E+02	3.05E+03	2.27E+02	4.20E+02	2.82E+02	4.44E+03	6.59E+01	2.31E+02				
**non-UTI 13**	4.77E+02	4.09E+03	4.15E+02	2.04E+02	2.43E+02	3.11E+03	1.27E+02					
**Extraction control**	4.03E+02	1.59E+03	4.95E+02	7.67E+02	2.60E+02	9.30E+02			1.18E+03	4.38E+03	4.95E+02	7.67E+02

Method → Sample ↓	23S rRNA gene content [logcopies in extract]			
	IL-DEx	QIAGEN	Promega	Phenol-Chloroform
UTI 1A	n.d.	4.48	3.26	3.06
UTI 1B	n.d.	3.67	n..d	2.94
UTI 2A	6.89	8.55	10.06	9.53
UTI 2B	6.79	8.34	9.88	9.76
UTI 3A	8.04	7.93	9.39	9.59
UTI 23	8.32	7.83	9.43	9.55
UTI 4A	6.26	7.63	8.43	7.53
UTI 4B	6.44	7.71	7.99	7.33
UTI 5A	9.08	7.87	9.07	7.49
UTI 5B	8.85	8.36	9.04	8.01
UTI 6A	8.89	7.52	9.62	10.00
UTI 6B	8.75	8.07	9.95	9.83
UTI 7A	3.13	4.75	3.82	3.34
UTI 7B	3.10	4.47	4.13	3.36
UTI 8A	8.66	8.56	9.39	8.79
UTI 8B	8.88	8.80	9.26	8.57
UTI 9A	7.29	8.01	9.31	9.15
UTI 9B	7.33	7.91	9.61	8.47
UTI 10A	2.96	4.64	3.86	3.29
UTI 10B	2.95	5.13	4.74	2.28
non-UTI 11A	2.71	3.39	2.55	n.d.
non-UTI 11B	2.58	3.33	2.59	2.65
non-UTI 12A	2.96	3.88	2.27	2.79
non-UTI 12B	2.71	3.09	2.44	2.46
non-UTI 13A	2.52	3.84	2.53	n.d.
non-UTI 13B	2.83	3.39	2.71	2.31
Extraction control A	2.80	3.03	n.d.	n.d.
Extraction control B	2.41	3.38	2.69	2.88

Method → Sample ↓	Mean [logcopies in extract]				SD [logcopies in extract]				16S Background = Mean of Extraction control + 3xStandard deviation			
	IL-DEx	QIAGEN	Promega	Phenol-Chloroform	IL-DEx	QIAGEN	Promega	Phenol-Chloroform	IL-DEx	QIAGEN	Promega	Phenol-Chloroform
**UTI 1**	n.d.	4.08	3.26	3.00		0.57		0.08				
**UTI 2**	6.84	8.45	9.97	9.64	0.07	0.15	0.13	0.16				
**UTI 3**	8.18	7.88	9.41	9.57	0.20	0.08	0.03	0.03				
**UTI 4**	6.35	7.67	8.21	7.43	0.12	0.06	0.31	0.14				
**UTI 5**	8.97	8.11	9.06	7.75	0.17	0.35	0.02	0.37				
**UTI 6**	8.82	7.79	9.79	9.91	0.10	0.39	0.23	0.12				
**UTI 7**	3.12	4.61	3.98	3.35	0.02	0.20	0.21	0.01				
**UTI 8**	8.77	8.68	9.32	8.68	0.15	0.16	0.10	0.16				
**UTI 9**	7.31	7.96	9.46	8.81	0.03	0.07	0.22	0.48				
**UTI 10**	2.96	4.88	4.30	2.79	0.01	0.34	0.62	0.71				
**non-UTI 11**	2.64	3.36	2.57	2.65	0.09	0.04	0.03					
**non-UTI 12**	2.83	3.48	2.36	2.62	0.18	0.55	0.13	0.23				
**non-UTI 13**	2.68	3.61	2.62	2.31	0.22	0.32	0.13					
**Extraction control**	2.60	3.20	2.69	2.88	0.27	0.25			3.07	3.64	2.69	2.88

Table S19: Data for Fig. S9. Human DNA content of extracts from clinical urines. Extract from 1 ml of urine. Measured with human-qPCR. The Background was calculated from the mean of the extraction control + 3x standard deviation and is the limit above which values are true positives with a probability of 95%.

Method →	human DNA content [ng in extract]			
Sample ↓	IL-DEx	QIAGEN	Promega	Phenol-Chloroform
UTI 1A	1035.05	1501.90	1588.94	286.68
UTI 1B	1131.44	1390.89	1695.18	858.86
UTI 2A	565.27	3183.28	192269.38	48093.75
UTI 2B	529.97	1903.88	171125.90	37344.55
UTI 3A	3887.78	538.99	51014.83	119183.52
UTI 23	6257.42	1067.42	52863.50	89099.58
UTI 4A	1515.65	3021.00	8196.83	5030.09
UTI 4B	1978.47	3712.89	11111.01	3581.08
UTI 5A	4894.57	134.99	5193.79	222.85
UTI 5B	863.54	901.50	4432.21	382.26
UTI 6A	7616.23	422.65	37879.11	116574.44
UTI 6B	7385.92	676.86	25195.30	121850.99
UTI 7A	10789.14	935.66	31194.34	7277.19
UTI 7B	12296.09	2911.45	25107.52	9254.05
UTI 8A	15151.15	9581.32	33003.31	15012.11
UTI 8B	20282.85	11453.97	53552.22	11635.55
UTI 9A	2169.38	1812.39	219549.01	84114.77
UTI 9B	4262.92	3912.34	263168.46	70464.33
UTI 10A	547.74	1880.59	26761.66	36272.99
UTI 10B	541.49	1683.34	104639.36	59403.62
non-UTI 11A	84.55	753.85	24079.30	5744.51
non-UTI 11B	38.85	455.05	18707.80	5654.41
non-UTI 12A	123.26	2376.21	652.57	31.44
non-UTI 12B	89.57	3039.65	693.95	22.34
non-UTI 13A	83.20	0.56	413.49	59.55
non-UTI 13B	38.85	0.70	490.85	61.08
Extraction control A	0.04	0.12	0.02	0.03
Extraction control B	0.05	0.08	0.03	0.03

Method →	Mean [ng in extract]				SD [ng in extract]				Background = Mean of Extraction control + 3xStandard deviation			
Sample ↓	IL-DEx	QIAGEN	Promega	Phenol-Chloroform	IL-DEx	QIAGEN	Promega	Phenol-Chloroform	IL-DEx	QIAGEN	Promega	Phenol-Chloroform
**UTI 1**	1082.17	1445.33	1641.20	496.20	68.16	78.49	75.12	404.59				
**UTI 2**	547.34	2461.83	181389.83	42379.70	24.96	904.67	14950.69	7600.83				
**UTI 3**	4932.29	758.50	51930.94	103049.51	1675.59	373.66	1307.20	21272.56				
**UTI 4**	1731.67	3349.12	9543.33	4244.19	327.26	489.24	2060.64	1024.61				
**UTI 5**	2055.88	348.84	4797.91	291.87	2850.37	542.01	538.52	112.73				
**UTI 6**	7500.19	534.86	30892.97	119183.52	162.85	179.75	8968.81	3731.09				
**UTI 7**	11518.00	1650.50	27985.94	8206.31	1065.57	1397.09	4304.03	1397.85				
**UTI 8**	17530.22	10475.89	42040.46	13216.43	3628.66	1324.16	14530.27	2387.59				
**UTI 9**	3041.03	2662.84	240371.33	76987.61	1480.36	1484.88	30843.61	9652.32				
**UTI 10**	544.60	1779.23	52918.08	46419.25	4.42	139.48	55067.85	16355.83				
**non-UTI 11**	57.31	585.69	21224.30	5699.28	32.31	211.28	3798.22	63.72				
**non-UTI 12**	105.07	2687.54	672.94	26.50	23.83	469.12	29.26	6.43				
**non-UTI 13**	56.86	0.63	450.51	60.31	31.36	0.10	54.70	1.08				
**Extraction control**	0.04	0.10	0.02	0.03	0.01	0.03	0.01	0.002	0.07	0.17	0.06	0.04

Table S20: Data for Fig. 5. 16S rRNA gene sequencing results for extracts from clinical urines.

Sample ID	Sample Type	tax_id	abundance	estimated counts	species	genus	family	order	class	phylum	clade
IL-DEx_1A_UTI	UTI	545	93.37%	17 838.40	Citrobacter koseri	Citrobacter	Enterobacteriaceae	Enterobacterales	Gammaproteobacteria	Proteobacteria	
IL-DEx_1A_UTI	UTI	1852374	1.79%	341.71	Ezakiella massiliensis	Ezakiella			Tissierellia	Firmicutes	Terrabacteria group
IL-DEx_1A_UTI	UTI	386414	1.52%	290.41	Prevotella timonensis	Prevotella	Prevotellaceae	Bacteroidales	Bacteroidia	Bacteroidetes	FCB group
IL-DEx_1A_UTI	UTI	827	0.91%	173.28	Campylobacter ureolyticus	Campylobacter	Campylobacteraceae	Campylobacterales	Epsilonproteobacteria	Proteobacteria	
IL-DEx_1A_UTI	UTI	1702287	0.42%	80.35	Negativibacillus massiliensis	Negativicoccus	Veillonellaceae	Veillonellales	Negativicutes	Firmicutes	Terrabacteria group
IL-DEx_1A_UTI	UTI	1376	0.31%	60.02	Aerococcus urinae	Aerococcus	Aerococcaceae	Lactobacillales	Bacilli	Firmicutes	Terrabacteria group
IL-DEx_1A_UTI	UTI	322095	0.27%	51.31	Porphyromonas somerae	Porphyromonas	Porphyromonadaceae	Bacteroidales	Bacteroidia	Bacteroidetes	FCB group
IL-DEx_1A_UTI	UTI	371674	0.24%	45.50	Moryella indoligenes	Moryella	Lachnospiraceae	Clostridiales	Clostridia	Firmicutes	Terrabacteria group
IL-DEx_1A_UTI	UTI	507750	0.17%	31.94	Peptoniphilus duerdenii	Peptoniphilus	Peptoniphilaceae	Tissierellales	Tissierellia	Firmicutes	Terrabacteria group
IL-DEx_1A_UTI	UTI	266762	0.16%	30.01	Porphyromonas gingivicanis	Porphyromonas	Porphyromonadaceae	Bacteroidales	Bacteroidia	Bacteroidetes	FCB group
IL-DEx_1A_UTI	UTI	884684	0.15%	29.04	Mageeibacillus indolicus	Mageeibacillus	Hungateiclostridiaceae	Clostridiales	Clostridia	Firmicutes	Terrabacteria group
IL-DEx_1A_UTI	UTI	33031	0.13%	24.20	Peptoniphilus lacrimalis	Peptoniphilus	Peptoniphilaceae	Tissierellales	Tissierellia	Firmicutes	Terrabacteria group
IL-DEx_1A_UTI	UTI	501496	0.12%	23.23	Porphyromonas bennonis	Porphyromonas	Porphyromonadaceae	Bacteroidales	Bacteroidia	Bacteroidetes	FCB group
IL-DEx_1A_UTI	UTI	46507	0.11%	20.33	Ezakiella coagulans	Ezakiella			Tissierellia	Firmicutes	Terrabacteria group
IL-DEx_1A_UTI	UTI	2576406	0.10%	18.81	Citrobacter sp. TBCP-5362	Citrobacter	Enterobacteriaceae	Enterobacterales	Gammaproteobacteria	Proteobacteria	
IL-DEx_1A_UTI	UTI	2490633	0.09%	17.42	Streptococcus periodonticum	Streptococcus	Streptococcaceae	Lactobacillales	Bacilli	Firmicutes	Terrabacteria group
IL-DEx_1A_UTI	UTI	33012	0.08%	15.49	Propionimicrobium lymphophilum	Propionimicrobium	Propionibacteriaceae	Propionibacteriales	Actinobacteria	Actinobacteria	Terrabacteria group
IL-DEx_1A_UTI	UTI	309120	0.07%	13.55	Dialister micraerophilus	Dialister	Veillonellaceae	Veillonellales	Negativicutes	Firmicutes	Terrabacteria group
IL-DEx_1A_UTI	UTI	unmapped	0.00%	3.00							
IL-DEx_1A_UTI	UTI	mapped_unclassified	0.00%	682.00							
QIAGEN_1A_UTI	UTI	545	89.82%	16 620.95	Citrobacter koseri	Citrobacter	Enterobacteriaceae	Enterobacterales	Gammaproteobacteria	Proteobacteria	
QIAGEN_1A_UTI	UTI	1852374	2.21%	409.20	Ezakiella massiliensis	Ezakiella			Tissierellia	Firmicutes	Terrabacteria group
QIAGEN_1A_UTI	UTI	386414	1.67%	309.48	Prevotella timonensis	Prevotella	Prevotellaceae	Bacteroidales	Bacteroidia	Bacteroidetes	FCB group
QIAGEN_1A_UTI	UTI	33012	0.85%	157.09	Propionimicrobium lymphophilum	Propionimicrobium	Propionibacteriaceae	Propionibacteriales	Actinobacteria	Actinobacteria	Terrabacteria group
QIAGEN_1A_UTI	UTI	827	0.79%	146.75	Campylobacter ureolyticus	Campylobacter	Campylobacteraceae	Campylobacterales	Epsilonproteobacteria	Proteobacteria	
QIAGEN_1A_UTI	UTI	1702287	0.64%	117.58	Negativibacillus massiliensis	Negativicoccus	Veillonellaceae	Veillonellales	Negativicutes	Firmicutes	Terrabacteria group
QIAGEN_1A_UTI	UTI	1376	0.54%	99.71	Aerococcus urinae	Aerococcus	Aerococcaceae	Lactobacillales	Bacilli	Firmicutes	Terrabacteria group
QIAGEN_1A_UTI	UTI	371674	0.51%	94.71	Moryella indoligenes	Moryella	Lachnospiraceae	Clostridiales	Clostridia	Firmicutes	Terrabacteria group
QIAGEN_1A_UTI	UTI	2490633	0.48%	88.78	Streptococcus periodonticum	Streptococcus	Streptococcaceae	Lactobacillales	Bacilli	Firmicutes	Terrabacteria group
QIAGEN_1A_UTI	UTI	322095	0.36%	65.85	Porphyromonas somerae	Porphyromonas	Porphyromonadaceae	Bacteroidales	Bacteroidia	Bacteroidetes	FCB group
QIAGEN_1A_UTI	UTI	507750	0.29%	53.62	Peptoniphilus duerdenii	Peptoniphilus	Peptoniphilaceae	Tissierellales	Tissierellia	Firmicutes	Terrabacteria group
QIAGEN_1A_UTI	UTI	884684	0.24%	44.21	Mageeibacillus indolicus	Mageeibacillus	Hungateiclostridiaceae	Clostridiales	Clostridia	Firmicutes	Terrabacteria group
QIAGEN_1A_UTI	UTI	33031	0.23%	42.33	Peptoniphilus lacrimalis	Peptoniphilus	Peptoniphilaceae	Tissierellales	Tissierellia	Firmicutes	Terrabacteria group
QIAGEN_1A_UTI	UTI	190146	0.18%	33.86	Actinotignum urinale	Actinotignum	Actinomycetaceae	Actinomycetales	Actinobacteria	Actinobacteria	Terrabacteria group
QIAGEN_1A_UTI	UTI	266762	0.18%	33.86	Porphyromonas gingivicanis	Porphyromonas	Porphyromonadaceae	Bacteroidales	Bacteroidia	Bacteroidetes	FCB group
QIAGEN_1A_UTI	UTI	46507	0.18%	33.86	Ezakiella coagulans	Ezakiella			Tissierellia	Firmicutes	Terrabacteria group
QIAGEN_1A_UTI	UTI	54005	0.16%	30.10	Peptoniphilus harei	Peptoniphilus	Peptoniphilaceae	Tissierellales	Tissierellia	Firmicutes	Terrabacteria group
QIAGEN_1A_UTI	UTI	501496	0.12%	22.58	Porphyromonas bennonis	Porphyromonas	Porphyromonadaceae	Bacteroidales	Bacteroidia	Bacteroidetes	FCB group
QIAGEN_1A_UTI	UTI	2576406	0.11%	21.07	Citrobacter sp. TBCP-5362	Citrobacter	Enterobacteriaceae	Enterobacterales	Gammaproteobacteria	Proteobacteria	
QIAGEN_1A_UTI	UTI	796942	0.09%	16.29	Stomatobaculum longum	Stomatobaculum	Lachnospiraceae	Clostridiales	Clostridia	Firmicutes	Terrabacteria group
QIAGEN_1A_UTI	UTI	1328	0.08%	15.64	Streptococcus anginosus	Streptococcus	Streptococcaceae	Lactobacillales	Bacilli	Firmicutes	Terrabacteria group
QIAGEN_1A_UTI	UTI	562	0.08%	14.16	Escherichia coli	Escherichia	Enterobacteriaceae	Enterobacterales	Gammaproteobacteria	Proteobacteria	
QIAGEN_1A_UTI	UTI	28901	0.07%	12.61	Salmonella enterica	Salmonella	Enterobacteriaceae	Enterobacterales	Gammaproteobacteria	Proteobacteria	
QIAGEN_1A_UTI	UTI	28127	0.06%	11.29	Prevotella buccalis	Prevotella	Prevotellaceae	Bacteroidales	Bacteroidia	Bacteroidetes	FCB group
QIAGEN_1A_UTI	UTI	1871034	0.05%	9.41	Propionimicrobium sp. Marseille-P3275	Propionimicrobium	Propionibacteriaceae	Propionibacteriales	Actinobacteria	Actinobacteria	Terrabacteria group
QIAGEN_1A_UTI	UTI	unmapped	0.00%	5.00							
QIAGEN_1A_UTI	UTI	mapped_unclassified	0.00%	1 235.00							
Promega_1A_UTI	UTI	545	60.10%	11 076.99	Citrobacter koseri	Citrobacter	Enterobacteriaceae	Enterobacterales	Gammaproteobacteria	Proteobacteria	
Promega_1A_UTI	UTI	1852374	12.87%	2 371.78	Ezakiella massiliensis	Ezakiella			Tissierellia	Firmicutes	Terrabacteria group
Promega_1A_UTI	UTI	386414	6.39%	1 177.97	Prevotella timonensis	Prevotella	Prevotellaceae	Bacteroidales	Bacteroidia	Bacteroidetes	FCB group
Promega_1A_UTI	UTI	827	4.01%	739.96	Campylobacter ureolyticus	Campylobacter	Campylobacteraceae	Campylobacterales	Epsilonproteobacteria	Proteobacteria	
Promega_1A_UTI	UTI	1702287	3.91%	721.32	Negativibacillus massiliensis	Negativicoccus	Veillonellaceae	Veillonellales	Negativicutes	Firmicutes	Terrabacteria group
Promega_1A_UTI	UTI	1376	2.88%	531.20	Aerococcus urinae	Aerococcus	Aerococcaceae	Lactobacillales	Bacilli	Firmicutes	Terrabacteria group
Promega_1A_UTI	UTI	884684	2.86%	526.30	Mageeibacillus indolicus	Mageeibacillus	Hungateiclostridiaceae	Clostridiales	Clostridia	Firmicutes	Terrabacteria group
Promega_1A_UTI	UTI	322095	0.96%	177.09	Porphyromonas somerae	Porphyromonas	Porphyromonadaceae	Bacteroidales	Bacteroidia	Bacteroidetes	FCB group
Promega_1A_UTI	UTI	33012	0.96%	176.14	Propionimicrobium lymphophilum	Propionimicrobium	Propionibacteriaceae	Propionibacteriales	Actinobacteria	Actinobacteria	Terrabacteria group
Promega_1A_UTI	UTI	371674	0.81%	150.11	Moryella indoligenes	Moryella	Lachnospiraceae	Clostridiales	Clostridia	Firmicutes	Terrabacteria group
Promega_1A_UTI	UTI	507750	0.76%	140.72	Peptoniphilus duerdenii	Peptoniphilus	Peptoniphilaceae	Tissierellales	Tissierellia	Firmicutes	Terrabacteria group
Promega_1A_UTI	UTI	2490633	0.58%	106.24	Streptococcus periodonticum	Streptococcus	Streptococcaceae	Lactobacillales	Bacilli	Firmicutes	Terrabacteria group
Promega_1A_UTI	UTI	266762	0.57%	105.83	Porphyromonas gingivicanis	Porphyromonas	Porphyromonadaceae	Bacteroidales	Bacteroidia	Bacteroidetes	FCB group
Promega_1A_UTI	UTI	501496	0.37%	68.01	Porphyromonas bennonis	Porphyromonas	Porphyromonadaceae	Bacteroidales	Bacteroidia	Bacteroidetes	FCB group
Promega_1A_UTI	UTI	33031	0.24%	43.80	Peptoniphilus lacrimalis	Peptoniphilus	Peptoniphilaceae	Tissierellales	Tissierellia	Firmicutes	Terrabacteria group
Promega_1A_UTI	UTI	190146	0.19%	35.41	Actinotignum urinale	Actinotignum	Actinomycetaceae	Actinomycetales	Actinobacteria	Actinobacteria	Terrabacteria group
Promega_1A_UTI	UTI	54005	0.18%	32.62	Peptoniphilus harei	Peptoniphilus	Peptoniphilaceae	Tissierellales	Tissierellia	Firmicutes	Terrabacteria group
Promega_1A_UTI	UTI	46507	0.18%	32.62	Ezakiella coagulans	Ezakiella			Tissierellia	Firmicutes	Terrabacteria group
Promega_1A_UTI	UTI	28127	0.16%	29.82	Prevotella buccalis	Prevotella	Prevotellaceae	Bacteroidales	Bacteroidia	Bacteroidetes	FCB group
Promega_1A_UTI	UTI	938289	0.15%	27.96	Levyella massiliensis	Levyella		Clostridiales	Clostridia	Firmicutes	Terrabacteria group
Promega_1A_UTI	UTI	309120	0.14%	25.16	Dialister micraerophilus	Dialister	Veillonellaceae	Veillonellales	Negativicutes	Firmicutes	Terrabacteria group
Promega_1A_UTI	UTI	319644	0.12%	22.61	Saccharofermentans acetigenes	Saccharofermentan	Hungateiclostridiaceae	Clostridiales	Clostridia	Firmicutes	Terrabacteria group
Promega_1A_UTI	UTI	562	0.10%	18.66	Escherichia coli	Escherichia	Enterobacteriaceae	Enterobacterales	Gammaproteobacteria	Proteobacteria	
Promega_1A_UTI	UTI	28123	0.09%	15.67	Porphyromonas asaccharolytica	Porphyromonas	Porphyromonadaceae	Bacteroidales	Bacteroidia	Bacteroidetes	FCB group
Promega_1A_UTI	UTI	1393034	0.08%	13.98	Atopobium deltae	Atopobium	Atopobiaceae	Coriobacteriales	Coriobacteriia	Actinobacteria	Terrabacteria group
Promega_1A_UTI	UTI	281920	0.07%	12.70	Porphyromonas uenonis	Porphyromonas	Porphyromonadaceae	Bacteroidales	Bacteroidia	Bacteroidetes	FCB group
Promega_1A_UTI	UTI	1871034	0.06%	11.18	Propionimicrobium sp. Marseille-P3275	Propionimicrobium	Propionibacteriaceae	Propionibacteriales	Actinobacteria	Actinobacteria	Terrabacteria group
Promega_1A_UTI	UTI	796942	0.06%	10.18	Stomatobaculum longum	Stomatobaculum	Lachnospiraceae	Clostridiales	Clostridia	Firmicutes	Terrabacteria group
Promega_1A_UTI	UTI	59505	0.05%	9.32	Actinotignum schaalii	Actinotignum	Actinomycetaceae	Actinomycetales	Actinobacteria	Actinobacteria	Terrabacteria group
Promega_1A_UTI	UTI	404403	0.05%	9.32	Howardella ureilytica	Howardella		Clostridiales	Clostridia	Firmicutes	Terrabacteria group
Promega_1A_UTI	UTI	28264	0.05%	9.32	Arcanobacterium haemolyticum	Arcanobacterium	Actinomycetaceae	Actinomycetales	Actinobacteria	Actinobacteria	Terrabacteria group
Promega_1A_UTI	UTI	unmapped	0.00%	4.00							
Promega_1A_UTI	UTI	mapped_unclassified	0.00%	1 388.00							
PC_1A_UTI	UTI	545	96.86%	18 510.55	Citrobacter koseri	Citrobacter	Enterobacteriaceae	Enterobacterales	Gammaproteobacteria	Proteobacteria	
PC_1A_UTI	UTI	2490633	0.70%	133.69	Streptococcus periodonticum	Streptococcus	Streptococcaceae	Lactobacillales	Bacilli	Firmicutes	Terrabacteria group
PC_1A_UTI	UTI	386414	0.53%	101.72	Prevotella timonensis	Prevotella	Prevotellaceae	Bacteroidales	Bacteroidia	Bacteroidetes	FCB group
PC_1A_UTI	UTI	1852374	0.50%	95.91	Ezakiella massiliensis	Ezakiella			Tissierellia	Firmicutes	Terrabacteria group
PC_1A_UTI	UTI	669455	0.34%	64.91	Sediminibacterium magnilacihabitans	Sediminibacterium	Chitinophagaceae	Chitinophagales	Chitinophagia	Bacteroidetes	FCB group
PC_1A_UTI	UTI	1702287	0.25%	47.47	Negativibacillus massiliensis	Negativicoccus	Veillonellaceae	Veillonellales	Negativicutes	Firmicutes	Terrabacteria group
PC_1A_UTI	UTI	1376	0.23%	44.56	Aerococcus urinae	Aerococcus	Aerococcaceae	Lactobacillales	Bacilli	Firmicutes	Terrabacteria group
PC_1A_UTI	UTI	827	0.14%	27.13	Campylobacter ureolyticus	Campylobacter	Campylobacteraceae	Campylobacterales	Epsilonproteobacteria	Proteobacteria	
PC_1A_UTI	UTI	322095	0.11%	21.31	Porphyromonas somerae	Porphyromonas	Porphyromonadaceae	Bacteroidales	Bacteroidia	Bacteroidetes	FCB group
PC_1A_UTI	UTI	33012	0.11%	21.31	Propionimicrobium lymphophilum	Propionimicrobium	Propionibacteriaceae	Propionibacteriales	Actinobacteria	Actinobacteria	Terrabacteria group
PC_1A_UTI	UTI	190146	0.09%	16.47	Actinotignum urinale	Actinotignum	Actinomycetaceae	Actinomycetales	Actinobacteria	Actinobacteria	Terrabacteria group
PC_1A_UTI	UTI	562	0.09%	16.27	Escherichia coli	Escherichia	Enterobacteriaceae	Enterobacterales	Gammaproteobacteria	Proteobacteria	
PC_1A_UTI	UTI	507750	0.05%	9.69	Peptoniphilus duerdenii	Peptoniphilus	Peptoniphilaceae	Tissierellales	Tissierellia	Firmicutes	Terrabacteria group
PC_1A_UTI	UTI	unmapped	0.00%	6.00							
PC_1A_UTI	UTI	mapped_unclassified	0.00%	694.00							
IL-DEx_2A_UTI	UTI	562	74.75%	101.65	Escherichia coli	Escherichia	Enterobacteriaceae	Enterobacterales	Gammaproteobacteria	Proteobacteria	
IL-DEx_2A_UTI	UTI	1351	5.76%	7.83	Enterococcus faecalis	Enterococcus	Enterococcaceae	Lactobacillales	Bacilli	Firmicutes	Terrabacteria group
IL-DEx_2A_UTI	UTI	1261	5.04%	6.85	Peptostreptococcus anaerobius	Peptostreptococcus	Peptostreptococcaceae	Clostridiales	Clostridia	Firmicutes	Terrabacteria group
IL-DEx_2A_UTI	UTI	545	3.66%	4.98	Citrobacter koseri	Citrobacter	Enterobacteriaceae	Enterobacterales	Gammaproteobacteria	Proteobacteria	
IL-DEx_2A_UTI	UTI	573	3.61%	4.91	Klebsiella pneumoniae	Klebsiella	Enterobacteriaceae	Enterobacterales	Gammaproteobacteria	Proteobacteria	
IL-DEx_2A_UTI	UTI	818	2.16%	2.94	Bacteroides thetaiotaomicron	Bacteroides	Bacteroidaceae	Bacteroidales	Bacteroidia	Bacteroidetes	FCB group
IL-DEx_2A_UTI	UTI	37482	1.44%	1.96	Laceyella sacchari	Laceyella	Thermoactinomycetaceae	Bacillales	Bacilli	Firmicutes	Terrabacteria group
IL-DEx_2A_UTI	UTI	287	1.44%	1.96	Pseudomonas aeruginosa	Pseudomonas	Pseudomonadaceae	Pseudomonadales	Gammaproteobacteria	Proteobacteria	
IL-DEx_2A_UTI	UTI	693444	0.72%	0.98	Enterobacteriaceae bacterium strain FGI 57		Enterobacteriaceae	Enterobacterales	Gammaproteobacteria	Proteobacteria	
IL-DEx_2A_UTI	UTI	197614	0.72%	0.98	Streptococcus gallolyticus subsp. pasteurianus	Streptococcus	Streptococcaceae	Lactobacillales	Bacilli	Firmicutes	Terrabacteria group
IL-DEx_2A_UTI	UTI	1871034	0.72%	0.98	Propionimicrobium sp. Marseille-P3275	Propionimicrobium	Propionibacteriaceae	Propionibacteriales	Actinobacteria	Actinobacteria	Terrabacteria group
IL-DEx_2A_UTI	UTI	unmapped	0.00%	1 390.00							
IL-DEx_2A_UTI	UTI	mapped_unclassified	0.00%	3.00							
QIAGEN_2A_UTI	UTI	562	76.28%	14 913.64	Escherichia coli	Escherichia	Enterobacteriaceae	Enterobacterales	Gammaproteobacteria	Proteobacteria	
QIAGEN_2A_UTI	UTI	1261	15.72%	3 074.28	Peptostreptococcus anaerobius	Peptostreptococcus	Peptostreptococcaceae	Clostridiales	Clostridia	Firmicutes	Terrabacteria group
QIAGEN_2A_UTI	UTI	1351	3.79%	740.84	Enterococcus faecalis	Enterococcus	Enterococcaceae	Lactobacillales	Bacilli	Firmicutes	Terrabacteria group
QIAGEN_2A_UTI	UTI	28125	1.78%	347.64	Prevotella bivia	Prevotella	Prevotellaceae	Bacteroidales	Bacteroidia	Bacteroidetes	FCB group
QIAGEN_2A_UTI	UTI	818	1.31%	256.52	Bacteroides thetaiotaomicron	Bacteroides	Bacteroidaceae	Bacteroidales	Bacteroidia	Bacteroidetes	FCB group
QIAGEN_2A_UTI	UTI	584	0.68%	132.72	Proteus mirabilis	Proteus	Morganellaceae	Enterobacterales	Gammaproteobacteria	Proteobacteria	
QIAGEN_2A_UTI	UTI	2490633	0.19%	36.65	Streptococcus periodonticum	Streptococcus	Streptococcaceae	Lactobacillales	Bacilli	Firmicutes	Terrabacteria group
QIAGEN_2A_UTI	UTI	623	0.08%	14.88	Shigella flexneri	Shigella	Enterobacteriaceae	Enterobacterales	Gammaproteobacteria	Proteobacteria	
QIAGEN_2A_UTI	UTI	622	0.07%	13.04	Shigella dysenteriae	Shigella	Enterobacteriaceae	Enterobacterales	Gammaproteobacteria	Proteobacteria	
QIAGEN_2A_UTI	UTI	28116	0.06%	10.90	Bacteroides ovatus	Bacteroides	Bacteroidaceae	Bacteroidales	Bacteroidia	Bacteroidetes	FCB group
QIAGEN_2A_UTI	UTI	1260	0.06%	10.89	Finegoldia magna	Finegoldia	Peptoniphilaceae	Tissierellales	Tissierellia	Firmicutes	Terrabacteria group
QIAGEN_2A_UTI	UTI	unmapped	0.00%	9.00							
QIAGEN_2A_UTI	UTI	mapped_unclassified	0.00%	205.00							
Promega_2A_UTI	UTI	562	81.96%	16 104.16	Escherichia coli	Escherichia	Enterobacteriaceae	Enterobacterales	Gammaproteobacteria	Proteobacteria	
Promega_2A_UTI	UTI	818	6.88%	1 352.59	Bacteroides thetaiotaomicron	Bacteroides	Bacteroidaceae	Bacteroidales	Bacteroidia	Bacteroidetes	FCB group
Promega_2A_UTI	UTI	1261	4.39%	862.38	Peptostreptococcus anaerobius	Peptostreptococcus	Peptostreptococcaceae	Clostridiales	Clostridia	Firmicutes	Terrabacteria group
Promega_2A_UTI	UTI	28125	3.26%	640.26	Prevotella bivia	Prevotella	Prevotellaceae	Bacteroidales	Bacteroidia	Bacteroidetes	FCB group
Promega_2A_UTI	UTI	584	3.03%	594.46	Proteus mirabilis	Proteus	Morganellaceae	Enterobacterales	Gammaproteobacteria	Proteobacteria	
Promega_2A_UTI	UTI	28116	0.23%	45.36	Bacteroides ovatus	Bacteroides	Bacteroidaceae	Bacteroidales	Bacteroidia	Bacteroidetes	FCB group
Promega_2A_UTI	UTI	2490633	0.15%	28.88	Streptococcus periodonticum	Streptococcus	Streptococcaceae	Lactobacillales	Bacilli	Firmicutes	Terrabacteria group
Promega_2A_UTI	UTI	1260	0.06%	11.95	Finegoldia magna	Finegoldia	Peptoniphilaceae	Tissierellales	Tissierellia	Firmicutes	Terrabacteria group
Promega_2A_UTI	UTI	1351	0.05%	9.96	Enterococcus faecalis	Enterococcus	Enterococcaceae	Lactobacillales	Bacilli	Firmicutes	Terrabacteria group
Promega_2A_UTI	UTI	unmapped	0.00%	14.00							
Promega_2A_UTI	UTI	mapped_unclassified	0.00%	99.00							
PC_2A_UTI	UTI	562	73.20%	14 264.19	Escherichia coli	Escherichia	Enterobacteriaceae	Enterobacterales	Gammaproteobacteria	Proteobacteria	
PC_2A_UTI	UTI	1261	16.91%	3 294.97	Peptostreptococcus anaerobius	Peptostreptococcus	Peptostreptococcaceae	Clostridiales	Clostridia	Firmicutes	Terrabacteria group
PC_2A_UTI	UTI	1351	3.82%	744.91	Enterococcus faecalis	Enterococcus	Enterococcaceae	Lactobacillales	Bacilli	Firmicutes	Terrabacteria group
PC_2A_UTI	UTI	818	2.54%	495.79	Bacteroides thetaiotaomicron	Bacteroides	Bacteroidaceae	Bacteroidales	Bacteroidia	Bacteroidetes	FCB group
PC_2A_UTI	UTI	28125	1.85%	360.63	Prevotella bivia	Prevotella	Prevotellaceae	Bacteroidales	Bacteroidia	Bacteroidetes	FCB group
PC_2A_UTI	UTI	584	0.96%	187.21	Proteus mirabilis	Proteus	Morganellaceae	Enterobacterales	Gammaproteobacteria	Proteobacteria	
PC_2A_UTI	UTI	2490633	0.29%	56.16	Streptococcus periodonticum	Streptococcus	Streptococcaceae	Lactobacillales	Bacilli	Firmicutes	Terrabacteria group
PC_2A_UTI	UTI	1260	0.26%	50.25	Finegoldia magna	Finegoldia	Peptoniphilaceae	Tissierellales	Tissierellia	Firmicutes	Terrabacteria group
PC_2A_UTI	UTI	623	0.11%	20.63	Shigella flexneri	Shigella	Enterobacteriaceae	Enterobacterales	Gammaproteobacteria	Proteobacteria	
PC_2A_UTI	UTI	622	0.06%	11.24	Shigella dysenteriae	Shigella	Enterobacteriaceae	Enterobacterales	Gammaproteobacteria	Proteobacteria	
PC_2A_UTI	UTI	unmapped	0.00%	2.00							
PC_2A_UTI	UTI	mapped_unclassified	0.00%	305.00							
IL-DEx_3A_UTI	UTI	562	99.75%	19 578.12	Escherichia coli	Escherichia	Enterobacteriaceae	Enterobacterales	Gammaproteobacteria	Proteobacteria	
IL-DEx_3A_UTI	UTI	669455	0.16%	31.92	Sediminibacterium magnilacihabitans	Sediminibacterium	Chitinophagaceae	Chitinophagales	Chitinophagia	Bacteroidetes	FCB group
IL-DEx_3A_UTI	UTI	34062	0.09%	16.96	Moraxella osloensis	Moraxella	Moraxellaceae	Pseudomonadales	Gammaproteobacteria	Proteobacteria	
IL-DEx_3A_UTI	UTI	unmapped	0.00%	55.00							
IL-DEx_3A_UTI	UTI	mapped_unclassified	0.00%	58.00							
QIAGEN_3A_UTI	UTI	562	100.00%	19 637.00	Escherichia coli	Escherichia	Enterobacteriaceae	Enterobacterales	Gammaproteobacteria	Proteobacteria	
QIAGEN_3A_UTI	UTI	unmapped	0.00%	107.00							
QIAGEN_3A_UTI	UTI	mapped_unclassified	0.00%	2.00							
Promega_3A_UTI	UTI	562	99.92%	19 567.77	Escherichia coli	Escherichia	Enterobacteriaceae	Enterobacterales	Gammaproteobacteria	Proteobacteria	
Promega_3A_UTI	UTI	1261	0.08%	15.23	Peptostreptococcus anaerobius	Peptostreptococcus	Peptostreptococcaceae	Clostridiales	Clostridia	Firmicutes	Terrabacteria group
Promega_3A_UTI	UTI	unmapped	0.00%	45.00							
Promega_3A_UTI	UTI	mapped_unclassified	0.00%	38.00							
12_PC_3A_UTI	UTI	562	100.00%	18 946.00	Escherichia coli	Escherichia	Enterobacteriaceae	Enterobacterales	Gammaproteobacteria	Proteobacteria	
12_PC_3A_UTI	UTI	unmapped	0.00%	345.00							
12_PC_3A_UTI	UTI	mapped_unclassified	0.00%	14.00							
IL-DEx_4A_UTI	UTI	562	93.29%	17 854.33	Escherichia coli	Escherichia	Enterobacteriaceae	Enterobacterales	Gammaproteobacteria	Proteobacteria	
IL-DEx_4A_UTI	UTI	1311	2.51%	480.76	Streptococcus agalactiae	Streptococcus	Streptococcaceae	Lactobacillales	Bacilli	Firmicutes	Terrabacteria group
IL-DEx_4A_UTI	UTI	197614	1.22%	234.14	Streptococcus gallolyticus subsp. pasteurianus	Streptococcus	Streptococcaceae	Lactobacillales	Bacilli	Firmicutes	Terrabacteria group
IL-DEx_4A_UTI	UTI	47770	0.89%	169.94	Lactobacillus crispatus	Lactobacillus	Lactobacillaceae	Lactobacillales	Bacilli	Firmicutes	Terrabacteria group
IL-DEx_4A_UTI	UTI	1351	0.71%	135.01	Enterococcus faecalis	Enterococcus	Enterococcaceae	Lactobacillales	Bacilli	Firmicutes	Terrabacteria group
IL-DEx_4A_UTI	UTI	187328	0.70%	133.07	Veillonella montpellierensis	Veillonella	Veillonellaceae	Veillonellales	Negativicutes	Firmicutes	Terrabacteria group
IL-DEx_4A_UTI	UTI	134821	0.21%	40.80	Ureaplasma parvum	Ureaplasma	Mycoplasmataceae	Mycoplasmatales	Mollicutes	Tenericutes	Terrabacteria group

IL-DEx_4A_UTI	UTI	109790	0.17%	32.09	Lactobacillus jensenii	Lactobacillus	Lactobacillaceae	Lactobacillales	Bacilli	Firmicutes	Terrabacteria group
IL-DEx_4A_UTI	UTI	147802	0.14%	27.20	Lactobacillus iners	Lactobacillus	Lactobacillaceae	Lactobacillales	Bacilli	Firmicutes	Terrabacteria group
IL-DEx_4A_UTI	UTI	2702	0.08%	15.54	Gardnerella vaginalis	Gardnerella	Bifidobacteriaceae	Bifidobacteriales	Actinobacteria	Actinobacteria	Terrabacteria group
IL-DEx_4A_UTI	UTI	623	0.08%	15.12	Shigella flexneri	Shigella	Enterobacteriaceae	Enterobacterales	Gammaproteobacteria	Proteobacteria	
IL-DEx_4A_UTI	UTI	unmapped	0.00%	31.00							
IL-DEx_4A_UTI	UTI	mapped_unclassified	0.00%	598.00							
QIAGEN_4A_UTI	UTI	562	95.09%	18 362.89	Escherichia coli	Escherichia	Enterobacteriaceae	Enterobacterales	Gammaproteobacteria	Proteobacteria	
QIAGEN_4A_UTI	UTI	187328	1.49%	287.70	Veillonella montpellierensis	Veillonella	Veillonellaceae	Veillonellales	Negativicutes	Firmicutes	Terrabacteria group
QIAGEN_4A_UTI	UTI	197614	1.27%	244.52	Streptococcus gallolyticus subsp. pasteurianus	Streptococcus	Streptococcaceae	Lactobacillales	Bacilli	Firmicutes	Terrabacteria group
QIAGEN_4A_UTI	UTI	47770	0.95%	182.99	Lactobacillus crispatus	Lactobacillus	Lactobacillaceae	Lactobacillales	Bacilli	Firmicutes	Terrabacteria group
QIAGEN_4A_UTI	UTI	134821	0.40%	76.33	Ureaplasma parvum	Ureaplasma	Mycoplasmataceae	Mycoplasmatales	Mollicutes	Tenericutes	Terrabacteria group
QIAGEN_4A_UTI	UTI	109790	0.29%	56.76	Lactobacillus jensenii	Lactobacillus	Lactobacillaceae	Lactobacillales	Bacilli	Firmicutes	Terrabacteria group
QIAGEN_4A_UTI	UTI	147802	0.21%	40.12	Lactobacillus iners	Lactobacillus	Lactobacillaceae	Lactobacillales	Bacilli	Firmicutes	Terrabacteria group
QIAGEN_4A_UTI	UTI	2702	0.20%	38.16	Gardnerella vaginalis	Gardnerella	Bifidobacteriaceae	Bifidobacteriales	Actinobacteria	Actinobacteria	Terrabacteria group
QIAGEN_4A_UTI	UTI	1351	0.06%	11.74	Enterococcus faecalis	Enterococcus	Enterococcaceae	Lactobacillales	Bacilli	Firmicutes	Terrabacteria group
QIAGEN_4A_UTI	UTI	502393	0.05%	9.79	Gemella asaccharolytica	Gemella		Bacillales	Bacilli	Firmicutes	Terrabacteria group
QIAGEN_4A_UTI	UTI	unmapped	0.00%	20.00							
QIAGEN_4A_UTI	UTI	mapped_unclassified	0.00%	454.00							
Promega_4A_UTI	UTI	562	89.26%	17 179.22	Escherichia coli	Escherichia	Enterobacteriaceae	Enterobacterales	Gammaproteobacteria	Proteobacteria	
Promega_4A_UTI	UTI	1311	6.83%	1 314.89	Streptococcus agalactiae	Streptococcus	Streptococcaceae	Lactobacillales	Bacilli	Firmicutes	Terrabacteria group
Promega_4A_UTI	UTI	197614	1.30%	249.34	Streptococcus gallolyticus subsp. pasteurianus	Streptococcus	Streptococcaceae	Lactobacillales	Bacilli	Firmicutes	Terrabacteria group
Promega_4A_UTI	UTI	47770	0.85%	162.66	Lactobacillus crispatus	Lactobacillus	Lactobacillaceae	Lactobacillales	Bacilli	Firmicutes	Terrabacteria group
Promega_4A_UTI	UTI	1351	0.59%	112.98	Enterococcus faecalis	Enterococcus	Enterococcaceae	Lactobacillales	Bacilli	Firmicutes	Terrabacteria group
Promega_4A_UTI	UTI	187328	0.55%	105.19	Veillonella montpellierensis	Veillonella	Veillonellaceae	Veillonellales	Negativicutes	Firmicutes	Terrabacteria group
Promega_4A_UTI	UTI	109790	0.17%	33.12	Lactobacillus jensenii	Lactobacillus	Lactobacillaceae	Lactobacillales	Bacilli	Firmicutes	Terrabacteria group
Promega_4A_UTI	UTI	2490633	0.13%	24.35	Streptococcus periodonticum	Streptococcus	Streptococcaceae	Lactobacillales	Bacilli	Firmicutes	Terrabacteria group
Promega_4A_UTI	UTI	134821	0.10%	18.51	Ureaplasma parvum	Ureaplasma	Mycoplasmataceae	Mycoplasmatales	Mollicutes	Tenericutes	Terrabacteria group
Promega_4A_UTI	UTI	502393	0.09%	16.56	Gemella asaccharolytica	Gemella		Bacillales	Bacilli	Firmicutes	Terrabacteria group
Promega_4A_UTI	UTI	2702	0.08%	15.58	Gardnerella vaginalis	Gardnerella	Bifidobacteriaceae	Bifidobacteriales	Actinobacteria	Actinobacteria	Terrabacteria group
Promega_4A_UTI	UTI	147802	0.08%	14.61	Lactobacillus iners	Lactobacillus	Lactobacillaceae	Lactobacillales	Bacilli	Firmicutes	Terrabacteria group
Promega_4A_UTI	UTI	unmapped	0.00%	3.00							
Promega_4A_UTI	UTI	mapped_unclassified	0.00%	529.00							
PC_4A_UTI	UTI	562	87.38%	16 676.45	Escherichia coli	Escherichia	Enterobacteriaceae	Enterobacterales	Gammaproteobacteria	Proteobacteria	
PC_4A_UTI	UTI	187328	6.30%	1 202.81	Veillonella montpellierensis	Veillonella	Veillonellaceae	Veillonellales	Negativicutes	Firmicutes	Terrabacteria group
PC_4A_UTI	UTI	47770	1.83%	349.00	Lactobacillus crispatus	Lactobacillus	Lactobacillaceae	Lactobacillales	Bacilli	Firmicutes	Terrabacteria group
PC_4A_UTI	UTI	197614	1.38%	263.63	Streptococcus gallolyticus subsp. pasteurianus	Streptococcus	Streptococcaceae	Lactobacillales	Bacilli	Firmicutes	Terrabacteria group
PC_4A_UTI	UTI	1311	1.00%	189.97	Streptococcus agalactiae	Streptococcus	Streptococcaceae	Lactobacillales	Bacilli	Firmicutes	Terrabacteria group
PC_4A_UTI	UTI	134821	0.66%	125.03	Ureaplasma parvum	Ureaplasma	Mycoplasmataceae	Mycoplasmatales	Mollicutes	Tenericutes	Terrabacteria group
PC_4A_UTI	UTI	147802	0.51%	97.89	Lactobacillus iners	Lactobacillus	Lactobacillaceae	Lactobacillales	Bacilli	Firmicutes	Terrabacteria group
PC_4A_UTI	UTI	109790	0.45%	85.21	Lactobacillus jensenii	Lactobacillus	Lactobacillaceae	Lactobacillales	Bacilli	Firmicutes	Terrabacteria group
PC_4A_UTI	UTI	1351	0.15%	28.11	Enterococcus faecalis	Enterococcus	Enterococcaceae	Lactobacillales	Bacilli	Firmicutes	Terrabacteria group
PC_4A_UTI	UTI	54005	0.13%	25.20	Peptoniphilus harei	Peptoniphilus	Peptoniphilaceae	Tissierellales	Tissierellia	Firmicutes	Terrabacteria group
PC_4A_UTI	UTI	2702	0.10%	18.42	Gardnerella vaginalis	Gardnerella	Bifidobacteriaceae	Bifidobacteriales	Actinobacteria	Actinobacteria	Terrabacteria group
PC_4A_UTI	UTI	1260	0.06%	11.63	Finegoldia magna	Finegoldia	Peptoniphilaceae	Tissierellales	Tissierellia	Firmicutes	Terrabacteria group
PC_4A_UTI	UTI	502393	0.06%	10.66	Gemella asaccharolytica	Gemella		Bacillales	Bacilli	Firmicutes	Terrabacteria group
PC_4A_UTI	UTI	unmapped	0.00%	6.00							
PC_4A_UTI	UTI	mapped_unclassified	0.00%	639.00							
IL-DEx_5A_UTI	UTI	562	97.06%	19 057.15	Escherichia coli	Escherichia	Enterobacteriaceae	Enterobacterales	Gammaproteobacteria	Proteobacteria	
IL-DEx_5A_UTI	UTI	147802	2.39%	468.45	Lactobacillus iners	Lactobacillus	Lactobacillaceae	Lactobacillales	Bacilli	Firmicutes	Terrabacteria group
IL-DEx_5A_UTI	UTI	109790	0.38%	74.59	Lactobacillus jensenii	Lactobacillus	Lactobacillaceae	Lactobacillales	Bacilli	Firmicutes	Terrabacteria group
IL-DEx_5A_UTI	UTI	2702	0.18%	34.81	Gardnerella vaginalis	Gardnerella	Bifidobacteriaceae	Bifidobacteriales	Actinobacteria	Actinobacteria	Terrabacteria group
IL-DEx_5A_UTI	UTI	unmapped	0.00%	2.00							
IL-DEx_5A_UTI	UTI	mapped_unclassified	0.00%	109.00							
QIAGEN_5A_UTI	UTI	562	98.54%	19 146.18	Escherichia coli	Escherichia	Enterobacteriaceae	Enterobacterales	Gammaproteobacteria	Proteobacteria	
QIAGEN_5A_UTI	UTI	147802	0.95%	184.29	Lactobacillus iners	Lactobacillus	Lactobacillaceae	Lactobacillales	Bacilli	Firmicutes	Terrabacteria group
QIAGEN_5A_UTI	UTI	109790	0.20%	39.42	Lactobacillus jensenii	Lactobacillus	Lactobacillaceae	Lactobacillales	Bacilli	Firmicutes	Terrabacteria group
QIAGEN_5A_UTI	UTI	1351	0.20%	38.43	Enterococcus faecalis	Enterococcus	Enterococcaceae	Lactobacillales	Bacilli	Firmicutes	Terrabacteria group
QIAGEN_5A_UTI	UTI	2702	0.11%	21.68	Gardnerella vaginalis	Gardnerella	Bifidobacteriaceae	Bifidobacteriales	Actinobacteria	Actinobacteria	Terrabacteria group
QIAGEN_5A_UTI	UTI	unmapped	0.00%	3.00							
QIAGEN_5A_UTI	UTI	mapped_unclassified	0.00%	320.00							
Promega_5A_UTI	UTI	562	98.88%	19 338.15	Escherichia coli	Escherichia	Enterobacteriaceae	Enterobacterales	Gammaproteobacteria	Proteobacteria	
Promega_5A_UTI	UTI	147802	0.76%	148.50	Lactobacillus iners	Lactobacillus	Lactobacillaceae	Lactobacillales	Bacilli	Firmicutes	Terrabacteria group
Promega_5A_UTI	UTI	109790	0.15%	28.71	Lactobacillus jensenii	Lactobacillus	Lactobacillaceae	Lactobacillales	Bacilli	Firmicutes	Terrabacteria group
Promega_5A_UTI	UTI	2702	0.11%	21.78	Gardnerella vaginalis	Gardnerella	Bifidobacteriaceae	Bifidobacteriales	Actinobacteria	Actinobacteria	Terrabacteria group
Promega_5A_UTI	UTI	623	0.11%	20.86	Shigella flexneri	Shigella	Enterobacteriaceae	Enterobacterales	Gammaproteobacteria	Proteobacteria	
Promega_5A_UTI	UTI	unmapped	0.00%	12.00							
Promega_5A_UTI	UTI	mapped_unclassified	0.00%	198.00							
PC_5A_UTI	UTI	562	99.50%	19 572.71	Escherichia coli	Escherichia	Enterobacteriaceae	Enterobacterales	Gammaproteobacteria	Proteobacteria	
PC_5A_UTI	UTI	1351	0.15%	28.87	Enterococcus faecalis	Enterococcus	Enterococcaceae	Lactobacillales	Bacilli	Firmicutes	Terrabacteria group
PC_5A_UTI	UTI	147802	0.14%	26.88	Lactobacillus iners	Lactobacillus	Lactobacillaceae	Lactobacillales	Bacilli	Firmicutes	Terrabacteria group
PC_5A_UTI	UTI	669455	0.09%	16.93	Sediminibacterium magnilacihabitans	Sediminibacterium	Chitinophagaceae	Chitinophagales	Chitinophagia	Bacteroidetes	FCB group
PC_5A_UTI	UTI	1596	0.08%	14.93	Lactobacillus gasseri	Lactobacillus	Lactobacillaceae	Lactobacillales	Bacilli	Firmicutes	Terrabacteria group
PC_5A_UTI	UTI	623	0.06%	11.68	Shigella flexneri	Shigella	Enterobacteriaceae	Enterobacterales	Gammaproteobacteria	Proteobacteria	
PC_5A_UTI	UTI	unmapped	0.00%	1.00							
PC_5A_UTI	UTI	mapped_unclassified	0.00%	94.00							
IL-DEx_6A_UTI	UTI	562	99.75%	19 548.06	Escherichia coli	Escherichia	Enterobacteriaceae	Enterobacterales	Gammaproteobacteria	Proteobacteria	
IL-DEx_6A_UTI	UTI	404403	0.11%	20.96	Howardella ureilytica	Howardella		Clostridiales	Clostridia	Firmicutes	Terrabacteria group
IL-DEx_6A_UTI	UTI	1338	0.08%	15.97	Streptococcus intermedius	Streptococcus	Streptococcaceae	Lactobacillales	Bacilli	Firmicutes	Terrabacteria group
IL-DEx_6A_UTI	UTI	28901	0.06%	12.01	Salmonella enterica	Salmonella	Enterobacteriaceae	Enterobacterales	Gammaproteobacteria	Proteobacteria	
IL-DEx_6A_UTI	UTI	unmapped	0.00%	12.00							
IL-DEx_6A_UTI	UTI	mapped_unclassified	0.00%	61.00							
QIAGEN_6A_UTI	UTI	562	99.60%	19 558.31	Escherichia coli	Escherichia	Enterobacteriaceae	Enterobacterales	Gammaproteobacteria	Proteobacteria	
QIAGEN_6A_UTI	UTI	1338	0.40%	77.69	Streptococcus intermedius	Streptococcus	Streptococcaceae	Lactobacillales	Bacilli	Firmicutes	Terrabacteria group
QIAGEN_6A_UTI	UTI	unmapped	0.00%	25.00							
QIAGEN_6A_UTI	UTI	mapped_unclassified	0.00%	93.00							
Promega_6A_UTI	UTI	562	99.56%	19 581.25	Escherichia coli	Escherichia	Enterobacteriaceae	Enterobacterales	Gammaproteobacteria	Proteobacteria	
Promega_6A_UTI	UTI	1338	0.28%	55.23	Streptococcus intermedius	Streptococcus	Streptococcaceae	Lactobacillales	Bacilli	Firmicutes	Terrabacteria group
Promega_6A_UTI	UTI	28901	0.10%	18.98	Salmonella enterica	Salmonella	Enterobacteriaceae	Enterobacterales	Gammaproteobacteria	Proteobacteria	
Promega_6A_UTI	UTI	33040	0.06%	11.55	Streptococcus anginosus	Streptococcus	Streptococcaceae	Lactobacillales	Bacilli	Firmicutes	Terrabacteria group
Promega_6A_UTI	UTI	unmapped	0.00%	6.00							
Promega_6A_UTI	UTI	mapped_unclassified	0.00%	81.00							
PC_6A_UTI	UTI	562	98.34%	19 387.58	Escherichia coli	Escherichia	Enterobacteriaceae	Enterobacterales	Gammaproteobacteria	Proteobacteria	
PC_6A_UTI	UTI	1338	1.29%	254.30	Streptococcus intermedius	Streptococcus	Streptococcaceae	Lactobacillales	Bacilli	Firmicutes	Terrabacteria group
PC_6A_UTI	UTI	33040	0.19%	37.19	Streptococcus anginosus	Streptococcus	Streptococcaceae	Lactobacillales	Bacilli	Firmicutes	Terrabacteria group
PC_6A_UTI	UTI	404403	0.18%	34.94	Howardella ureilytica	Howardella		Clostridiales	Clostridia	Firmicutes	Terrabacteria group
PC_6A_UTI	UTI	unmapped	0.00%	3.00							
PC_6A_UTI	UTI	mapped_unclassified	0.00%	51.00							
IL-DEx_8A_UTI	UTI	562	100.00%	19 775.00	Escherichia coli	Escherichia	Enterobacteriaceae	Enterobacterales	Gammaproteobacteria	Proteobacteria	
IL-DEx_8A_UTI	UTI	unmapped	0.00%	5.00							
IL-DEx_8A_UTI	UTI	mapped_unclassified	0.00%	13.00							
QIAGEN_8A_UTI	UTI	562	100.00%	19 694.00	Escherichia coli	Escherichia	Enterobacteriaceae	Enterobacterales	Gammaproteobacteria	Proteobacteria	
QIAGEN_8A_UTI	UTI	unmapped	0.00%	36.00							
QIAGEN_8A_UTI	UTI	mapped_unclassified	0.00%	12.00							
Promega_8A_UTI	UTI	562	100.00%	19 737.00	Escherichia coli	Escherichia	Enterobacteriaceae	Enterobacterales	Gammaproteobacteria	Proteobacteria	
Promega_8A_UTI	UTI	unmapped	0.00%	11.00							
Promega_8A_UTI	UTI	mapped_unclassified	0.00%	7.00							
28_PC_8A_UTI	UTI	562	100.00%	19 679.00	Escherichia coli	Escherichia	Enterobacteriaceae	Enterobacterales	Gammaproteobacteria	Proteobacteria	
28_PC_8A_UTI	UTI	unmapped	0.00%	41.00							
28_PC_8A_UTI	UTI	mapped_unclassified	0.00%	12.00							
IL-DEx_7A_UTI	UTI	573	100.00%	19 782.00	Klebsiella pneumoniae	Klebsiella	Enterobacteriaceae	Enterobacterales	Gammaproteobacteria	Proteobacteria	
IL-DEx_7A_UTI	UTI	unmapped	0.00%	14.00							
IL-DEx_7A_UTI	UTI	mapped_unclassified	0.00%	10.00							
QIAGEN_7A_UTI	UTI	573	99.94%	19 737.24	Klebsiella pneumoniae	Klebsiella	Enterobacteriaceae	Enterobacterales	Gammaproteobacteria	Proteobacteria	
QIAGEN_7A_UTI	UTI	562	0.06%	12.76	Escherichia coli	Escherichia	Enterobacteriaceae	Enterobacterales	Gammaproteobacteria	Proteobacteria	
QIAGEN_7A_UTI	UTI	unmapped	0.00%	16.00							
QIAGEN_7A_UTI	UTI	mapped_unclassified	0.00%	14.00							
Promega_7A_UTI	UTI	573	99.89%	19 737.82	Klebsiella pneumoniae	Klebsiella	Enterobacteriaceae	Enterobacterales	Gammaproteobacteria	Proteobacteria	
Promega_7A_UTI	UTI	562	0.11%	21.18	Escherichia coli	Escherichia	Enterobacteriaceae	Enterobacterales	Gammaproteobacteria	Proteobacteria	
Promega_7A_UTI	UTI	unmapped	0.00%	2.00							
Promega_7A_UTI	UTI	mapped_unclassified	0.00%	18.00							
PC_7A_UTI	UTI	573	99.91%	19 748.89	Klebsiella pneumoniae	Klebsiella	Enterobacteriaceae	Enterobacterales	Gammaproteobacteria	Proteobacteria	
PC_7A_UTI	UTI	562	0.09%	17.11	Escherichia coli	Escherichia	Enterobacteriaceae	Enterobacterales	Gammaproteobacteria	Proteobacteria	
PC_7A_UTI	UTI	unmapped	0.00%	26.00							
PC_7A_UTI	UTI	mapped_unclassified	0.00%	17.00							
IL-DEx_9A_UTI	UTI	562	100.00%	19 460.00	Escherichia coli	Escherichia	Enterobacteriaceae	Enterobacterales	Gammaproteobacteria	Proteobacteria	
IL-DEx_9A_UTI	UTI	unmapped	0.00%	54.00							
IL-DEx_9A_UTI	UTI	mapped_unclassified	0.00%	16.00							
QIAGEN_9A_UTI	UTI	562	100.00%	19 598.00	Escherichia coli	Escherichia	Enterobacteriaceae	Enterobacterales	Gammaproteobacteria	Proteobacteria	
QIAGEN_9A_UTI	UTI	unmapped	0.00%	40.00							
QIAGEN_9A_UTI	UTI	mapped_unclassified	0.00%	7.00							
Promega_9A_UTI	UTI	562	100.00%	19 492.00	Escherichia coli	Escherichia	Enterobacteriaceae	Enterobacterales	Gammaproteobacteria	Proteobacteria	
Promega_9A_UTI	UTI	unmapped	0.00%	138.00							
Promega_9A_UTI	UTI	mapped_unclassified	0.00%	1.00							
PC_9A_UTI	UTI	562	100.00%	19 556.00	Escherichia coli	Escherichia	Enterobacteriaceae	Enterobacterales	Gammaproteobacteria	Proteobacteria	
PC_9A_UTI	UTI	unmapped	0.00%	116.00							
PC_9A_UTI	UTI	mapped_unclassified	0.00%	7.00							
IL-DEx_10A_UTI	UTI	562	100.00%	19 615.00	Escherichia coli	Escherichia	Enterobacteriaceae	Enterobacterales	Gammaproteobacteria	Proteobacteria	
IL-DEx_10A_UTI	UTI	unmapped	0.00%	56.00							
IL-DEx_10A_UTI	UTI	mapped_unclassified	0.00%	8.00							
QIAGEN_10A_UTI	UTI	287	68.16%	8 043.76	Pseudomonas aeruginosa	Pseudomonas	Pseudomonadaceae	Pseudomonadales	Gammaproteobacteria	Proteobacteria	
QIAGEN_10A_UTI	UTI	562	27.94%	3 297.01	Escherichia coli	Escherichia	Enterobacteriaceae	Enterobacterales	Gammaproteobacteria	Proteobacteria	
QIAGEN_10A_UTI	UTI	364410	1.17%	138.54	Granulibacter bethesdensis	Granulibacter	Acetobacteraceae	Rhodospirillales	Alphaproteobacteria	Proteobacteria	
QIAGEN_10A_UTI	UTI	573	0.98%	115.58	Klebsiella pneumoniae	Klebsiella	Enterobacteriaceae	Enterobacterales	Gammaproteobacteria	Proteobacteria	
QIAGEN_10A_UTI	UTI	29466	0.87%	102.52	Veillonella parvula	Veillonella	Veillonellaceae	Veillonellales	Negativicutes	Firmicutes	Terrabacteria group
QIAGEN_10A_UTI	UTI	545	0.35%	41.01	Citrobacter koseri	Citrobacter	Enterobacteriaceae	Enterobacterales	Gammaproteobacteria	Proteobacteria	
QIAGEN_10A_UTI	UTI	1351	0.22%	26.36	Enterococcus faecalis	Enterococcus	Enterococcaceae	Lactobacillales	Bacilli	Firmicutes	Terrabacteria group
QIAGEN_10A_UTI	UTI	33996	0.12%	13.78	Gluconacetobacter diazotrophicus	Gluconacetobacter	Acetobacteraceae	Rhodospirillales	Alphaproteobacteria	Proteobacteria	
QIAGEN_10A_UTI	UTI	29378	0.12%	13.67	Staphylococcus arlettae	Staphylococcus	Staphylococcaceae	Bacillales	Bacilli	Firmicutes	Terrabacteria group
QIAGEN_10A_UTI	UTI	1747	0.08%	9.76	Cutibacterium acnes	Cutibacterium	Propionibacteriaceae	Propionibacteriales	Actinobacteria	Actinobacteria	Terrabacteria group
QIAGEN_10A_UTI	UTI	unmapped	0.00%	1 644.00							
QIAGEN_10A_UTI	UTI	mapped_unclassified	0.00%	301.00							
Promega_10A_UTI	UTI	287	97.73%	17 449.52	Pseudomonas aeruginosa	Pseudomonas	Pseudomonadaceae	Pseudomonadales	Gammaproteobacteria	Proteobacteria	
Promega_10A_UTI	UTI	29466	1.17%	208.67	Veillonella parvula	Veillonella	Veillonellaceae	Veillonellales	Negativicutes	Firmicutes	Terrabacteria group
Promega_10A_UTI	UTI	562	0.51%	91.25	Escherichia coli	Escherichia	Enterobacteriaceae	Enterobacterales	Gammaproteobacteria	Proteobacteria	
Promega_10A_UTI	UTI	573	0.34%	61.05	Klebsiella pneumoniae	Klebsiella	Enterobacteriaceae	Enterobacterales	Gammaproteobacteria	Proteobacteria	
Promega_10A_UTI	UTI	1351	0.12%	21.76	Enterococcus faecalis	Enterococcus	Enterococcaceae	Lactobacillales	Bacilli	Firmicutes	Terrabacteria group
Promega_10A_UTI	UTI	158836	0.06%	11.37	Enterobacter hormaechei	Enterobacter	Enterobacteriaceae	Enterobacterales	Gammaproteobacteria	Proteobacteria	
Promega_10A_UTI	UTI	550	0.06%	11.37	Enterobacter cloacae	Enterobacter	Enterobacteriaceae	Enterobacterales	Gammaproteobacteria	Proteobacteria	
Promega_10A_UTI	UTI	unmapped	0.00%	1 421.00							
Promega_10A_UTI	UTI	mapped_unclassified	0.00%	209.00							
PC_10A_UTI	UTI	287	91.01%	13 944.28	Pseudomonas aeruginosa	Pseudomonas	Pseudomonadaceae	Pseudomonadales	Gammaproteobacteria	Proteobacteria	
PC_10A_UTI	UTI	669455	4.63%	709.20	Sediminibacterium magnilacihabitans	Sediminibacterium	Chitinophagaceae	Chitinophagales	Chitinophagia	Bacteroidetes	FCB group
PC_10A_UTI	UTI	29466	2.72%	416.82	Veillonella parvula	Veillonella	Veillonellaceae	Veillonellales	Negativicutes	Firmicutes	Terrabacteria group
PC_10A_UTI	UTI	158836	0.77%	117.93	Enterobacter hormaechei	Enterobacter	Enterobacteriaceae	Enterobacterales	Gammaproteobacteria	Proteobacteria	
PC_10A_UTI	UTI	1351	0.49%	75.74	Enterococcus faecalis	Enterococcus	Enterococcaceae	Lactobacillales	Bacilli	Firmicutes	Terrabacteria group
PC_10A_UTI	UTI	562	0.19%	28.53	Escherichia coli	Escherichia	Enterobacteriaceae	Enterobacterales	Gammaproteobacteria	Proteobacteria	
PC_10A_UTI	UTI	488447	0.12%	18.69	Burkholderia contaminans	Burkholderia	Burkholderiaceae	Burkholderiales	Betaproteobacteria	Proteobacteria	
PC_10A_UTI	UTI	202789	0.07%	10.82	Actinobaculum massiliense	Actinobaculum	Actinomycetaceae	Actinomycetales	Actinobacteria	Actinobacteria	Terrabacteria group

PC_10A_UTI	UTI	unmapped	0.00%	3 806.00							
PC_10A_UTI	UTI	mapped_unclassified	0.00%	303.00							
IL-DEx_11A_non-UTI	non-UTI	669455	21.00%	3 877.01	Sediminibacterium magnilacihabitans	Sediminibacterium	Chitinophagaceae	Chitinophagales	Chitinophagia	Bacteroidetes	FCB group
IL-DEx_11A_non-UTI	non-UTI	46507	11.52%	2 125.99	Ezakiella coagulans	Ezakiella			Tissierellia	Firmicutes	Terrabacteria group
IL-DEx_11A_non-UTI	non-UTI	33031	7.55%	1 393.92	Peptoniphilus lacrimalis	Peptoniphilus	Peptoniphilaceae	Tissierellales	Tissierellia	Firmicutes	Terrabacteria group
IL-DEx_11A_non-UTI	non-UTI	2081703	6.54%	1 207.50	Peptostreptococcaceae bacterium oral taxon 929		Peptostreptococcaceae	Clostridiales	Clostridia	Firmicutes	Terrabacteria group
IL-DEx_11A_non-UTI	non-UTI	33012	6.40%	1 181.27	Propionimicrobium lymphophilum	Propionimicrobium	Propionibacteriaceae	Propionibacteriales	Actinobacteria	Actinobacteria	Terrabacteria group
IL-DEx_11A_non-UTI	non-UTI	501496	6.28%	1 159.73	Porphyromonas bennonis	Porphyromonas	Porphyromonadaceae	Bacteroidales	Bacteroidia	Bacteroidetes	FCB group
IL-DEx_11A_non-UTI	non-UTI	54005	6.16%	1 137.73	Peptoniphilus harei	Peptoniphilus	Peptoniphilaceae	Tissierellales	Tissierellia	Firmicutes	Terrabacteria group
IL-DEx_11A_non-UTI	non-UTI	33032	5.66%	1 045.09	Anaerococcus lactolyticus	Anaerococcus	Peptoniphilaceae	Tissierellales	Tissierellia	Firmicutes	Terrabacteria group
IL-DEx_11A_non-UTI	non-UTI	2051	3.42%	631.39	Mobiluncus curtisii	Mobiluncus	Actinomycetaceae	Actinomycetales	Actinobacteria	Actinobacteria	Terrabacteria group
IL-DEx_11A_non-UTI	non-UTI	1465756	2.70%	498.36	Peptoniphilus grossensis	Peptoniphilus	Peptoniphilaceae	Tissierellales	Tissierellia	Firmicutes	Terrabacteria group
IL-DEx_11A_non-UTI	non-UTI	755172	2.18%	402.71	Peptoniphilus coxii	Peptoniphilus	Peptoniphilaceae	Tissierellales	Tissierellia	Firmicutes	Terrabacteria group
IL-DEx_11A_non-UTI	non-UTI	411577	2.15%	397.12	Anaerococcus murdochii	Anaerococcus	Peptoniphilaceae	Tissierellales	Tissierellia	Firmicutes	Terrabacteria group
IL-DEx_11A_non-UTI	non-UTI	308994	1.78%	327.87	Dialister propionicifaciens	Dialister	Veillonellaceae	Veillonellales	Negativicutes	Firmicutes	Terrabacteria group
IL-DEx_11A_non-UTI	non-UTI	1260	1.73%	319.44	Finegoldia magna	Finegoldia	Peptoniphilaceae	Tissierellales	Tissierellia	Firmicutes	Terrabacteria group
IL-DEx_11A_non-UTI	non-UTI	827	1.53%	282.91	Campylobacter ureolyticus	Campylobacter	Campylobacteraceae	Campylobacterales	Epsilonproteobacteria	Proteobacteria	
IL-DEx_11A_non-UTI	non-UTI	1376	1.43%	264.17	Aerococcus urinae	Aerococcus	Aerococcaceae	Lactobacillales	Bacilli	Firmicutes	Terrabacteria group
IL-DEx_11A_non-UTI	non-UTI	386414	1.43%	264.17	Prevotella timonensis	Prevotella	Prevotellaceae	Bacteroidales	Bacteroidia	Bacteroidetes	FCB group
IL-DEx_11A_non-UTI	non-UTI	184870	1.24%	228.57	Varibaculum cambriense	Varibaculum	Actinomycetaceae	Actinomycetales	Actinobacteria	Actinobacteria	Terrabacteria group
IL-DEx_11A_non-UTI	non-UTI	507750	1.21%	223.89	Peptoniphilus duerdenii	Peptoniphilus	Peptoniphilaceae	Tissierellales	Tissierellia	Firmicutes	Terrabacteria group
IL-DEx_11A_non-UTI	non-UTI	1258	0.99%	183.40	Peptoniphilus asaccharolyticus	Peptoniphilus	Peptoniphilaceae	Tissierellales	Tissierellia	Firmicutes	Terrabacteria group
IL-DEx_11A_non-UTI	non-UTI	1379270	0.69%	126.46	Gemmatimonas phototrophica	Gemmatimonas	Gemmatimonadaceae	Gemmatimonadales	Gemmatimonadetes	Gemmatimonadetes	FCB group
IL-DEx_11A_non-UTI	non-UTI	236753	0.61%	113.35	Fastidiosipila sanguinis	Fastidiosipila	Hungateiclostridiaceae	Clostridiales	Clostridia	Firmicutes	Terrabacteria group
IL-DEx_11A_non-UTI	non-UTI	938278	0.42%	77.75	Casaltella massiliensis	Casaltella	Clostridiales Family XIII. Incertae Sedis	Clostridiales	Clostridia	Firmicutes	Terrabacteria group
IL-DEx_11A_non-UTI	non-UTI	428712	0.42%	76.82	Jonquetella anthropi	Jonquetella	Synergistaceae	Synergistales	Synergistia	Synergistetes	
IL-DEx_11A_non-UTI	non-UTI	1870984	0.41%	76.42	Anaerococcus mediterraneensis	Anaerococcus	Peptoniphilaceae	Tissierellales	Tissierellia	Firmicutes	Terrabacteria group
IL-DEx_11A_non-UTI	non-UTI	361500	0.31%	57.96	Anaerococcus degeneri	Anaerococcus	Peptoniphilaceae	Tissierellales	Tissierellia	Firmicutes	Terrabacteria group
IL-DEx_11A_non-UTI	non-UTI	1230389	0.30%	56.21	Reyranella soli	Reyranella		Rhodospirillales	Alphaproteobacteria	Proteobacteria	
IL-DEx_11A_non-UTI	non-UTI	281920	0.28%	52.46	Porphyromonas uenonis	Porphyromonas	Porphyromonadaceae	Bacteroidales	Bacteroidia	Bacteroidetes	FCB group
IL-DEx_11A_non-UTI	non-UTI	178214	0.28%	51.52	Facklamia hominis	Facklamia	Aerococcaceae	Lactobacillales	Bacilli	Firmicutes	Terrabacteria group
IL-DEx_11A_non-UTI	non-UTI	488447	0.25%	46.11	Burkholderia contaminans	Burkholderia	Burkholderiaceae	Burkholderiales	Betaproteobacteria	Proteobacteria	
IL-DEx_11A_non-UTI	non-UTI	76517	0.25%	45.90	Campylobacter hominis	Campylobacter	Campylobacteraceae	Campylobacterales	Epsilonproteobacteria	Proteobacteria	
IL-DEx_11A_non-UTI	non-UTI	2741	0.24%	44.03	Peptococcus niger	Peptococcus	Peptococcaceae	Clostridiales	Clostridia	Firmicutes	Terrabacteria group
IL-DEx_11A_non-UTI	non-UTI	562	0.22%	40.23	Escherichia coli	Escherichia	Enterobacteriaceae	Enterobacterales	Gammaproteobacteria	Proteobacteria	
IL-DEx_11A_non-UTI	non-UTI	1351	0.20%	36.53	Enterococcus faecalis	Enterococcus	Enterococcaceae	Lactobacillales	Bacilli	Firmicutes	Terrabacteria group
IL-DEx_11A_non-UTI	non-UTI	285106	0.17%	30.97	Tepidimicrobium ferriphilum	Tepidimicrobium	Tissierellaceae	Tissierellales	Tissierellia	Firmicutes	Terrabacteria group
IL-DEx_11A_non-UTI	non-UTI	1261	0.16%	29.04	Peptostreptococcus anaerobius	Peptostreptococcus	Peptostreptococcaceae	Clostridiales	Clostridia	Firmicutes	Terrabacteria group
IL-DEx_11A_non-UTI	non-UTI	1235997	0.15%	27.46	Hydrobacter penzbergensis	Hydrobacter	Chitinophagaceae	Chitinophagales	Chitinophagia	Bacteroidetes	FCB group
IL-DEx_11A_non-UTI	non-UTI	54006	0.14%	26.48	Peptoniphilus ivorii	Peptoniphilus	Peptoniphilaceae	Tissierellales	Tissierellia	Firmicutes	Terrabacteria group
IL-DEx_11A_non-UTI	non-UTI	28127	0.14%	26.23	Prevotella buccalis	Prevotella	Prevotellaceae	Bacteroidales	Bacteroidia	Bacteroidetes	FCB group
IL-DEx_11A_non-UTI	non-UTI	1852374	0.13%	24.36	Ezakiella massiliensis	Ezakiella			Tissierellia	Firmicutes	Terrabacteria group
IL-DEx_11A_non-UTI	non-UTI	34062	0.13%	24.36	Moraxella osloensis	Moraxella	Moraxellaceae	Pseudomonadales	Gammaproteobacteria	Proteobacteria	
IL-DEx_11A_non-UTI	non-UTI	303	0.12%	22.48	Pseudomonas putida	Pseudomonas	Pseudomonadaceae	Pseudomonadales	Gammaproteobacteria	Proteobacteria	
IL-DEx_11A_non-UTI	non-UTI	938289	0.12%	21.55	Levyella massiliensis	Levyella		Clostridiales	Clostridia	Firmicutes	Terrabacteria group
IL-DEx_11A_non-UTI	non-UTI	1776381	0.11%	20.61	Olegusella massiliensis	Olegusella	Coriobacteriaceae	Coriobacteriales	Coriobacteriia	Actinobacteria	Terrabacteria group
IL-DEx_11A_non-UTI	non-UTI	131111	0.11%	20.61	Schaalia turicensis	Schaalia	Actinomycetaceae	Actinomycetales	Actinobacteria	Actinobacteria	Terrabacteria group
IL-DEx_11A_non-UTI	non-UTI	1702287	0.11%	19.67	Negativibacillus massiliensis	Negativicoccus	Veillonellaceae	Veillonellales	Negativicutes	Firmicutes	Terrabacteria group
IL-DEx_11A_non-UTI	non-UTI	1282	0.10%	17.80	Staphylococcus epidermidis	Staphylococcus	Staphylococcaceae	Bacillales	Bacilli	Firmicutes	Terrabacteria group
IL-DEx_11A_non-UTI	non-UTI	573	0.09%	16.91	Klebsiella pneumoniae	Klebsiella	Enterobacteriaceae	Enterobacterales	Gammaproteobacteria	Proteobacteria	
IL-DEx_11A_non-UTI	non-UTI	1816689	0.09%	16.86	Sutterella massiliensis	Sutterella	Sutterellaceae	Burkholderiales	Betaproteobacteria	Proteobacteria	
IL-DEx_11A_non-UTI	non-UTI	1295366	0.09%	15.93	Mesorhizobium soli	Mesorhizobium	Phyllobacteriaceae	Rhizobiales	Alphaproteobacteria	Proteobacteria	
IL-DEx_11A_non-UTI	non-UTI	1393034	0.08%	14.99	Atopobium deltae	Atopobium	Atopobiaceae	Coriobacteriales	Coriobacteriia	Actinobacteria	Terrabacteria group
IL-DEx_11A_non-UTI	non-UTI	54007	0.06%	11.24	Anaerococcus octavius	Anaerococcus	Peptoniphilaceae	Tissierellales	Tissierellia	Firmicutes	Terrabacteria group
IL-DEx_11A_non-UTI	non-UTI	95486	0.05%	10.09	Burkholderia cenocepacia	Burkholderia	Burkholderiaceae	Burkholderiales	Betaproteobacteria	Proteobacteria	
IL-DEx_11A_non-UTI	non-UTI	713059	0.05%	9.37	Candidatus Saccharibacteria bacterium oral taxon 488					Candidatus Saccharibacteria	Bacteria candidate phyla
IL-DEx_11A_non-UTI	non-UTI	unmapped	0.00%	21.00							
IL-DEx_11A_non-UTI	non-UTI	mapped_unclassified	0.00%	1 283.00							
QIAGEN_11A_non-UTI	non-UTI	46507	16.32%	2 966.88	Ezakiella coagulans	Ezakiella			Tissierellia	Firmicutes	Terrabacteria group
QIAGEN_11A_non-UTI	non-UTI	2081703	14.91%	2 709.34	Peptostreptococcaceae bacterium oral taxon 929		Peptostreptococcaceae	Clostridiales	Clostridia	Firmicutes	Terrabacteria group
QIAGEN_11A_non-UTI	non-UTI	33031	10.50%	1 908.62	Peptoniphilus lacrimalis	Peptoniphilus	Peptoniphilaceae	Tissierellales	Tissierellia	Firmicutes	Terrabacteria group
QIAGEN_11A_non-UTI	non-UTI	501496	8.95%	1 626.73	Porphyromonas bennonis	Porphyromonas	Porphyromonadaceae	Bacteroidales	Bacteroidia	Bacteroidetes	FCB group
QIAGEN_11A_non-UTI	non-UTI	827	8.21%	1 491.88	Campylobacter ureolyticus	Campylobacter	Campylobacteraceae	Campylobacterales	Epsilonproteobacteria	Proteobacteria	
QIAGEN_11A_non-UTI	non-UTI	33032	6.79%	1 233.16	Anaerococcus lactolyticus	Anaerococcus	Peptoniphilaceae	Tissierellales	Tissierellia	Firmicutes	Terrabacteria group
QIAGEN_11A_non-UTI	non-UTI	54005	4.62%	839.12	Peptoniphilus harei	Peptoniphilus	Peptoniphilaceae	Tissierellales	Tissierellia	Firmicutes	Terrabacteria group
QIAGEN_11A_non-UTI	non-UTI	1465756	4.39%	796.97	Peptoniphilus grossensis	Peptoniphilus	Peptoniphilaceae	Tissierellales	Tissierellia	Firmicutes	Terrabacteria group
QIAGEN_11A_non-UTI	non-UTI	33012	2.76%	501.97	Propionimicrobium lymphophilum	Propionimicrobium	Propionibacteriaceae	Propionibacteriales	Actinobacteria	Actinobacteria	Terrabacteria group
QIAGEN_11A_non-UTI	non-UTI	507750	2.27%	412.07	Peptoniphilus duerdenii	Peptoniphilus	Peptoniphilaceae	Tissierellales	Tissierellia	Firmicutes	Terrabacteria group
QIAGEN_11A_non-UTI	non-UTI	308994	1.91%	346.51	Dialister propionicifaciens	Dialister	Veillonellaceae	Veillonellales	Negativicutes	Firmicutes	Terrabacteria group
QIAGEN_11A_non-UTI	non-UTI	1260	1.89%	343.70	Finegoldia magna	Finegoldia	Peptoniphilaceae	Tissierellales	Tissierellia	Firmicutes	Terrabacteria group
QIAGEN_11A_non-UTI	non-UTI	1258	1.87%	339.36	Peptoniphilus asaccharolyticus	Peptoniphilus	Peptoniphilaceae	Tissierellales	Tissierellia	Firmicutes	Terrabacteria group
QIAGEN_11A_non-UTI	non-UTI	1376	1.83%	333.40	Aerococcus urinae	Aerococcus	Aerococcaceae	Lactobacillales	Bacilli	Firmicutes	Terrabacteria group
QIAGEN_11A_non-UTI	non-UTI	2051	1.72%	312.80	Mobiluncus curtisii	Mobiluncus	Actinomycetaceae	Actinomycetales	Actinobacteria	Actinobacteria	Terrabacteria group
QIAGEN_11A_non-UTI	non-UTI	386414	1.49%	271.59	Prevotella timonensis	Prevotella	Prevotellaceae	Bacteroidales	Bacteroidia	Bacteroidetes	FCB group
QIAGEN_11A_non-UTI	non-UTI	76517	1.49%	270.65	Campylobacter hominis	Campylobacter	Campylobacteraceae	Campylobacterales	Epsilonproteobacteria	Proteobacteria	
QIAGEN_11A_non-UTI	non-UTI	938278	1.04%	188.24	Casaltella massiliensis	Casaltella	Clostridiales Family XIII. Incertae Sedis	Clostridiales	Clostridia	Firmicutes	Terrabacteria group
QIAGEN_11A_non-UTI	non-UTI	411577	1.00%	181.10	Anaerococcus murdochii	Anaerococcus	Peptoniphilaceae	Tissierellales	Tissierellia	Firmicutes	Terrabacteria group
QIAGEN_11A_non-UTI	non-UTI	755172	0.75%	135.89	Peptoniphilus coxii	Peptoniphilus	Peptoniphilaceae	Tissierellales	Tissierellia	Firmicutes	Terrabacteria group
QIAGEN_11A_non-UTI	non-UTI	1870984	0.67%	121.83	Anaerococcus mediterraneensis	Anaerococcus	Peptoniphilaceae	Tissierellales	Tissierellia	Firmicutes	Terrabacteria group
QIAGEN_11A_non-UTI	non-UTI	236753	0.55%	99.27	Fastidiosipila sanguinis	Fastidiosipila	Hungateiclostridiaceae	Clostridiales	Clostridia	Firmicutes	Terrabacteria group
QIAGEN_11A_non-UTI	non-UTI	184870	0.48%	87.10	Varibaculum cambriense	Varibaculum	Actinomycetaceae	Actinomycetales	Actinobacteria	Actinobacteria	Terrabacteria group
QIAGEN_11A_non-UTI	non-UTI	178214	0.47%	86.16	Facklamia hominis	Facklamia	Aerococcaceae	Lactobacillales	Bacilli	Firmicutes	Terrabacteria group
QIAGEN_11A_non-UTI	non-UTI	2572088	0.37%	66.49	Candidatus Saccharibacteria bacterium oral taxon 957					Candidatus Saccharibacteria	Bacteria candidate phyla
QIAGEN_11A_non-UTI	non-UTI	2741	0.34%	61.81	Peptococcus niger	Peptococcus	Peptococcaceae	Clostridiales	Clostridia	Firmicutes	Terrabacteria group
QIAGEN_11A_non-UTI	non-UTI	281920	0.30%	55.25	Porphyromonas uenonis	Porphyromonas	Porphyromonadaceae	Bacteroidales	Bacteroidia	Bacteroidetes	FCB group
QIAGEN_11A_non-UTI	non-UTI	28127	0.29%	53.38	Prevotella buccalis	Prevotella	Prevotellaceae	Bacteroidales	Bacteroidia	Bacteroidetes	FCB group
QIAGEN_11A_non-UTI	non-UTI	1393034	0.21%	37.46	Atopobium deltae	Atopobium	Atopobiaceae	Coriobacteriales	Coriobacteriia	Actinobacteria	Terrabacteria group
QIAGEN_11A_non-UTI	non-UTI	319644	0.20%	35.59	Saccharofermentans acetigenes	Saccharofermentan	Hungateiclostridiaceae	Clostridiales	Clostridia	Firmicutes	Terrabacteria group
QIAGEN_11A_non-UTI	non-UTI	54006	0.19%	34.84	Peptoniphilus ivorii	Peptoniphilus	Peptoniphilaceae	Tissierellales	Tissierellia	Firmicutes	Terrabacteria group
QIAGEN_11A_non-UTI	non-UTI	361500	0.14%	26.00	Anaerococcus degeneri	Anaerococcus	Peptoniphilaceae	Tissierellales	Tissierellia	Firmicutes	Terrabacteria group
QIAGEN_11A_non-UTI	non-UTI	285106	0.14%	24.65	Tepidimicrobium ferriphilum	Tepidimicrobium	Tissierellaceae	Tissierellales	Tissierellia	Firmicutes	Terrabacteria group
QIAGEN_11A_non-UTI	non-UTI	404403	0.12%	22.48	Howardella ureilytica	Howardella		Clostridiales	Clostridia	Firmicutes	Terrabacteria group
QIAGEN_11A_non-UTI	non-UTI	1703337	0.12%	21.54	Prevotella colorans	Prevotella	Prevotellaceae	Bacteroidales	Bacteroidia	Bacteroidetes	FCB group
QIAGEN_11A_non-UTI	non-UTI	29524	0.12%	21.54	Porphyromonas circumdentaria	Porphyromonas	Porphyromonadaceae	Bacteroidales	Bacteroidia	Bacteroidetes	FCB group
QIAGEN_11A_non-UTI	non-UTI	562	0.11%	19.67	Escherichia coli	Escherichia	Enterobacteriaceae	Enterobacterales	Gammaproteobacteria	Proteobacteria	
QIAGEN_11A_non-UTI	non-UTI	1852374	0.10%	18.73	Ezakiella massiliensis	Ezakiella			Tissierellia	Firmicutes	Terrabacteria group
QIAGEN_11A_non-UTI	non-UTI	428712	0.09%	16.86	Jonquetella anthropi	Jonquetella	Synergistaceae	Synergistales	Synergistia	Synergistetes	
QIAGEN_11A_non-UTI	non-UTI	2576606	0.09%	16.86	Duncaniella sp. B8	Duncaniella	Muribaculaceae	Bacteroidales	Bacteroidia	Bacteroidetes	FCB group
QIAGEN_11A_non-UTI	non-UTI	1287640	0.08%	14.98	Anaerococcus obesiensis	Anaerococcus	Peptoniphilaceae	Tissierellales	Tissierellia	Firmicutes	Terrabacteria group
QIAGEN_11A_non-UTI	non-UTI	28264	0.06%	11.24	Arcanobacterium haemolyticum	Arcanobacterium	Actinomycetaceae	Actinomycetales	Actinobacteria	Actinobacteria	Terrabacteria group
QIAGEN_11A_non-UTI	non-UTI	242750	0.06%	10.30	Prevotella bergensis	Prevotella	Prevotellaceae	Bacteroidales	Bacteroidia	Bacteroidetes	FCB group
QIAGEN_11A_non-UTI	non-UTI	unmapped	0.00%	142.00							
QIAGEN_11A_non-UTI	non-UTI	mapped_unclassified	0.00%	1 351.00							
Promega_11A_non-UTI	non-UTI	46507	15.93%	2 763.24	Ezakiella coagulans	Ezakiella			Tissierellia	Firmicutes	Terrabacteria group
Promega_11A_non-UTI	non-UTI	2081703	12.73%	2 208.56	Peptostreptococcaceae bacterium oral taxon 929		Peptostreptococcaceae	Clostridiales	Clostridia	Firmicutes	Terrabacteria group
Promega_11A_non-UTI	non-UTI	33031	11.56%	2 005.18	Peptoniphilus lacrimalis	Peptoniphilus	Peptoniphilaceae	Tissierellales	Tissierellia	Firmicutes	Terrabacteria group
Promega_11A_non-UTI	non-UTI	501496	9.21%	1 596.56	Porphyromonas bennonis	Porphyromonas	Porphyromonadaceae	Bacteroidales	Bacteroidia	Bacteroidetes	FCB group
Promega_11A_non-UTI	non-UTI	54005	6.07%	1 053.05	Peptoniphilus harei	Peptoniphilus	Peptoniphilaceae	Tissierellales	Tissierellia	Firmicutes	Terrabacteria group
Promega_11A_non-UTI	non-UTI	33012	5.69%	987.34	Propionimicrobium lymphophilum	Propionimicrobium	Propionibacteriaceae	Propionibacteriales	Actinobacteria	Actinobacteria	Terrabacteria group
Promega_11A_non-UTI	non-UTI	827	5.37%	931.87	Campylobacter ureolyticus	Campylobacter	Campylobacteraceae	Campylobacterales	Epsilonproteobacteria	Proteobacteria	
Promega_11A_non-UTI	non-UTI	33032	5.01%	869.27	Anaerococcus lactolyticus	Anaerococcus	Peptoniphilaceae	Tissierellales	Tissierellia	Firmicutes	Terrabacteria group
Promega_11A_non-UTI	non-UTI	1465756	4.66%	808.84	Peptoniphilus grossensis	Peptoniphilus	Peptoniphilaceae	Tissierellales	Tissierellia	Firmicutes	Terrabacteria group
Promega_11A_non-UTI	non-UTI	308994	2.75%	477.03	Dialister propionicifaciens	Dialister	Veillonellaceae	Veillonellales	Negativicutes	Firmicutes	Terrabacteria group
Promega_11A_non-UTI	non-UTI	2051	2.74%	475.18	Mobiluncus curtisii	Mobiluncus	Actinomycetaceae	Actinomycetales	Actinobacteria	Actinobacteria	Terrabacteria group
Promega_11A_non-UTI	non-UTI	507750	2.46%	426.18	Peptoniphilus duerdenii	Peptoniphilus	Peptoniphilaceae	Tissierellales	Tissierellia	Firmicutes	Terrabacteria group
Promega_11A_non-UTI	non-UTI	1376	2.19%	379.96	Aerococcus urinae	Aerococcus	Aerococcaceae	Lactobacillales	Bacilli	Firmicutes	Terrabacteria group
Promega_11A_non-UTI	non-UTI	1258	1.54%	266.60	Peptoniphilus asaccharolyticus	Peptoniphilus	Peptoniphilaceae	Tissierellales	Tissierellia	Firmicutes	Terrabacteria group
Promega_11A_non-UTI	non-UTI	1260	1.43%	247.76	Finegoldia magna	Finegoldia	Peptoniphilaceae	Tissierellales	Tissierellia	Firmicutes	Terrabacteria group
Promega_11A_non-UTI	non-UTI	938278	1.34%	232.97	Casaltella massiliensis	Casaltella	Clostridiales Family XIII. Incertae Sedis	Clostridiales	Clostridia	Firmicutes	Terrabacteria group
Promega_11A_non-UTI	non-UTI	386414	1.26%	218.18	Prevotella timonensis	Prevotella	Prevotellaceae	Bacteroidales	Bacteroidia	Bacteroidetes	FCB group
Promega_11A_non-UTI	non-UTI	184870	0.97%	168.25	Varibaculum cambriense	Varibaculum	Actinomycetaceae	Actinomycetales	Actinobacteria	Actinobacteria	Terrabacteria group
Promega_11A_non-UTI	non-UTI	76517	0.79%	136.82	Campylobacter hominis	Campylobacter	Campylobacteraceae	Campylobacterales	Epsilonproteobacteria	Proteobacteria	
Promega_11A_non-UTI	non-UTI	411577	0.69%	120.50	Anaerococcus murdochii	Anaerococcus	Peptoniphilaceae	Tissierellales	Tissierellia	Firmicutes	Terrabacteria group
Promega_11A_non-UTI	non-UTI	2572088	0.65%	112.33	Candidatus Saccharibacteria bacterium oral taxon 957					Candidatus Saccharibacteria	Bacteria candidate phyla
Promega_11A_non-UTI	non-UTI	178214	0.55%	95.22	Facklamia hominis	Facklamia	Aerococcaceae	Lactobacillales	Bacilli	Firmicutes	Terrabacteria group
Promega_11A_non-UTI	non-UTI	755172	0.50%	86.52	Peptoniphilus coxii	Peptoniphilus	Peptoniphilaceae	Tissierellales	Tissierellia	Firmicutes	Terrabacteria group
Promega_11A_non-UTI	non-UTI	281920	0.37%	64.71	Porphyromonas uenonis	Porphyromonas	Porphyromonadaceae	Bacteroidales	Bacteroidia	Bacteroidetes	FCB group
Promega_11A_non-UTI	non-UTI	1870984	0.37%	63.61	Anaerococcus mediterraneensis	Anaerococcus	Peptoniphilaceae	Tissierellales	Tissierellia	Firmicutes	Terrabacteria group
Promega_11A_non-UTI	non-UTI	236753	0.35%	61.02	Fastidiosipila sanguinis	Fastidiosipila	Hungateiclostridiaceae	Clostridiales	Clostridia	Firmicutes	Terrabacteria group
Promega_11A_non-UTI	non-UTI	319644	0.25%	43.45	Saccharofermentans acetigenes	Saccharofermentan	Hungateiclostridiaceae	Clostridiales	Clostridia	Firmicutes	Terrabacteria group
Promega_11A_non-UTI	non-UTI	28127	0.24%	41.60	Prevotella buccalis	Prevotella	Prevotellaceae	Bacteroidales	Bacteroidia	Bacteroidetes	FCB group
Promega_11A_non-UTI	non-UTI	216816	0.22%	37.90	Bifidobacterium longum	Bifidobacterium	Bifidobacteriaceae	Bifidobacteriales	Actinobacteria	Actinobacteria	Terrabacteria group
Promega_11A_non-UTI	non-UTI	428712	0.21%	36.05	Jonquetella anthropi	Jonquetella	Synergistaceae	Synergistales	Synergistia	Synergistetes	
Promega_11A_non-UTI	non-UTI	562	0.18%	30.51	Escherichia coli	Escherichia	Enterobacteriaceae	Enterobacterales	Gammaproteobacteria	Proteobacteria	
Promega_11A_non-UTI	non-UTI	2741	0.17%	28.66	Peptococcus niger	Peptococcus	Peptococcaceae	Clostridiales	Clostridia	Firmicutes	Terrabacteria group
Promega_11A_non-UTI	non-UTI	54006	0.16%	27.70	Peptoniphilus ivorii	Peptoniphilus	Peptoniphilaceae	Tissierellales	Tissierellia	Firmicutes	Terrabacteria group
Promega_11A_non-UTI	non-UTI	131111	0.15%	25.89	Schaalia turicensis	Schaalia	Actinomycetaceae	Actinomycetales	Actinobacteria	Actinobacteria	Terrabacteria group
Promega_11A_non-UTI	non-UTI	28264	0.13%	23.11	Arcanobacterium haemolyticum	Arcanobacterium	Actinomycetaceae	Actinomycetales	Actinobacteria	Actinobacteria	Terrabacteria group
Promega_11A_non-UTI	non-UTI	285106	0.13%	22.25	Tepidimicrobium ferriphilum	Tepidimicrobium	Tissierellaceae	Tissierellales	Tissierellia	Firmicutes	Terrabacteria group
Promega_11A_non-UTI	non-UTI	29524	0.10%	17.57	Porphyromonas circumdentaria	Porphyromonas	Porphyromonadaceae	Bacteroidales	Bacteroidia	Bacteroidetes	FCB group
Promega_11A_non-UTI	non-UTI	1703337	0.10%	17.56	Prevotella colorans	Prevotella	Prevotellaceae	Bacteroidales	Bacteroidia	Bacteroidetes	FCB group
Promega_11A_non-UTI	non-UTI	1852374	0.10%	16.64	Ezakiella massiliensis	Ezakiella			Tissierellia	Firmicutes	Terrabacteria group
Promega_11A_non-UTI	non-UTI	1351	0.10%	16.64	Enterococcus faecalis	Enterococcus	Enterococcaceae	Lactobacillales	Bacilli	Firmicutes	Terrabacteria group
Promega_11A_non-UTI	non-UTI	1393034	0.10%	16.64	Atopobium deltae	Atopobium	Atopobiaceae	Coriobacteriales	Coriobacteriia	Actinobacteria	Terrabacteria group
Promega_11A_non-UTI	non-UTI	361500	0.09%	15.32	Anaerococcus degeneri	Anaerococcus	Peptoniphilaceae	Tissierellales	Tissierellia	Firmicutes	Terrabacteria group
Promega_11A_non-UTI	non-UTI	33034	0.09%	14.79	Anaerococcus prevotii	Anaerococcus	Peptoniphilaceae	Tissierellales	Tissierellia	Firmicutes	Terrabacteria group
Promega_11A_non-UTI	non-UTI	2490633	0.07%	12.02	Streptococcus periodonticum	Streptococcus	Streptococcaceae	Lactobacillales	Bacilli	Firmicutes	Terrabacteria group
Promega_11A_non-UTI	non-UTI	2572087	0.07%	11.55	Candidatus Saccharibacteria bacterium oral taxon 955					Candidatus Saccharibacteria	Bacteria candidate phyla
Promega_11A_non-UTI	non-UTI	938289	0.06%	11.09	Levyella massiliensis	Levyella		Clostridiales	Clostridia	Firmicutes	Terrabacteria group
Promega_11A_non-UTI	non-UTI	322095	0.06%	10.17	Porphyromonas somerae	Porphyromonas	Porphyromonadaceae	Bacteroidales	Bacteroidia	Bacteroidetes	FCB group
Promega_11A_non-UTI	non-UTI	1528099	0.06%	10.17	Lawsonella clevelandensis	Lawsonella		Corynebacteriales	Actinobacteria	Actinobacteria	Terrabacteria group
Promega_11A_non-UTI	non-UTI	unmapped	0.00%	501.00							
Promega_11A_non-UTI	non-UTI	mapped_unclassified	0.00%	1 521.00							
PC_11A_non-UTI	non-UTI	46507	18.97%	2 424.97	Ezakiella coagulans	Ezakiella			Tissierellia	Firmicutes	Terrabacteria group
PC_11A_non-UTI	non-UTI	2081703	15.64%	1 998.92	Peptostreptococcaceae bacterium oral taxon 929		Peptostreptococcaceae	Clostridiales	Clostridia	Firmicutes	Terrabacteria group
PC_11A_non-UTI	non-UTI	501496	9.00%	1 149.74	Porphyromonas bennonis	Porphyromonas	Porphyromonadaceae	Bacteroidales	Bacteroidia	Bacteroidetes	FCB group
PC_11A_non-UTI	non-UTI	33031	8.37%	1 069.46	Peptoniphilus lacrimalis	Peptoniphilus	Peptoniphilaceae	Tissierellales	Tissierellia	Firmicutes	Terrabacteria group
PC_11A_non-UTI	non-UTI	827	8.12%	1 037.16	Campylobacter ureolyticus	Campylobacter	Campylobacteraceae	Campylobacterales	Epsilonproteobacteria	Proteobacteria	
PC_11A_non-UTI	non-UTI	1465756	7.81%	997.49	Peptoniphilus grossensis	Peptoniphilus	Peptoniphilaceae	Tissierellales	Tissierellia	Firmicutes	Terrabacteria group
PC_11A_non-UTI	non-UTI	54005	5.62%	718.82	Peptoniphilus harei	Peptoniphilus	Peptoniphilaceae	Tissierellales	Tissierellia	Firmicutes	Terrabacteria group
PC_11A_non-UTI	non-UTI	308994	4.55%	581.33	Dialister propionicifaciens	Dialister	Veillonellaceae	Veillonellales	Negativicutes	Firmicutes	Terrabacteria group
PC_11A_non-UTI	non-UTI	33032	3.42%	437.25	Anaerococcus lactolyticus	Anaerococcus	Peptoniphilaceae	Tissierellales	Tissierellia	Firmicutes	Terrabacteria group
PC_11A_non-UTI	non-UTI	507750	3.13%	399.55	Peptoniphilus duerdenii	Peptoniphilus	Peptoniphilaceae	Tissierellales	Tissierellia	Firmicutes	Terrabacteria group
PC_11A_non-UTI	non-UTI	386414	2.35%	300.81	Prevotella timonensis	Prevotella	Prevotellaceae	Bacteroidales	Bacteroidia	Bacteroidetes	FCB group
PC_11A_non-UTI	non-UTI	1258	1.45%	185.19	Peptoniphilus asaccharolyticus	Peptoniphilus	Peptoniphilaceae	Tissierellales	Tissierellia	Firmicutes	Terrabacteria group
PC_11A_non-UTI	non-UTI	938278	1.26%	160.56	Casaltella massiliensis	Casaltella	Clostridiales Family XIII. Incertae Sedis	Clostridiales	Clostridia	Firmicutes	Terrabacteria group
PC_11A_non-UTI	non-UTI	76517	1.08%	138.41	Campylobacter hominis	Campylobacter	Campylobacteraceae	Campylobacterales	Epsilonproteobacteria	Proteobacteria	
PC_11A_non-UTI	non-UTI	755172	1.06%	136.02	Peptoniphilus coxii	Peptoniphilus	Peptoniphilaceae	Tissierellales	Tissierellia	Firmicutes	Terrabacteria group

PC_11A_non-UTI	non-UTI	1260	0.92%	117.86	Finegoldia magna	Finegoldia	Peptoniphilaceae	Tissierellales	Tissierellia	Firmicutes	Terrabacteria group
PC_11A_non-UTI	non-UTI	33012	0.85%	108.88	Propionimicrobium lymphophilum	Propionimicrobium	Propionibacteriaceae	Propionibacteriales	Actinobacteria	Actinobacteria	Terrabacteria group
PC_11A_non-UTI	non-UTI	281920	0.69%	88.58	Porphyromonas uenonis	Porphyromonas	Porphyromonadaceae	Bacteroidales	Bacteroidia	Bacteroidetes	FCB group
PC_11A_non-UTI	non-UTI	2051	0.66%	84.89	Mobiluncus curtisii	Mobiluncus	Actinomycetaceae	Actinomycetales	Actinobacteria	Actinobacteria	Terrabacteria group
PC_11A_non-UTI	non-UTI	319644	0.48%	61.82	Saccharofermentans acetigenes	Saccharofermentans	Hungateiclostridiaceae	Clostridiales	Clostridia	Firmicutes	Terrabacteria group
PC_11A_non-UTI	non-UTI	236753	0.46%	59.06	Fastidiosipila sanguinis	Fastidiosipila	Hungateiclostridiaceae	Clostridiales	Clostridia	Firmicutes	Terrabacteria group
PC_11A_non-UTI	non-UTI	562	0.33%	42.45	Escherichia coli	Escherichia	Enterobacteriaceae	Enterobacterales	Gammaproteobacteria	Proteobacteria	
PC_11A_non-UTI	non-UTI	2572088	0.31%	39.68	Candidatus Saccharibacteria bacterium oral taxon 957					Candidatus Saccharibacteria	Bacteria candidate phyla
PC_11A_non-UTI	non-UTI	1376	0.30%	37.83	Aerococcus urinae	Aerococcus	Aerococcaceae	Lactobacillales	Bacilli	Firmicutes	Terrabacteria group
PC_11A_non-UTI	non-UTI	1852374	0.24%	30.45	Ezakiella massiliensis	Ezakiella			Tissierellia	Firmicutes	Terrabacteria group
PC_11A_non-UTI	non-UTI	2578113	0.23%	29.53	Bradyrhizobium sp. KBS0725	Bradyrhizobium	Bradyrhizobiaceae	Rhizobiales	Alphaproteobacteria	Proteobacteria	
PC_11A_non-UTI	non-UTI	178214	0.22%	28.61	Facklamia hominis	Facklamia	Aerococcaceae	Lactobacillales	Bacilli	Firmicutes	Terrabacteria group
PC_11A_non-UTI	non-UTI	28127	0.20%	25.84	Prevotella buccalis	Prevotella	Prevotellaceae	Bacteroidales	Bacteroidia	Bacteroidetes	FCB group
PC_11A_non-UTI	non-UTI	287	0.20%	25.84	Pseudomonas aeruginosa	Pseudomonas	Pseudomonadaceae	Pseudomonadales	Gammaproteobacteria	Proteobacteria	
PC_11A_non-UTI	non-UTI	54006	0.20%	25.25	Peptoniphilus ivorii	Peptoniphilus	Peptoniphilaceae	Tissierellales	Tissierellia	Firmicutes	Terrabacteria group
PC_11A_non-UTI	non-UTI	428712	0.18%	23.07	Jonquetella anthropi	Jonquetella	Synergistaceae	Synergistales	Synergistia	Synergistetes	
PC_11A_non-UTI	non-UTI	1295366	0.17%	22.15	Mesorhizobium soli	Mesorhizobium	Phyllobacteriaceae	Rhizobiales	Alphaproteobacteria	Proteobacteria	
PC_11A_non-UTI	non-UTI	674703	0.17%	21.22	Rhodoplanes sp. Z2-YC6860	Rhodoplanes	Hyphomicrobiaceae	Rhizobiales	Alphaproteobacteria	Proteobacteria	
PC_11A_non-UTI	non-UTI	411577	0.17%	21.10	Anaerococcus murdochii	Anaerococcus	Peptoniphilaceae	Tissierellales	Tissierellia	Firmicutes	Terrabacteria group
PC_11A_non-UTI	non-UTI	1162689	0.14%	18.45	Sediminibacterium lactis	Sediminibacterium	Chitinophagaceae	Chitinophagales	Chitinophagia	Bacteroidetes	FCB group
PC_11A_non-UTI	non-UTI	1870984	0.14%	17.78	Anaerococcus mediterraneensis	Anaerococcus	Peptoniphilaceae	Tissierellales	Tissierellia	Firmicutes	Terrabacteria group
PC_11A_non-UTI	non-UTI	285106	0.12%	15.26	Tepidimicrobium ferriphilum	Tepidimicrobium	Tissierellaceae	Tissierellales	Tissierellia	Firmicutes	Terrabacteria group
PC_11A_non-UTI	non-UTI	29524	0.12%	14.76	Porphyromonas circumdentaria	Porphyromonas	Porphyromonadaceae	Bacteroidales	Bacteroidia	Bacteroidetes	FCB group
PC_11A_non-UTI	non-UTI	1805714	0.10%	12.92	Christensenella massiliensis	Christensenella	Christensenellaceae	Clostridiales	Clostridia	Firmicutes	Terrabacteria group
PC_11A_non-UTI	non-UTI	1747	0.10%	12.92	Cutibacterium acnes	Cutibacterium	Propionibacteriaceae	Propionibacteriales	Actinobacteria	Actinobacteria	Terrabacteria group
PC_11A_non-UTI	non-UTI	2741	0.10%	12.92	Peptococcus niger	Peptococcus	Peptococcaceae	Clostridiales	Clostridia	Firmicutes	Terrabacteria group
PC_11A_non-UTI	non-UTI	184870	0.09%	12.00	Varibaculum cambriense	Varibaculum	Actinomycetaceae	Actinomycetales	Actinobacteria	Actinobacteria	Terrabacteria group
PC_11A_non-UTI	non-UTI	242750	0.09%	12.00	Prevotella bergensis	Prevotella	Prevotellaceae	Bacteroidales	Bacteroidia	Bacteroidetes	FCB group
PC_11A_non-UTI	non-UTI	288426	0.09%	11.07	Paracoccus marinus	Paracoccus	Rhodobacteraceae	Rhodobacterales	Alphaproteobacteria	Proteobacteria	
PC_11A_non-UTI	non-UTI	1703337	0.08%	10.15	Prevotella colorans	Prevotella	Prevotellaceae	Bacteroidales	Bacteroidia	Bacteroidetes	FCB group
PC_11A_non-UTI	non-UTI	unmapped	0.00%	3 342.00							
PC_11A_non-UTI	non-UTI	mapped_unclassified	0.00%	1 147.00							
IL-DEx_12A_non-UTI	non-UTI	1351	99.78%	19 476.09	Enterococcus faecalis	Enterococcus	Enterococcaceae	Lactobacillales	Bacilli	Firmicutes	Terrabacteria group
IL-DEx_12A_non-UTI	non-UTI	1283	0.09%	17.96	Staphylococcus haemolyticus	Staphylococcus	Staphylococcaceae	Bacillales	Bacilli	Firmicutes	Terrabacteria group
IL-DEx_12A_non-UTI	non-UTI	669455	0.08%	14.97	Sediminibacterium magnilacihabitans	Sediminibacterium	Chitinophagaceae	Chitinophagales	Chitinophagia	Bacteroidetes	FCB group
IL-DEx_12A_non-UTI	non-UTI	562	0.06%	10.98	Escherichia coli	Escherichia	Enterobacteriaceae	Enterobacterales	Gammaproteobacteria	Proteobacteria	
IL-DEx_12A_non-UTI	non-UTI	unmapped	0.00%	132.00							
IL-DEx_12A_non-UTI	non-UTI	mapped_unclassified	0.00%	57.00							
QIAGEN_12A_non-UTI	non-UTI	1351	99.76%	17 089.10	Enterococcus faecalis	Enterococcus	Enterococcaceae	Lactobacillales	Bacilli	Firmicutes	Terrabacteria group
QIAGEN_12A_non-UTI	non-UTI	562	0.17%	28.93	Escherichia coli	Escherichia	Enterobacteriaceae	Enterobacterales	Gammaproteobacteria	Proteobacteria	
QIAGEN_12A_non-UTI	non-UTI	1282	0.08%	12.97	Staphylococcus epidermidis	Staphylococcus	Staphylococcaceae	Bacillales	Bacilli	Firmicutes	Terrabacteria group
QIAGEN_12A_non-UTI	non-UTI	unmapped	0.00%	1 669.00							
QIAGEN_12A_non-UTI	non-UTI	mapped_unclassified	0.00%	92.00							
Promega_12A_non-UTI	non-UTI	1351	99.73%	19 454.14	Enterococcus faecalis	Enterococcus	Enterococcaceae	Lactobacillales	Bacilli	Firmicutes	Terrabacteria group
Promega_12A_non-UTI	non-UTI	1282	0.10%	19.95	Staphylococcus epidermidis	Staphylococcus	Staphylococcaceae	Bacillales	Bacilli	Firmicutes	Terrabacteria group
Promega_12A_non-UTI	non-UTI	1283	0.09%	16.96	Staphylococcus haemolyticus	Staphylococcus	Staphylococcaceae	Bacillales	Bacilli	Firmicutes	Terrabacteria group
Promega_12A_non-UTI	non-UTI	562	0.08%	15.96	Escherichia coli	Escherichia	Enterobacteriaceae	Enterobacterales	Gammaproteobacteria	Proteobacteria	
Promega_12A_non-UTI	non-UTI	unmapped	0.00%	37.00							
Promega_12A_non-UTI	non-UTI	mapped_unclassified	0.00%	58.00							
PC_12A_non-UTI	non-UTI	1351	99.67%	18 563.21	Enterococcus faecalis	Enterococcus	Enterococcaceae	Lactobacillales	Bacilli	Firmicutes	Terrabacteria group
PC_12A_non-UTI	non-UTI	1776391	0.18%	32.89	Romboutsia timonensis	Romboutsia	Peptostreptococcaceae	Clostridiales	Clostridia	Firmicutes	Terrabacteria group
PC_12A_non-UTI	non-UTI	562	0.16%	28.90	Escherichia coli	Escherichia	Enterobacteriaceae	Enterobacterales	Gammaproteobacteria	Proteobacteria	
PC_12A_non-UTI	non-UTI	unmapped	0.00%	195.00							
PC_12A_non-UTI	non-UTI	mapped_unclassified	0.00%	100.00							
IL-DEx_13A_non-UTI	non-UTI	562	80.29%	58.61	Escherichia coli	Escherichia	Enterobacteriaceae	Enterobacterales	Gammaproteobacteria	Proteobacteria	
IL-DEx_13A_non-UTI	non-UTI	1351	6.85%	5.00	Enterococcus faecalis	Enterococcus	Enterococcaceae	Lactobacillales	Bacilli	Firmicutes	Terrabacteria group
IL-DEx_13A_non-UTI	non-UTI	573	6.01%	4.39	Klebsiella pneumoniae	Klebsiella	Enterobacteriaceae	Enterobacterales	Gammaproteobacteria	Proteobacteria	
IL-DEx_13A_non-UTI	non-UTI	545	4.11%	3.00	Citrobacter koseri	Citrobacter	Enterobacteriaceae	Enterobacterales	Gammaproteobacteria	Proteobacteria	
IL-DEx_13A_non-UTI	non-UTI	1870984	1.37%	1.00	Anaerococcus mediterraneensis	Anaerococcus	Peptoniphilaceae	Tissierellales	Tissierellia	Firmicutes	Terrabacteria group
IL-DEx_13A_non-UTI	non-UTI	1311	1.37%	1.00	Streptococcus agalactiae	Streptococcus	Streptococcaceae	Lactobacillales	Bacilli	Firmicutes	Terrabacteria group
IL-DEx_13A_non-UTI	non-UTI	unmapped	0.00%	4.00							
IL-DEx_13A_non-UTI	non-UTI	mapped_unclassified	0.00%	2.00							
QIAGEN_13B_non-UTI	non-UTI	562	23.77%	77.49	Escherichia coli	Escherichia	Enterobacteriaceae	Enterobacterales	Gammaproteobacteria	Proteobacteria	
QIAGEN_13B_non-UTI	non-UTI	28037	21.76%	70.95	Streptococcus mitis	Streptococcus	Streptococcaceae	Lactobacillales	Bacilli	Firmicutes	Terrabacteria group
QIAGEN_13B_non-UTI	non-UTI	34062	17.94%	58.49	Moraxella osloensis	Moraxella	Moraxellaceae	Pseudomonadales	Gammaproteobacteria	Proteobacteria	
QIAGEN_13B_non-UTI	non-UTI	1501332	13.00%	42.38	Oribacterium asaccharolyticum	Oribacterium	Lachnospiraceae	Clostridiales	Clostridia	Firmicutes	Terrabacteria group
QIAGEN_13B_non-UTI	non-UTI	55148	4.45%	14.51	Deinococcus proteolyticus	Deinococcus	Deinococcaceae	Deinococcales	Deinococci	Deinococcus-Thermus	Terrabacteria group
QIAGEN_13B_non-UTI	non-UTI	1351	3.53%	11.51	Enterococcus faecalis	Enterococcus	Enterococcaceae	Lactobacillales	Bacilli	Firmicutes	Terrabacteria group
QIAGEN_13B_non-UTI	non-UTI	81499	3.53%	11.51	Friedmanniella spumicola	Friedmanniella	Nocardioidaceae	Propionibacteriales	Actinobacteria	Actinobacteria	Terrabacteria group
QIAGEN_13B_non-UTI	non-UTI	1501329	2.75%	8.97	Oribacterium parvum	Oribacterium	Lachnospiraceae	Clostridiales	Clostridia	Firmicutes	Terrabacteria group
QIAGEN_13B_non-UTI	non-UTI	573	1.52%	4.97	Klebsiella pneumoniae	Klebsiella	Enterobacteriaceae	Enterobacterales	Gammaproteobacteria	Proteobacteria	
QIAGEN_13B_non-UTI	non-UTI	545	1.18%	3.84	Citrobacter koseri	Citrobacter	Enterobacteriaceae	Enterobacterales	Gammaproteobacteria	Proteobacteria	
QIAGEN_13B_non-UTI	non-UTI	287	1.18%	3.84	Pseudomonas aeruginosa	Pseudomonas	Pseudomonadaceae	Pseudomonadales	Gammaproteobacteria	Proteobacteria	
QIAGEN_13B_non-UTI	non-UTI	156978	0.59%	1.92	Corynebacterium imitans	Corynebacterium	Corynebacteriaceae	Corynebacteriales	Actinobacteria	Actinobacteria	Terrabacteria group
QIAGEN_13B_non-UTI	non-UTI	1261	0.59%	1.92	Peptostreptococcus anaerobius	Peptostreptococcus	Peptostreptococcaceae	Clostridiales	Clostridia	Firmicutes	Terrabacteria group
QIAGEN_13B_non-UTI	non-UTI	1146892	0.59%	1.92	Falsirhodobacter halotolerans	Falsirhodobacter	Rhodobacteraceae	Rhodobacterales	Alphaproteobacteria	Proteobacteria	
QIAGEN_13B_non-UTI	non-UTI	394230	0.55%	1.79	Deinococcus piscis	Deinococcus	Deinococcaceae	Deinococcales	Deinococci	Deinococcus-Thermus	Terrabacteria group
QIAGEN_13B_non-UTI	non-UTI	237576	0.42%	1.38	Oribacterium sinus	Oribacterium	Lachnospiraceae	Clostridiales	Clostridia	Firmicutes	Terrabacteria group
QIAGEN_13B_non-UTI	non-UTI	1379270	0.29%	0.96	Gemmatimonas phototrophica	Gemmatimonas	Gemmatimonadaceae	Gemmatimonadales	Gemmatimonadetes	Gemmatimonadetes	FCB group
QIAGEN_13B_non-UTI	non-UTI	46507	0.29%	0.96	Ezakiella coagulans	Ezakiella			Tissierellia	Firmicutes	Terrabacteria group
QIAGEN_13B_non-UTI	non-UTI	197614	0.29%	0.96	Streptococcus gallolyticus subsp. pasteurianus	Streptococcus	Streptococcaceae	Lactobacillales	Bacilli	Firmicutes	Terrabacteria group
QIAGEN_13B_non-UTI	non-UTI	109790	0.29%	0.96	Lactobacillus jensenii	Lactobacillus	Lactobacillaceae	Lactobacillales	Bacilli	Firmicutes	Terrabacteria group
QIAGEN_13B_non-UTI	non-UTI	187328	0.29%	0.96	Veillonella montpellierensis	Veillonella	Veillonellaceae	Veillonellales	Negativicutes	Firmicutes	Terrabacteria group
QIAGEN_13B_non-UTI	non-UTI	28125	0.29%	0.96	Prevotella bivia	Prevotella	Prevotellaceae	Bacteroidales	Bacteroidia	Bacteroidetes	FCB group
QIAGEN_13B_non-UTI	non-UTI	1465756	0.29%	0.96	Peptoniphilus grossensis	Peptoniphilus	Peptoniphilaceae	Tissierellales	Tissierellia	Firmicutes	Terrabacteria group
QIAGEN_13B_non-UTI	non-UTI	501496	0.29%	0.96	Porphyromonas bennonis	Porphyromonas	Porphyromonadaceae	Bacteroidales	Bacteroidia	Bacteroidetes	FCB group
QIAGEN_13B_non-UTI	non-UTI	938278	0.29%	0.96	Casaltella massiliensis	Casaltella	Clostridiales Family XIII. Incertae Sedis	Clostridiales	Clostridia	Firmicutes	Terrabacteria group
QIAGEN_13B_non-UTI	non-UTI	unmapped	0.00%	4.00							
QIAGEN_13B_non-UTI	non-UTI	mapped_unclassified	0.00%	14.00							
Promega_13A_non-UTI	non-UTI	712633	35.58%	183.95	Streptococcus sp. oral taxon 431	Streptococcus	Streptococcaceae	Lactobacillales	Bacilli	Firmicutes	Terrabacteria group
Promega_13A_non-UTI	non-UTI	562	24.99%	129.21	Escherichia coli	Escherichia	Enterobacteriaceae	Enterobacterales	Gammaproteobacteria	Proteobacteria	
Promega_13A_non-UTI	non-UTI	1747	11.05%	57.12	Cutibacterium acnes	Cutibacterium	Propionibacteriaceae	Propionibacteriales	Actinobacteria	Actinobacteria	Terrabacteria group
Promega_13A_non-UTI	non-UTI	1776391	10.86%	56.15	Romboutsia timonensis	Romboutsia	Peptostreptococcaceae	Clostridiales	Clostridia	Firmicutes	Terrabacteria group
Promega_13A_non-UTI	non-UTI	545	7.49%	38.73	Citrobacter koseri	Citrobacter	Enterobacteriaceae	Enterobacterales	Gammaproteobacteria	Proteobacteria	
Promega_13A_non-UTI	non-UTI	33011	3.00%	15.49	Cutibacterium granulosum	Cutibacterium	Propionibacteriaceae	Propionibacteriales	Actinobacteria	Actinobacteria	Terrabacteria group
Promega_13A_non-UTI	non-UTI	287	1.31%	6.78	Pseudomonas aeruginosa	Pseudomonas	Pseudomonadaceae	Pseudomonadales	Gammaproteobacteria	Proteobacteria	
Promega_13A_non-UTI	non-UTI	573	1.22%	6.32	Klebsiella pneumoniae	Klebsiella	Enterobacteriaceae	Enterobacterales	Gammaproteobacteria	Proteobacteria	
Promega_13A_non-UTI	non-UTI	1351	1.12%	5.81	Enterococcus faecalis	Enterococcus	Enterococcaceae	Lactobacillales	Bacilli	Firmicutes	Terrabacteria group
Promega_13A_non-UTI	non-UTI	1837344	0.81%	4.19	Brevilactibacter sinopodophylli	Brevilactibacter	Propionibacteriaceae	Propionibacteriales	Actinobacteria	Actinobacteria	Terrabacteria group
Promega_13A_non-UTI	non-UTI	47770	0.37%	1.94	Lactobacillus crispatus	Lactobacillus	Lactobacillaceae	Lactobacillales	Bacilli	Firmicutes	Terrabacteria group
Promega_13A_non-UTI	non-UTI	38304	0.37%	1.94	Corynebacterium tuberculostearicum	Corynebacterium	Corynebacteriaceae	Corynebacteriales	Actinobacteria	Actinobacteria	Terrabacteria group
Promega_13A_non-UTI	non-UTI	433330	0.31%	1.62	Propioniciclava tarda	Propioniciclava	Propionibacteriaceae	Propionibacteriales	Actinobacteria	Actinobacteria	Terrabacteria group
Promega_13A_non-UTI	non-UTI	28901	0.19%	0.98	Salmonella enterica	Salmonella	Enterobacteriaceae	Enterobacterales	Gammaproteobacteria	Proteobacteria	
Promega_13A_non-UTI	non-UTI	33012	0.19%	0.97	Propionimicrobium lymphophilum	Propionimicrobium	Propionibacteriaceae	Propionibacteriales	Actinobacteria	Actinobacteria	Terrabacteria group
Promega_13A_non-UTI	non-UTI	1261	0.19%	0.97	Peptostreptococcus anaerobius	Peptostreptococcus	Peptostreptococcaceae	Clostridiales	Clostridia	Firmicutes	Terrabacteria group
Promega_13A_non-UTI	non-UTI	669455	0.19%	0.97	Sediminibacterium magnilacihabitans	Sediminibacterium	Chitinophagaceae	Chitinophagales	Chitinophagia	Bacteroidetes	FCB group
Promega_13A_non-UTI	non-UTI	1311	0.19%	0.97	Streptococcus agalactiae	Streptococcus	Streptococcaceae	Lactobacillales	Bacilli	Firmicutes	Terrabacteria group
Promega_13A_non-UTI	non-UTI	827	0.19%	0.97	Campylobacter ureolyticus	Campylobacter	Campylobacteraceae	Campylobacterales	Epsilonproteobacteria	Proteobacteria	
Promega_13A_non-UTI	non-UTI	2081703	0.19%	0.97	Peptostreptococcaceae bacterium oral taxon 929		Peptostreptococcaceae	Clostridiales	Clostridia	Firmicutes	Terrabacteria group
Promega_13A_non-UTI	non-UTI	501496	0.19%	0.97	Porphyromonas bennonis	Porphyromonas	Porphyromonadaceae	Bacteroidales	Bacteroidia	Bacteroidetes	FCB group
Promega_13A_non-UTI	non-UTI	unmapped	0.00%	7.00							
Promega_13A_non-UTI	non-UTI	mapped_unclassified	0.00%	17.00							
PC_13A_non-UTI	non-UTI	2578113	30.33%	1 782.29	Bradyrhizobium sp. KBS0725	Bradyrhizobium	Bradyrhizobiaceae	Rhizobiales	Alphaproteobacteria	Proteobacteria	
PC_13A_non-UTI	non-UTI	2578114	30.33%	1 782.29	Bradyrhizobium sp. KBS0727	Bradyrhizobium	Bradyrhizobiaceae	Rhizobiales	Alphaproteobacteria	Proteobacteria	
PC_13A_non-UTI	non-UTI	1162689	11.25%	661.00	Sediminibacterium lactis	Sediminibacterium	Chitinophagaceae	Chitinophagales	Chitinophagia	Bacteroidetes	FCB group
PC_13A_non-UTI	non-UTI	674703	10.75%	631.80	Rhodoplanes sp. Z2-YC6860	Rhodoplanes	Hyphomicrobiaceae	Rhizobiales	Alphaproteobacteria	Proteobacteria	
PC_13A_non-UTI	non-UTI	1982110	7.55%	443.91	Hymenobacter profundi	Hymenobacter	Hymenobacteraceae	Cytophagales	Cytophagia	Bacteroidetes	FCB group
PC_13A_non-UTI	non-UTI	1295366	1.95%	114.87	Mesorhizobium soli	Mesorhizobium	Phyllobacteriaceae	Rhizobiales	Alphaproteobacteria	Proteobacteria	
PC_13A_non-UTI	non-UTI	34062	1.52%	89.56	Moraxella osloensis	Moraxella	Moraxellaceae	Pseudomonadales	Gammaproteobacteria	Proteobacteria	
PC_13A_non-UTI	non-UTI	562	1.37%	80.80	Escherichia coli	Escherichia	Enterobacteriaceae	Enterobacterales	Gammaproteobacteria	Proteobacteria	
PC_13A_non-UTI	non-UTI	1747	1.04%	61.33	Cutibacterium acnes	Cutibacterium	Propionibacteriaceae	Propionibacteriales	Actinobacteria	Actinobacteria	Terrabacteria group
PC_13A_non-UTI	non-UTI	287	1.04%	61.33	Pseudomonas aeruginosa	Pseudomonas	Pseudomonadaceae	Pseudomonadales	Gammaproteobacteria	Proteobacteria	
PC_13A_non-UTI	non-UTI	2565555	0.68%	39.91	Sphingomonas sp. PAMC26645	Sphingomonas	Sphingomonadaceae	Sphingomonadales	Alphaproteobacteria	Proteobacteria	
PC_13A_non-UTI	non-UTI	988946	0.55%	32.13	Loriellopsis cavernicola	Loriellopsis	Symphyonemataceae	Nostocales		Cyanobacteria	Terrabacteria group
PC_13A_non-UTI	non-UTI	1064518	0.43%	25.31	Noviherbaspirillum soli	Noviherbaspirillum	Oxalobacteraceae	Burkholderiales	Betaproteobacteria	Proteobacteria	
PC_13A_non-UTI	non-UTI	1418	0.31%	18.50	Bacillus mannanilyticus	Bacillus	Bacillaceae	Bacillales	Bacilli	Firmicutes	Terrabacteria group
PC_13A_non-UTI	non-UTI	269673	0.28%	16.55	Sporolactobacillus terrae	Sporolactobacillus	Sporolactobacillaceae	Bacillales	Bacilli	Firmicutes	Terrabacteria group
PC_13A_non-UTI	non-UTI	280333	0.22%	13.02	Bradyrhizobium pachyrhizi	Bradyrhizobium	Bradyrhizobiaceae	Rhizobiales	Alphaproteobacteria	Proteobacteria	
PC_13A_non-UTI	non-UTI	1215031	0.22%	12.66	Neobacillus thermocopriae	Neobacillus	Bacillaceae	Bacillales	Bacilli	Firmicutes	Terrabacteria group
PC_13A_non-UTI	non-UTI	2267537	0.17%	9.73	Roseomonas aeriglobus	Roseomonas	Acetobacteraceae	Rhodospirillales	Alphaproteobacteria	Proteobacteria	
PC_13A_non-UTI	non-UTI	unmapped	0.00%	4.00							
PC_13A_non-UTI	non-UTI	mapped_unclassified	0.00%	244.00							
IL-DEx_C1_control	control	669455	62.46%	9 776.89	Sediminibacterium magnilacihabitans	Sediminibacterium	Chitinophagaceae	Chitinophagales	Chitinophagia	Bacteroidetes	FCB group
IL-DEx_C1_control	control	1290	24.65%	3 857.63	Staphylococcus hominis	Staphylococcus	Staphylococcaceae	Bacillales	Bacilli	Firmicutes	Terrabacteria group
IL-DEx_C1_control	control	573	5.15%	805.33	Klebsiella pneumoniae	Klebsiella	Enterobacteriaceae	Enterobacterales	Gammaproteobacteria	Proteobacteria	
IL-DEx_C1_control	control	1352	2.20%	343.89	Enterococcus faecium	Enterococcus	Enterococcaceae	Lactobacillales	Bacilli	Firmicutes	Terrabacteria group
IL-DEx_C1_control	control	488447	1.30%	204.00	Burkholderia contaminans	Burkholderia	Burkholderiaceae	Burkholderiales	Betaproteobacteria	Proteobacteria	
IL-DEx_C1_control	control	1379270	1.04%	162.23	Gemmatimonas phototrophica	Gemmatimonas	Gemmatimonadaceae	Gemmatimonadales	Gemmatimonadetes	Gemmatimonadetes	FCB group
IL-DEx_C1_control	control	28037	0.87%	136.00	Streptococcus mitis	Streptococcus	Streptococcaceae	Lactobacillales	Bacilli	Firmicutes	Terrabacteria group
IL-DEx_C1_control	control	562	0.64%	100.06	Escherichia coli	Escherichia	Enterobacteriaceae	Enterobacterales	Gammaproteobacteria	Proteobacteria	
IL-DEx_C1_control	control	34062	0.58%	91.32	Moraxella osloensis	Moraxella	Moraxellaceae	Pseudomonadales	Gammaproteobacteria	Proteobacteria	
IL-DEx_C1_control	control	1230389	0.49%	76.74	Reyranella soli	Reyranella		Rhodospirillales	Alphaproteobacteria	Proteobacteria	
IL-DEx_C1_control	control	1235997	0.24%	37.66	Hydrobacter penzbergensis	Hydrobacter	Chitinophagaceae	Chitinophagales	Chitinophagia	Bacteroidetes	FCB group
IL-DEx_C1_control	control	1747	0.17%	27.20	Cutibacterium acnes	Cutibacterium	Propionibacteriaceae	Propionibacteriales	Actinobacteria	Actinobacteria	Terrabacteria group
IL-DEx_C1_control	control	1288393	0.13%	20.40	Corynebacterium pilbarense	Corynebacterium	Corynebacteriaceae	Corynebacteriales	Actinobacteria	Actinobacteria	Terrabacteria group
IL-DEx_C1_control	control	1351	0.08%	12.63	Enterococcus faecalis	Enterococcus	Enterococcaceae	Lactobacillales	Bacilli	Firmicutes	Terrabacteria group
IL-DEx_C1_control	control	unmapped	0.00%	17.00							
IL-DEx_C1_control	control	mapped_unclassified	0.00%	476.00							
QIAGEN_C1_control	control	1290	43.87%	89.49	Staphylococcus hominis	Staphylococcus	Staphylococcaceae	Bacillales	Bacilli	Firmicutes	Terrabacteria group
QIAGEN_C1_control	control	562	23.34%	47.61	Escherichia coli	Escherichia	Enterobacteriaceae	Enterobacterales	Gammaproteobacteria	Proteobacteria	
QIAGEN_C1_control	control	1776391	22.17%	45.23	Romboutsia timonensis	Romboutsia	Peptostreptococcaceae	Clostridiales	Clostridia	Firmicutes	Terrabacteria group
QIAGEN_C1_control	control	1351	2.83%	5.77	Enterococcus faecalis	Enterococcus	Enterococcaceae	Lactobacillales	Bacilli	Firmicutes	Terrabacteria group
QIAGEN_C1_control	control	545	1.89%	3.85	Citrobacter koseri	Citrobacter	Enterobacteriaceae	Enterobacterales	Gammaproteobacteria	Proteobacteria	
QIAGEN_C1_control	control	573	1.62%	3.31	Klebsiella pneumoniae	Klebsiella	Enterobacteriaceae	Enterobacterales	Gammaproteobacteria	Proteobacteria	
QIAGEN_C1_control	control	1311	0.94%	1.92	Streptococcus agalactiae	Streptococcus	Streptococcaceae	Lactobacillales	Bacilli	Firmicutes	Terrabacteria group
QIAGEN_C1_control	control	28901	0.51%	1.05	Salmonella enterica	Salmonella	Enterobacteriaceae	Enterobacterales	Gammaproteobacteria	Proteobacteria	
QIAGEN_C1_control	control	507750	0.47%	0.96	Peptoniphilus duerdenii	Peptoniphilus	Peptoniphilaceae	Tissierellales	Tissierellia	Firmicutes	Terrabacteria group
QIAGEN_C1_control	control	287	0.47%	0.96	Pseudomonas aeruginosa	Pseudomonas	Pseudomonadaceae	Pseudomonadales	Gammaproteobacteria	Proteobacteria	
QIAGEN_C1_control	control	294	0.47%	0.96	Pseudomonas fluorescens	Pseudomonas	Pseudomonadaceae	Pseudomonadales	Gammaproteobacteria	Proteobacteria	
QIAGEN_C1_control	control	1978467	0.47%	0.96	Pseudomonas sp. AK6U	Pseudomonas	Pseudomonadaceae	Pseudomonadales	Gammaproteobacteria	Proteobacteria	
QIAGEN_C1_control	control	1308	0.47%	0.96	Streptococcus thermophilus	Streptococcus	Streptococcaceae	Lactobacillales	Bacilli	Firmicutes	Terrabacteria group
QIAGEN_C1_control	control	827	0.47%	0.96	Campylobacter ureolyticus	Campylobacter	Campylobacteraceae	Campylobacterales	Epsilonproteobacteria	Proteobacteria	
QIAGEN_C1_control	control	unmapped	0.00%	98.00							
QIAGEN_C1_control	control	mapped_unclassified	0.00%	8.00							
Promega_C1_control	control	1308	53.28%	9 552.37	Streptococcus thermophilus	Streptococcus	Streptococcaceae	Lactobacillales	Bacilli	Firmicutes	Terrabacteria group
Promega_C1_control	control	1358	38.50%	6 903.44	Lactococcus lactis	Lactococcus	Streptococcaceae	Lactobacillales	Bacilli	Firmicutes	Terrabacteria group
Promega_C1_control	control	241425	2.15%	385.10	Crinalium epipsammum	Crinalium	Gomontiellaceae	Oscillatoriales		Cyanobacteria	Terrabacteria group
Promega_C1_control	control	1351	1.06%	189.27	Enterococcus faecalis	Enterococcus	Enterococcaceae	Lactobacillales	Bacilli	Firmicutes	Terrabacteria group

Promega_C1_control	control	1590	0.86%	154.08	Lactiplantibacillus plantarum	Lactiplantibacillus	Lactobacillaceae	Lactobacillales	Bacilli	Firmicutes	Terrabacteria group
Promega_C1_control	control	31998	0.77%	137.91	Methylobacterium radiotolerans	Methylobacterium	Methylobacteriaceae	Rhizobiales	Alphaproteobacteria	Proteobacteria	
Promega_C1_control	control	562	0.55%	97.96	Escherichia coli	Escherichia	Enterobacteriaceae	Enterobacterales	Gammaproteobacteria	Proteobacteria	
Promega_C1_control	control	137732	0.46%	81.79	Granulicatella elegans	Granulicatella	Carnobacteriaceae	Lactobacillales	Bacilli	Firmicutes	Terrabacteria group
Promega_C1_control	control	712633	0.44%	78.94	Streptococcus sp. oral taxon 431	Streptococcus	Streptococcaceae	Lactobacillales	Bacilli	Firmicutes	Terrabacteria group
Promega_C1_control	control	1747	0.43%	77.99	Cutibacterium acnes	Cutibacterium	Propionibacteriaceae	Propionibacteriales	Actinobacteria	Actinobacteria	Terrabacteria group
Promega_C1_control	control	34062	0.28%	49.46	Moraxella osloensis	Moraxella	Moraxellaceae	Pseudomonadales	Gammaproteobacteria	Proteobacteria	
Promega_C1_control	control	1433513	0.22%	39.95	Streptococcus gwangjuense	Streptococcus	Streptococcaceae	Lactobacillales	Bacilli	Firmicutes	Terrabacteria group
Promega_C1_control	control	1282	0.20%	36.14	Staphylococcus epidermidis	Staphylococcus	Staphylococcaceae	Bacillales	Bacilli	Firmicutes	Terrabacteria group
Promega_C1_control	control	1173026	0.17%	30.53	Gloeocapsa sp. PCC 7428	Gloeocapsa	Chroococcaceae	Chroococcales		Cyanobacteria	Terrabacteria group
Promega_C1_control	control	1961362	0.13%	23.78	Sphingomonas sp. NIC1	Sphingomonas	Sphingomonadaceae	Sphingomonadales	Alphaproteobacteria	Proteobacteria	
Promega_C1_control	control	288426	0.12%	20.92	Paracoccus marinus	Paracoccus	Rhodobacteraceae	Rhodobacterales	Alphaproteobacteria	Proteobacteria	
Promega_C1_control	control	106654	0.11%	19.97	Acinetobacter nosocomialis	Acinetobacter	Moraxellaceae	Pseudomonadales	Gammaproteobacteria	Proteobacteria	
Promega_C1_control	control	46124	0.10%	17.12	Granulicatella adiacens	Granulicatella	Carnobacteriaceae	Lactobacillales	Bacilli	Firmicutes	Terrabacteria group
Promega_C1_control	control	1536771	0.07%	12.36	Paenibacillus sp. FSL R5-0912	Paenibacillus	Paenibacillaceae	Bacillales	Bacilli	Firmicutes	Terrabacteria group
Promega_C1_control	control	37636	0.06%	11.41	Thermus scotoductus	Thermus	Thermaceae	Thermales	Deinococci	Deinococcus-Thermus	Terrabacteria group
Promega_C1_control	control	374425	0.05%	9.51	Methylobacterium komagatae	Methylobacterium	Methylobacteriaceae	Rhizobiales	Alphaproteobacteria	Proteobacteria	
Promega_C1_control	control	unmapped	0.00%	4.00							
Promega_C1_control	control	mapped_unclassified	0.00%	971.00							
PC_C1_control	control	2578113	15.82%	131.01	Bradyrhizobium sp. KBS0725	Bradyrhizobium	Bradyrhizobiaceae	Rhizobiales	Alphaproteobacteria	Proteobacteria	
PC_C1_control	control	2578114	15.82%	131.01	Bradyrhizobium sp. KBS0727	Bradyrhizobium	Bradyrhizobiaceae	Rhizobiales	Alphaproteobacteria	Proteobacteria	
PC_C1_control	control	1162689	15.77%	130.54	Sediminibacterium lactis	Sediminibacterium	Chitinophagaceae	Chitinophagales	Chitinophagia	Bacteroidetes	FCB group
PC_C1_control	control	674703	11.51%	95.28	Rhodoplanes sp. Z2-YC6860	Rhodoplanes	Hyphomicrobiaceae	Rhizobiales	Alphaproteobacteria	Proteobacteria	
PC_C1_control	control	1295366	8.75%	72.41	Mesorhizobium soli	Mesorhizobium	Phyllobacteriaceae	Rhizobiales	Alphaproteobacteria	Proteobacteria	
PC_C1_control	control	287	6.90%	57.17	Pseudomonas aeruginosa	Pseudomonas	Pseudomonadaceae	Pseudomonadales	Gammaproteobacteria	Proteobacteria	
PC_C1_control	control	562	6.77%	56.08	Escherichia coli	Escherichia	Enterobacteriaceae	Enterobacterales	Gammaproteobacteria	Proteobacteria	
PC_C1_control	control	147645	4.72%	39.07	Paracoccus yeei	Paracoccus	Rhodobacteraceae	Rhodobacterales	Alphaproteobacteria	Proteobacteria	
PC_C1_control	control	1290	2.42%	20.01	Staphylococcus hominis	Staphylococcus	Staphylococcaceae	Bacillales	Bacilli	Firmicutes	Terrabacteria group
PC_C1_control	control	374426	2.07%	17.15	Methylobacterium persicinum	Methylobacterium	Methylobacteriaceae	Rhizobiales	Alphaproteobacteria	Proteobacteria	
PC_C1_control	control	190721	2.07%	17.15	Ralstonia insidiosa	Ralstonia	Burkholderiaceae	Burkholderiales	Betaproteobacteria	Proteobacteria	
PC_C1_control	control	1351	1.38%	11.43	Enterococcus faecalis	Enterococcus	Enterococcaceae	Lactobacillales	Bacilli	Firmicutes	Terrabacteria group
PC_C1_control	control	573	1.17%	9.67	Klebsiella pneumoniae	Klebsiella	Enterobacteriaceae	Enterobacterales	Gammaproteobacteria	Proteobacteria	
PC_C1_control	control	160791	0.96%	7.97	Sphingomonas wittichii	Sphingomonas	Sphingomonadaceae	Sphingomonadales	Alphaproteobacteria	Proteobacteria	
PC_C1_control	control	54007	0.92%	7.62	Anaerococcus octavius	Anaerococcus	Peptoniphilaceae	Tissierellales	Tissierellia	Firmicutes	Terrabacteria group
PC_C1_control	control	288000	0.58%	4.77	Bradyrhizobium sp. BTAi1	Bradyrhizobium	Bradyrhizobiaceae	Rhizobiales	Alphaproteobacteria	Proteobacteria	
PC_C1_control	control	1166337	0.37%	3.06	Sphingomonas jatrophae	Sphingomonas	Sphingomonadaceae	Sphingomonadales	Alphaproteobacteria	Proteobacteria	
PC_C1_control	control	861534	0.35%	2.88	Sphingomonas formosensis	Sphingomonas	Sphingomonadaceae	Sphingomonadales	Alphaproteobacteria	Proteobacteria	
PC_C1_control	control	1241761	0.31%	2.61	Sphingomonas yantingensis	Sphingomonas	Sphingomonadaceae	Sphingomonadales	Alphaproteobacteria	Proteobacteria	
PC_C1_control	control	2081703	0.23%	1.91	Peptostreptococcaceae bacterium oral taxon 929		Peptostreptococcaceae	Clostridiales	Clostridia	Firmicutes	Terrabacteria group
PC_C1_control	control	46507	0.23%	1.91	Ezakiella coagulans	Ezakiella			Tissierellia	Firmicutes	Terrabacteria group
PC_C1_control	control	545	0.23%	1.91	Citrobacter koseri	Citrobacter	Enterobacteriaceae	Enterobacterales	Gammaproteobacteria	Proteobacteria	
PC_C1_control	control	1030157	0.19%	1.59	Sphingomonas sp. KC8	Sphingomonas	Sphingomonadaceae	Sphingomonadales	Alphaproteobacteria	Proteobacteria	
PC_C1_control	control	884684	0.12%	0.95	Mageeibacillus indolicus	Mageeibacillus	Hungateiclostridiaceae	Clostridiales	Clostridia	Firmicutes	Terrabacteria group
PC_C1_control	control	1358	0.12%	0.95	Lactococcus lactis	Lactococcus	Streptococcaceae	Lactobacillales	Bacilli	Firmicutes	Terrabacteria group
PC_C1_control	control	33032	0.12%	0.95	Anaerococcus lactolyticus	Anaerococcus	Peptoniphilaceae	Tissierellales	Tissierellia	Firmicutes	Terrabacteria group
PC_C1_control	control	28125	0.12%	0.95	Prevotella bivia	Prevotella	Prevotellaceae	Bacteroidales	Bacteroidia	Bacteroidetes	FCB group
PC_C1_control	control	unmapped	0.00%	53.00							
PC_C1_control	control	mapped_unclassified	0.00%	42.00							
IL-DEx_TE_buffer_Elution_b	Elution buffer	562	61.95%	91.06	Escherichia coli	Escherichia	Enterobacteriaceae	Enterobacterales	Gammaproteobacteria	Proteobacteria	
IL-DEx_TE_buffer_Elution_b	Elution buffer	1747	8.67%	12.74	Cutibacterium acnes	Cutibacterium	Propionibacteriaceae	Propionibacteriales	Actinobacteria	Actinobacteria	Terrabacteria group
IL-DEx_TE_buffer_Elution_b	Elution buffer	1351	6.00%	8.82	Enterococcus faecalis	Enterococcus	Enterococcaceae	Lactobacillales	Bacilli	Firmicutes	Terrabacteria group
IL-DEx_TE_buffer_Elution_b	Elution buffer	243690	6.00%	8.82	Methylobacterium goesingense	Methylobacterium	Methylobacteriaceae	Rhizobiales	Alphaproteobacteria	Proteobacteria	
IL-DEx_TE_buffer_Elution_b	Elution buffer	287	3.33%	4.90	Pseudomonas aeruginosa	Pseudomonas	Pseudomonadaceae	Pseudomonadales	Gammaproteobacteria	Proteobacteria	
IL-DEx_TE_buffer_Elution_b	Elution buffer	573	2.72%	3.99	Klebsiella pneumoniae	Klebsiella	Enterobacteriaceae	Enterobacterales	Gammaproteobacteria	Proteobacteria	
IL-DEx_TE_buffer_Elution_b	Elution buffer	1402	2.44%	3.59	Bacillus licheniformis	Bacillus	Bacillaceae	Bacillales	Bacilli	Firmicutes	Terrabacteria group
IL-DEx_TE_buffer_Elution_b	Elution buffer	1856406	2.44%	3.59	Bacillus sp. H15-1	Bacillus	Bacillaceae	Bacillales	Bacilli	Firmicutes	Terrabacteria group
IL-DEx_TE_buffer_Elution_b	Elution buffer	2026248	2.44%	3.59	Bacillus sp. 1s-1	Bacillus	Bacillaceae	Bacillales	Bacilli	Firmicutes	Terrabacteria group
IL-DEx_TE_buffer_Elution_b	Elution buffer	545	1.33%	1.96	Citrobacter koseri	Citrobacter	Enterobacteriaceae	Enterobacterales	Gammaproteobacteria	Proteobacteria	
IL-DEx_TE_buffer_Elution_b	Elution buffer	208962	0.67%	0.98	Escherichia albertii	Escherichia	Enterobacteriaceae	Enterobacterales	Gammaproteobacteria	Proteobacteria	
IL-DEx_TE_buffer_Elution_b	Elution buffer	2572087	0.67%	0.98	Candidatus Saccharibacteria bacterium oral taxon 955					Candidatus Saccharibacteria	Bacteria candidate phyla
IL-DEx_TE_buffer_Elution_b	Elution buffer	197614	0.67%	0.98	Streptococcus gallolyticus subsp. pasteurianus	Streptococcus	Streptococcaceae	Lactobacillales	Bacilli	Firmicutes	Terrabacteria group
IL-DEx_TE_buffer_Elution_b	Elution buffer	1261	0.67%	0.98	Peptostreptococcus anaerobius	Peptostreptococcus	Peptostreptococcaceae	Clostridiales	Clostridia	Firmicutes	Terrabacteria group
IL-DEx_TE_buffer_Elution_b	Elution buffer	unmapped	0.00%	1.00							
IL-DEx_TE_buffer_Elution_b	Elution buffer	mapped_unclassified	0.00%	3.00							
QIAGEN_Elution_Elution_bu	Elution buffer	34062	55.66%	4 466.38	Moraxella osloensis	Moraxella	Moraxellaceae	Pseudomonadales	Gammaproteobacteria	Proteobacteria	
QIAGEN_Elution_Elution_bu	Elution buffer	669455	12.53%	1 005.57	Sediminibacterium magnilacihabitans	Sediminibacterium	Chitinophagaceae	Chitinophagales	Chitinophagia	Bacteroidetes	FCB group
QIAGEN_Elution_Elution_bu	Elution buffer	1282	5.39%	432.41	Staphylococcus epidermidis	Staphylococcus	Staphylococcaceae	Bacillales	Bacilli	Firmicutes	Terrabacteria group
QIAGEN_Elution_Elution_bu	Elution buffer	1270	3.33%	266.96	Micrococcus luteus	Micrococcus	Micrococcaceae	Micrococcales	Actinobacteria	Actinobacteria	Terrabacteria group
QIAGEN_Elution_Elution_bu	Elution buffer	993416	3.32%	266.15	Micrococcus cohnii	Micrococcus	Micrococcaceae	Micrococcales	Actinobacteria	Actinobacteria	Terrabacteria group
QIAGEN_Elution_Elution_bu	Elution buffer	29382	2.84%	227.94	Staphylococcus cohnii	Staphylococcus	Staphylococcaceae	Bacillales	Bacilli	Firmicutes	Terrabacteria group
QIAGEN_Elution_Elution_bu	Elution buffer	1290	2.43%	194.62	Staphylococcus hominis	Staphylococcus	Staphylococcaceae	Bacillales	Bacilli	Firmicutes	Terrabacteria group
QIAGEN_Elution_Elution_bu	Elution buffer	29388	2.41%	193.62	Staphylococcus capitis	Staphylococcus	Staphylococcaceae	Bacillales	Bacilli	Firmicutes	Terrabacteria group
QIAGEN_Elution_Elution_bu	Elution buffer	1396	1.88%	150.64	Bacillus cereus	Bacillus	Bacillaceae	Bacillales	Bacilli	Firmicutes	Terrabacteria group
QIAGEN_Elution_Elution_bu	Elution buffer	1747	1.80%	144.77	Cutibacterium acnes	Cutibacterium	Propionibacteriaceae	Propionibacteriales	Actinobacteria	Actinobacteria	Terrabacteria group
QIAGEN_Elution_Elution_bu	Elution buffer	29385	1.43%	114.43	Staphylococcus saprophyticus	Staphylococcus	Staphylococcaceae	Bacillales	Bacilli	Firmicutes	Terrabacteria group
QIAGEN_Elution_Elution_bu	Elution buffer	138300	1.35%	108.24	Kytococcus schroeteri	Kytococcus	Dermacoccaceae	Micrococcales	Actinobacteria	Actinobacteria	Terrabacteria group
QIAGEN_Elution_Elution_bu	Elution buffer	562	1.13%	90.97	Escherichia coli	Escherichia	Enterobacteriaceae	Enterobacterales	Gammaproteobacteria	Proteobacteria	
QIAGEN_Elution_Elution_bu	Elution buffer	1230389	1.02%	82.17	Reyranella soli	Reyranella		Rhodospirillales	Alphaproteobacteria	Proteobacteria	
QIAGEN_Elution_Elution_bu	Elution buffer	38289	0.99%	79.23	Corynebacterium jeikeium	Corynebacterium	Corynebacteriaceae	Corynebacteriales	Actinobacteria	Actinobacteria	Terrabacteria group
QIAGEN_Elution_Elution_bu	Elution buffer	1276	0.93%	74.68	Kytococcus sedentarius	Kytococcus	Dermacoccaceae	Micrococcales	Actinobacteria	Actinobacteria	Terrabacteria group
QIAGEN_Elution_Elution_bu	Elution buffer	33011	0.48%	38.15	Cutibacterium granulosum	Cutibacterium	Propionibacteriaceae	Propionibacteriales	Actinobacteria	Actinobacteria	Terrabacteria group
QIAGEN_Elution_Elution_bu	Elution buffer	1283	0.43%	34.27	Staphylococcus haemolyticus	Staphylococcus	Staphylococcaceae	Bacillales	Bacilli	Firmicutes	Terrabacteria group
QIAGEN_Elution_Elution_bu	Elution buffer	1273	0.21%	16.63	Micrococcus lylae	Micrococcus	Micrococcaceae	Micrococcales	Actinobacteria	Actinobacteria	Terrabacteria group
QIAGEN_Elution_Elution_bu	Elution buffer	1351	0.16%	12.72	Enterococcus faecalis	Enterococcus	Enterococcaceae	Lactobacillales	Bacilli	Firmicutes	Terrabacteria group
QIAGEN_Elution_Elution_bu	Elution buffer	62629	0.16%	12.72	Corynebacterium thomssenii	Corynebacterium	Corynebacteriaceae	Corynebacteriales	Actinobacteria	Actinobacteria	Terrabacteria group
QIAGEN_Elution_Elution_bu	Elution buffer	479117	0.15%	11.74	Brevibacterium ravenspurgense	Brevibacterium	Brevibacteriaceae	Micrococcales	Actinobacteria	Actinobacteria	Terrabacteria group
QIAGEN_Elution_Elution_bu	Elution buffer	unmapped	0.00%	3.00							
QIAGEN_Elution_Elution_bu	Elution buffer	mapped_unclassified	0.00%	207.00							
Promega_Elution_Elution_b	Elution buffer	562	46.48%	51.60	Escherichia coli	Escherichia	Enterobacteriaceae	Enterobacterales	Gammaproteobacteria	Proteobacteria	
Promega_Elution_Elution_b	Elution buffer	54005	11.86%	13.17	Peptoniphilus harei	Peptoniphilus	Peptoniphilaceae	Tissierellales	Tissierellia	Firmicutes	Terrabacteria group
Promega_Elution_Elution_b	Elution buffer	34062	10.17%	11.29	Moraxella osloensis	Moraxella	Moraxellaceae	Pseudomonadales	Gammaproteobacteria	Proteobacteria	
Promega_Elution_Elution_b	Elution buffer	1002526	7.63%	8.47	Pseudomonas formosensis	Pseudomonas	Pseudomonadaceae	Pseudomonadales	Gammaproteobacteria	Proteobacteria	
Promega_Elution_Elution_b	Elution buffer	573	6.91%	7.67	Klebsiella pneumoniae	Klebsiella	Enterobacteriaceae	Enterobacterales	Gammaproteobacteria	Proteobacteria	
Promega_Elution_Elution_b	Elution buffer	1351	4.24%	4.70	Enterococcus faecalis	Enterococcus	Enterococcaceae	Lactobacillales	Bacilli	Firmicutes	Terrabacteria group
Promega_Elution_Elution_b	Elution buffer	545	2.54%	2.82	Citrobacter koseri	Citrobacter	Enterobacteriaceae	Enterobacterales	Gammaproteobacteria	Proteobacteria	
Promega_Elution_Elution_b	Elution buffer	1396	2.54%	2.82	Bacillus cereus	Bacillus	Bacillaceae	Bacillales	Bacilli	Firmicutes	Terrabacteria group
Promega_Elution_Elution_b	Elution buffer	1311	1.69%	1.88	Streptococcus agalactiae	Streptococcus	Streptococcaceae	Lactobacillales	Bacilli	Firmicutes	Terrabacteria group
Promega_Elution_Elution_b	Elution buffer	411577	0.85%	0.94	Anaerococcus murdochii	Anaerococcus	Peptoniphilaceae	Tissierellales	Tissierellia	Firmicutes	Terrabacteria group
Promega_Elution_Elution_b	Elution buffer	47715	0.85%	0.94	Lactobacillus rhamnosus	Lactobacillus	Lactobacillaceae	Lactobacillales	Bacilli	Firmicutes	Terrabacteria group
Promega_Elution_Elution_b	Elution buffer	1261	0.85%	0.94	Peptostreptococcus anaerobius	Peptostreptococcus	Peptostreptococcaceae	Clostridiales	Clostridia	Firmicutes	Terrabacteria group
Promega_Elution_Elution_b	Elution buffer	28125	0.85%	0.94	Prevotella bivia	Prevotella	Prevotellaceae	Bacteroidales	Bacteroidia	Bacteroidetes	FCB group
Promega_Elution_Elution_b	Elution buffer	1852374	0.85%	0.94	Ezakiella massiliensis	Ezakiella			Tissierellia	Firmicutes	Terrabacteria group
Promega_Elution_Elution_b	Elution buffer	1747	0.85%	0.94	Cutibacterium acnes	Cutibacterium	Propionibacteriaceae	Propionibacteriales	Actinobacteria	Actinobacteria	Terrabacteria group
Promega_Elution_Elution_b	Elution buffer	584	0.85%	0.94	Proteus mirabilis	Proteus	Morganellaceae	Enterobacterales	Gammaproteobacteria	Proteobacteria	
Promega_Elution_Elution_b	Elution buffer	unmapped	0.00%	4.00							
Promega_Elution_Elution_b	Elution buffer	mapped_unclassified	0.00%	8.00							
PC_Tris_Elution_buffer	Elution buffer	562	62.25%	50.42	Escherichia coli	Escherichia	Enterobacteriaceae	Enterobacterales	Gammaproteobacteria	Proteobacteria	
PC_Tris_Elution_buffer	Elution buffer	1351	6.17%	5.00	Enterococcus faecalis	Enterococcus	Enterococcaceae	Lactobacillales	Bacilli	Firmicutes	Terrabacteria group
PC_Tris_Elution_buffer	Elution buffer	573	5.66%	4.58	Klebsiella pneumoniae	Klebsiella	Enterobacteriaceae	Enterobacterales	Gammaproteobacteria	Proteobacteria	
PC_Tris_Elution_buffer	Elution buffer	34062	4.94%	4.00	Moraxella osloensis	Moraxella	Moraxellaceae	Pseudomonadales	Gammaproteobacteria	Proteobacteria	
PC_Tris_Elution_buffer	Elution buffer	545	4.94%	4.00	Citrobacter koseri	Citrobacter	Enterobacteriaceae	Enterobacterales	Gammaproteobacteria	Proteobacteria	
PC_Tris_Elution_buffer	Elution buffer	287	3.70%	3.00	Pseudomonas aeruginosa	Pseudomonas	Pseudomonadaceae	Pseudomonadales	Gammaproteobacteria	Proteobacteria	
PC_Tris_Elution_buffer	Elution buffer	46507	2.47%	2.00	Ezakiella coagulans	Ezakiella			Tissierellia	Firmicutes	Terrabacteria group
PC_Tris_Elution_buffer	Elution buffer	197614	1.23%	1.00	Streptococcus gallolyticus subsp. pasteurianus	Streptococcus	Streptococcaceae	Lactobacillales	Bacilli	Firmicutes	Terrabacteria group
PC_Tris_Elution_buffer	Elution buffer	1393034	1.23%	1.00	Atopobium deltae	Atopobium	Atopobiaceae	Coriobacteriales	Coriobacteriia	Actinobacteria	Terrabacteria group
PC_Tris_Elution_buffer	Elution buffer	187328	1.23%	1.00	Veillonella montpellierensis	Veillonella	Veillonellaceae	Veillonellales	Negativicutes	Firmicutes	Terrabacteria group
PC_Tris_Elution_buffer	Elution buffer	1261	1.23%	1.00	Peptostreptococcus anaerobius	Peptostreptococcus	Peptostreptococcaceae	Clostridiales	Clostridia	Firmicutes	Terrabacteria group
PC_Tris_Elution_buffer	Elution buffer	386414	1.23%	1.00	Prevotella timonensis	Prevotella	Prevotellaceae	Bacteroidales	Bacteroidia	Bacteroidetes	FCB group
PC_Tris_Elution_buffer	Elution buffer	147802	1.23%	1.00	Lactobacillus iners	Lactobacillus	Lactobacillaceae	Lactobacillales	Bacilli	Firmicutes	Terrabacteria group
PC_Tris_Elution_buffer	Elution buffer	28141	1.23%	1.00	Cronobacter sakazakii	Cronobacter	Enterobacteriaceae	Enterobacterales	Gammaproteobacteria	Proteobacteria	
PC_Tris_Elution_buffer	Elution buffer	1311	1.23%	1.00	Streptococcus agalactiae	Streptococcus	Streptococcaceae	Lactobacillales	Bacilli	Firmicutes	Terrabacteria group
PC_Tris_Elution_buffer	Elution buffer	unmapped	0.00%	1.00							
PC_Tris_Elution_buffer	Elution buffer	mapped_unclassified	0.00%	0.00							
Library_NTC_Nuclease-free	Nuclease-free water	562	71.59%	68.01	Escherichia coli	Escherichia	Enterobacteriaceae	Enterobacterales	Gammaproteobacteria	Proteobacteria	
Library_NTC_Nuclease-free	Nuclease-free water	573	9.24%	8.78	Klebsiella pneumoniae	Klebsiella	Enterobacteriaceae	Enterobacterales	Gammaproteobacteria	Proteobacteria	
Library_NTC_Nuclease-free	Nuclease-free water	1351	7.37%	7.00	Enterococcus faecalis	Enterococcus	Enterococcaceae	Lactobacillales	Bacilli	Firmicutes	Terrabacteria group
Library_NTC_Nuclease-free	Nuclease-free water	54005	2.11%	2.00	Peptoniphilus harei	Peptoniphilus	Peptoniphilaceae	Tissierellales	Tissierellia	Firmicutes	Terrabacteria group
Library_NTC_Nuclease-free	Nuclease-free water	545	2.11%	2.00	Citrobacter koseri	Citrobacter	Enterobacteriaceae	Enterobacterales	Gammaproteobacteria	Proteobacteria	
Library_NTC_Nuclease-free	Nuclease-free water	564	1.28%	1.21	Escherichia fergusonii	Escherichia	Enterobacteriaceae	Enterobacterales	Gammaproteobacteria	Proteobacteria	
Library_NTC_Nuclease-free	Nuclease-free water	501496	1.05%	1.00	Porphyromonas bennonis	Porphyromonas	Porphyromonadaceae	Bacteroidales	Bacteroidia	Bacteroidetes	FCB group
Library_NTC_Nuclease-free	Nuclease-free water	1311	1.05%	1.00	Streptococcus agalactiae	Streptococcus	Streptococcaceae	Lactobacillales	Bacilli	Firmicutes	Terrabacteria group
Library_NTC_Nuclease-free	Nuclease-free water	818	1.05%	1.00	Bacteroides thetaiotaomicron	Bacteroides	Bacteroidaceae	Bacteroidales	Bacteroidia	Bacteroidetes	FCB group
Library_NTC_Nuclease-free	Nuclease-free water	2081703	1.05%	1.00	Peptostreptococcaceae bacterium oral taxon 929		Peptostreptococcaceae	Clostridiales	Clostridia	Firmicutes	Terrabacteria group
Library_NTC_Nuclease-free	Nuclease-free water	1982110	1.05%	1.00	Hymenobacter profundi	Hymenobacter	Hymenobacteraceae	Cytophagales	Cytophagia	Bacteroidetes	FCB group
Library_NTC_Nuclease-free	Nuclease-free water	47770	1.05%	1.00	Lactobacillus crispatus	Lactobacillus	Lactobacillaceae	Lactobacillales	Bacilli	Firmicutes	Terrabacteria group
Library_NTC_Nuclease-free	Nuclease-free water	unmapped	0.00%	0.00							
Library_NTC_Nuclease-free	Nuclease-free water	mapped_unclassified	0.00%	2.00							
